# Supplementary material for: Arresting failure propagation in buildings through collapse isolation
Source: Nature. 2024 May 15;629(8012):592–6. doi: 10.1038/s41586-024-07268-5 (PMC11096105; doi:10.1038/s41586-024-07268-5)
Supplement: Supplementary file 1 — This file contains a supplementary test report that covers as-built building design, material properties, monitoring plan, structural response in phase 1 of testing and structural response in phase 2 of testing. [file 41586_2024_7268_MOESM1_ESM.pdf]

nature portfolio

https://doi.org/10.1038/s41586-024-07268-5

Supplementary information

Arresting failure propagation in buildings through collapse isolation

In the format provided by the
authors and unedited

SUPPLEMENTARY INFORMATION

February 2024

Index of contents

Introduction 3
Section 1: As-built building design 4
Section 2: Material properties 6
Section 3: Monitoring plan 7
Section 4: Structural response in Phase 1 of testing 11
Section 5: Structural response in Phase 2 of testing 21
References 33
Appendix: Detailed drawing of the as-built building design 34

Introduction

This test report was prepared as supplementary information for the main article. The report aims to elaborate in detail on the test campaign, including the building specimen, the tested material properties, the monitoring plan, and the main measurements recorded during Phase 1 and Phase 2 of testing.

The report is structured into five main sections:

- **S1. As-built building design**
- **S2. Material properties**
- **S3. Monitoring plan**
- **S4. Structural response in Phase 1 of testing**
- **S5. Structural response in Phase 2 of testing**

Section 1

As-built building design

The purpose-built specimen had two 2.6 m high floors with a 15 × 12 m floor plan. The longer span of the building consisted of 3 bays of 5 m span, whereas the shorter side was composed of 2 bays of 6 m span (**S-Fig. 1a**). We designed the specimen with 2.5 kN/m^2 dead load and 5.0 kN/m^2 live load according to Eurocodes suitable for use categories C3, C4, C5, D1 or D2 [1–7]. All the columns were precast with a dimension of 40 x 40 cm (detailed information about the column reinforcement configuration is provided in the Appendix) except in three column locations where they were made of "hanging" concrete columns (C8 and C11 shown in **S-Fig. 1b**) and a triple-hinged steel column (C12 shown in **S-Fig. 1c**) designed specifically to perform the column removal scenarios.

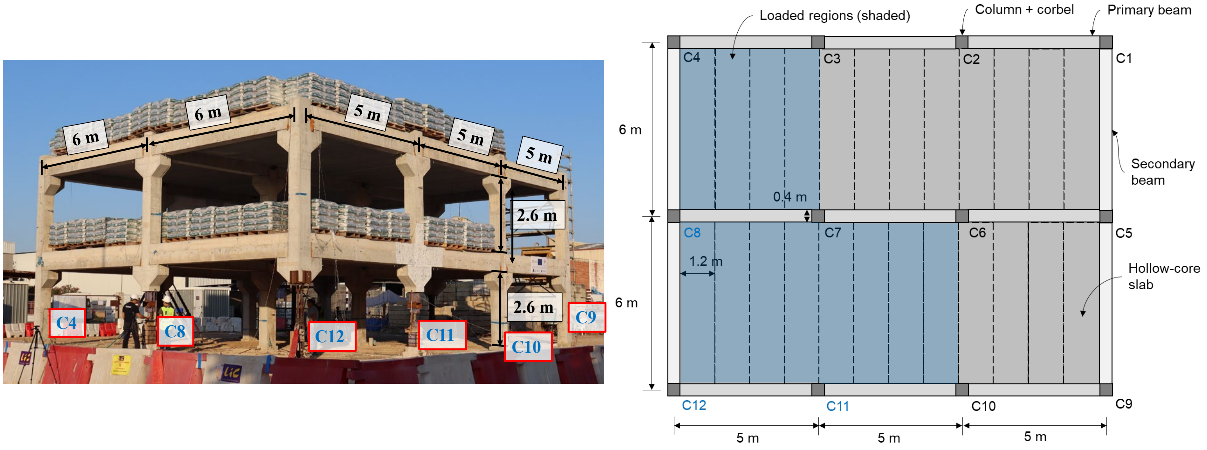

(a)

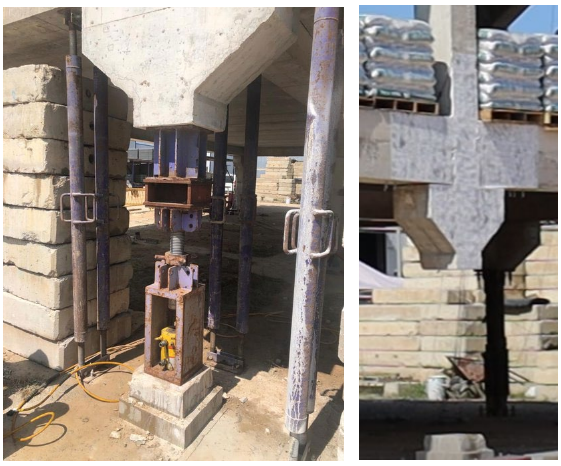

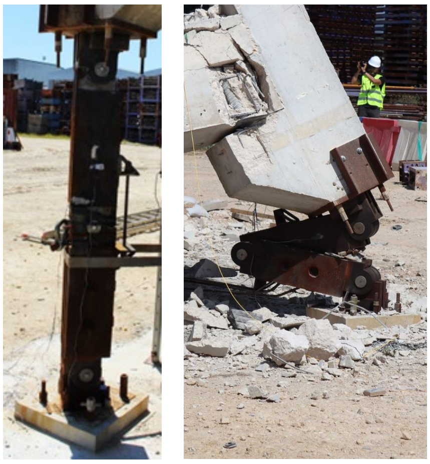

(b) (c)

Supplementary Figure 1. (a) Layout and geometries of the purpose-built precast specimen; (b) "Hanging" concrete columns (C8 and C11); (c) Three-hinged steel column (C12) before and after the removal process.

All the beams were partially precast with a depth of 61.5 cm (35 cm precast + 26.5 cm topping) and a width of 40 cm (**S-Fig. 2a**). These beams were supported on elastomeric pad bearings atop the column's corbel (**S-Fig. 2b**). The top continuity bars passed through the holes specially constructed in the precast column (**S-Fig. 2c**). These bars were installed continuously from edge to edge using rebar splicing at the internal span and anchored to the corner column using mechanical couplers. The beams' bottom side (the precast part) was connected to the corbels using 2-Φ20 mm dowel bars. Hollow core slabs with a total depth of 26.5 cm (20 cm precast + 6.5 cm topping) and a unit width of 1.2 m were used as the floor system, spanning 6 m between the primary beams (**S-Fig. 2d**). We used 12 mm diameter ties to connect two adjacent hollow core units and between the beams and the hollow core units. We also designed additional top reinforcement (503 mm^2/m) bridging the two bays of the hollow core system passing through the top of the internal beams. Detailed construction drawings of all these structural components are included in the Appendix of this test report. The construction process of the building specimen is shown in Supplementary Video 4.

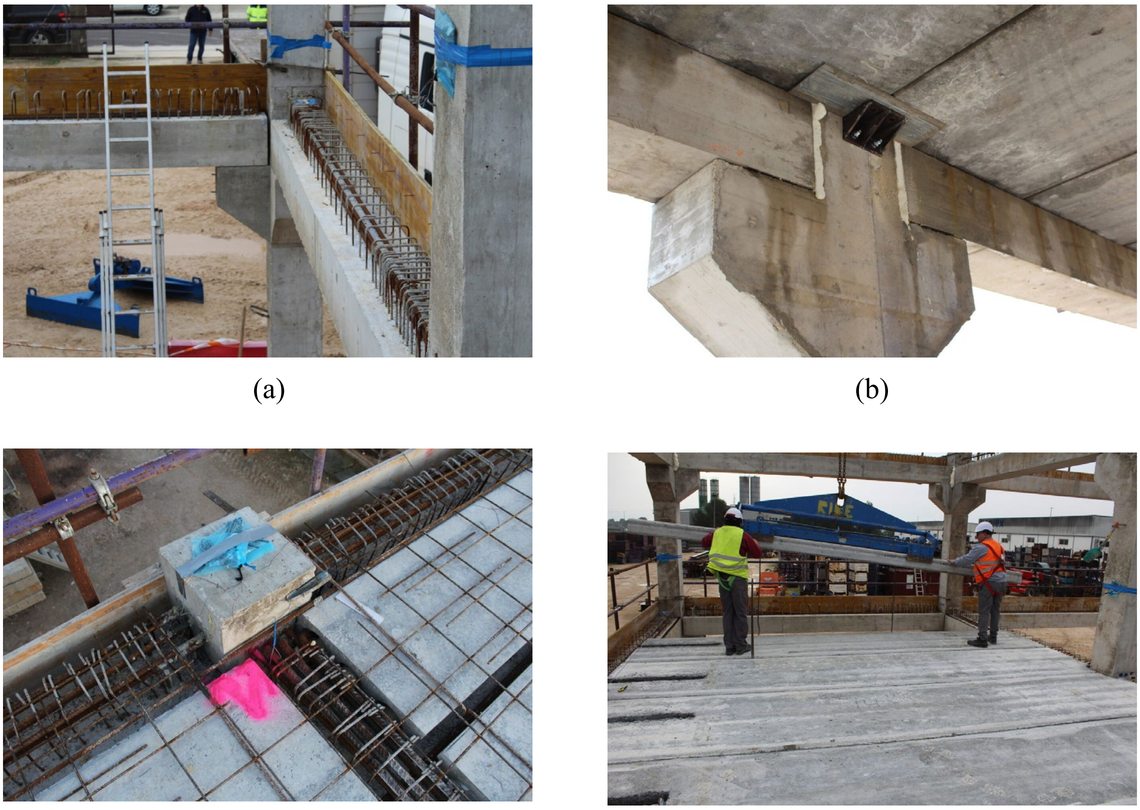

(a) (b) (c) (d)

Supplementary Figure 2. (a) Precast beams; (b) Beam-column connections using corbels; (c) Continuity bars in the beams; (d) Hollow-core slab systems.

Section 2
Material properties

The material characterisation was performed to identify the properties of the concrete and the reinforcing bars used to construct the building specimen. The test of concrete materials comprised compressive and tensile tests and a determination of the elastic modulus. These tests were performed according to EN 12390-3, EN 12390-6 and EN 12390-13, respectively. The reinforcing bars were tested through a typical uniaxial tensile test of a bare bar (in air) according to [EN ISO 15630-1] to determine the initial elastic modulus, yield strength, ultimate strength, peak and rupture strain. The summary of the material properties is shown in **Supplementary Table 1** below.

Supplementary Table 1. Mechanical properties of the concrete and steel reinforcement of different elements.

Mechanical property	Element	Results
Compressive Strength [MPa] *3 concrete cylinders per element; 300x150mm (EN 12390-3)*	Columns C2, C6, C7, C10, C11	33.9
	Columns C1, C3, C4, C5, C8, C9, C12	36.3
	1st slab beams	37.1
	2nd slab beams	39.6
	Topping	35.5
Elastic Modulus [MPa] *3 concrete cylinders per element; 300x150mm (EN 12390-13)*	Columns C2, C6, C7, C10, C11	31,109
	Columns C1, C3, C4, C5, C8, C9, C12	29,235
	1st slab beams	33,032
	2nd slab beams	37,605
	Topping	35,318
Tensile Strength [MPa] *3 concrete cylinders per element; 300x150mm (EN 12390-6)*	Columns C2, C6, C7, C10, C11	2.43
	Columns C1, C3, C4, C5, C8, C9, C12	2.97
	1st slab beams	2.50
	2nd slab beams	3.03
	Topping	3.27
Reinforcement Yield Strength [MPa] *(EN ISO 15630-1)*	Φ12 (4 specimens)	590
	Φ25 (4 specimens)	532
Reinforcement Tensile Strength [MPa] *(EN ISO 15630-1)*	Φ12 (4 specimens)	717
	Φ25 (4 specimens)	656
Reinforcement Elongation after Fracture *(EN ISO 15630-1)*	Φ12 (4 specimens)	21.5%
	Φ25 (4 specimens)	23.4%
Reinforcement Elongation at Maximum Force *(EN ISO 15630-1)*	Φ12 (4 specimens)	8.6%
	Φ25 (4 specimens)	14.4%

Section 3
Monitoring plan

To monitor the specimen's response during the tests, we extensively instrumented it with multiple sensors:

- We installed 18 displacement transducers (including one long-gauge cable sensor) at different locations in the building to monitor the vertical displacement of the removed columns, the lateral displacement (horizontal drift) of the two corner columns C1 and C9, and the separation between beams and slabs and between slab (hollow core) units (**S-Fig. 3**).

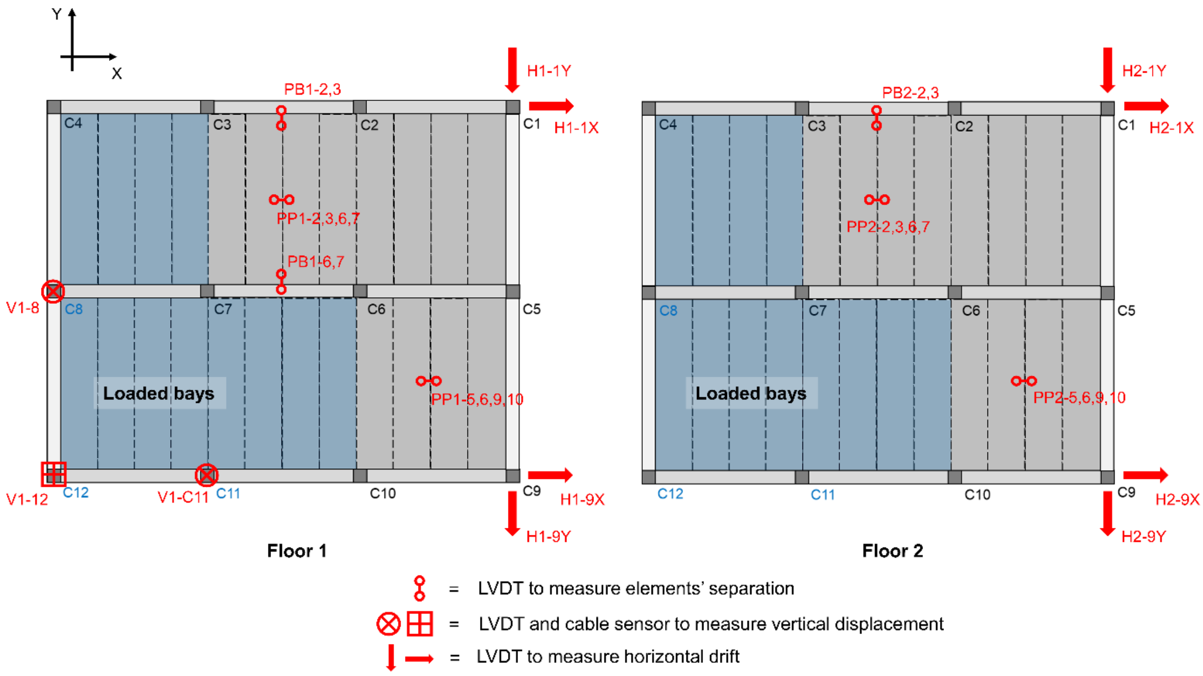

Supplementary Figure 3. The layout of displacement transducers and their identification and nomenclature.

- We installed 57 strain gauges at reinforcing bars inside the columns, slabs, and beams. However, some of them were producing unreliable measurements, so only the selected ones are presented and discussed in this report.
  - Column longitudinal bars to monitor the redistribution of gravity loads (**S-Fig. 4**). All strain gauges were placed at 40 cm from the bottom end of the columns.
  - Steel columns (2 strain gauges) to monitor the removal process and estimate the removal time (**S-Fig. 4**);
  - Dowel bars connecting the beam to the corbel to monitor the role and activation of the dowel bars in maintaining the integrity of the beam-column connection (**S-Fig. 5**);

- Bars connecting beams and planks and between planks to monitor the contribution of floor slabs in load-carrying mechanisms (**S-Fig. 6**);
- Continuity bars in the beams at the collapsed part, at the border, and at the upright part farther from the failure border to measure the degree of damage caused by the partial collapse (**S-Fig. 7**);

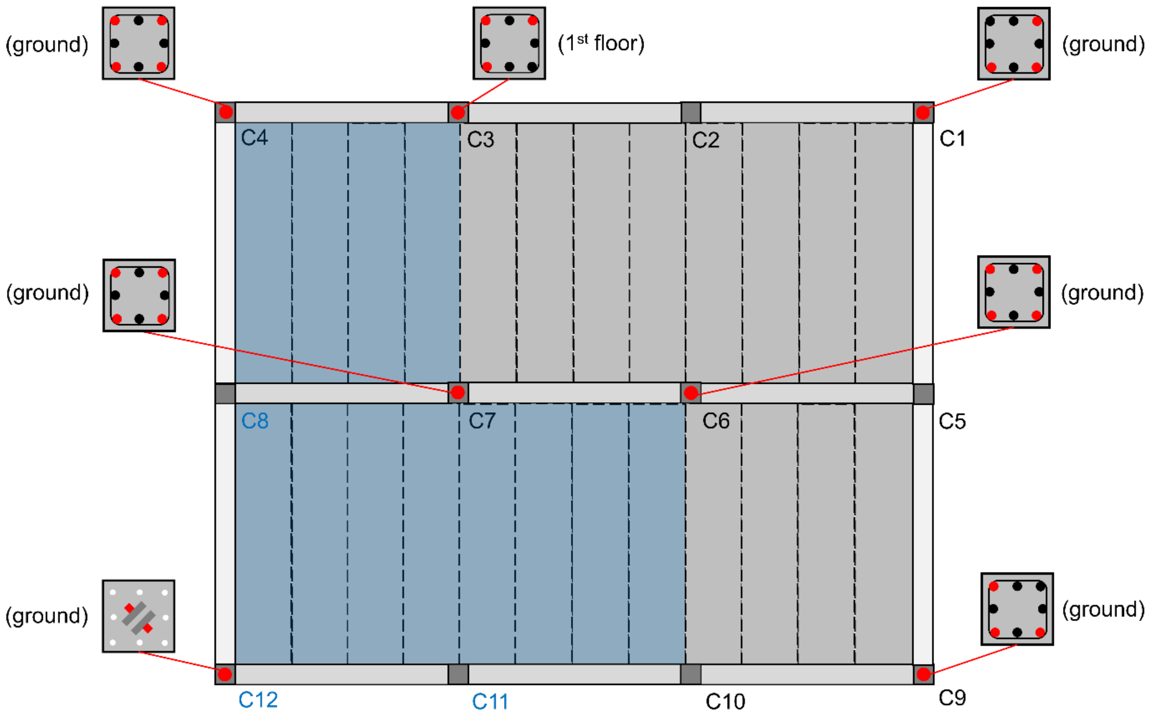

Supplementary Figure 4. The layout of columns' strain gauges (red dots indicate bars with strain gauges).

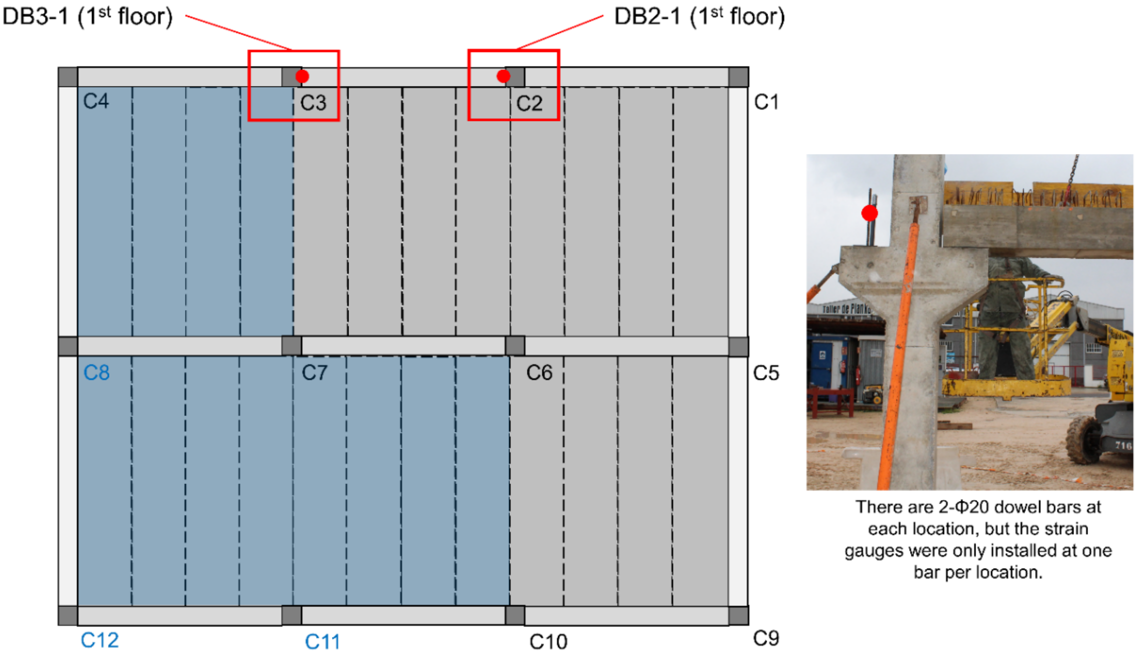

Supplementary Figure 5. The layout of dowel bars' strain gauges.

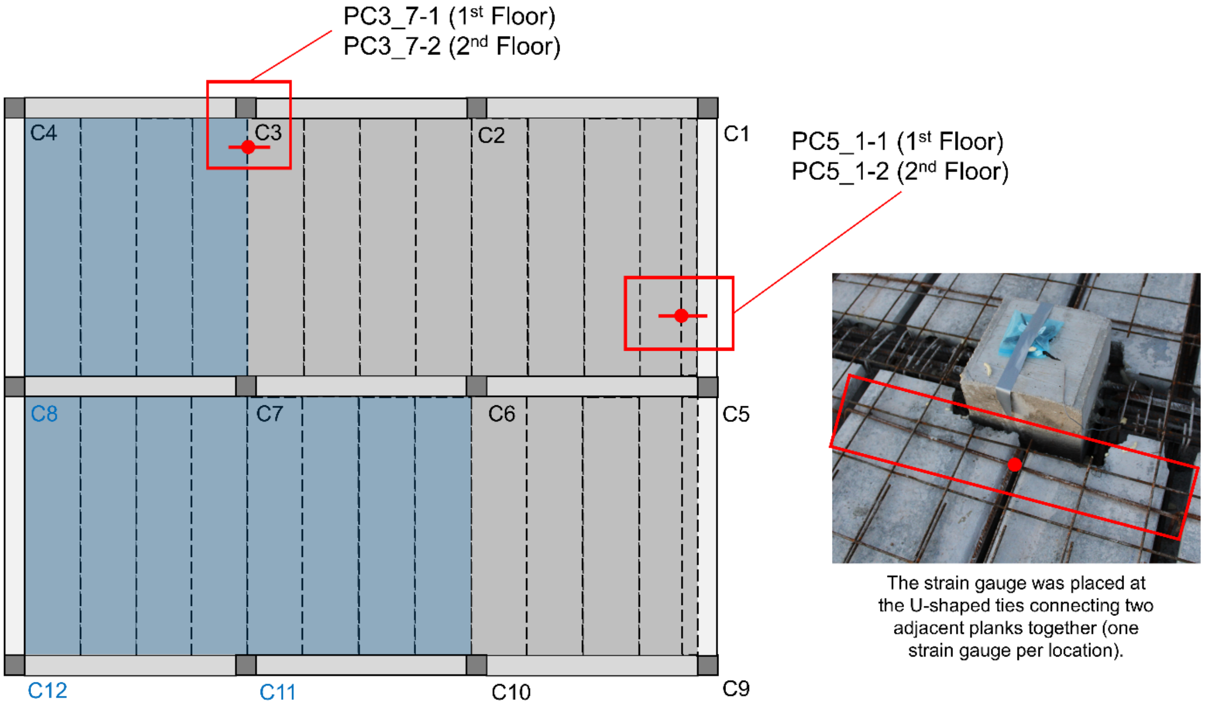

Supplementary Figure 6. The layout of U-shaped ties' strain gauges (connecting two adjacent plank units).

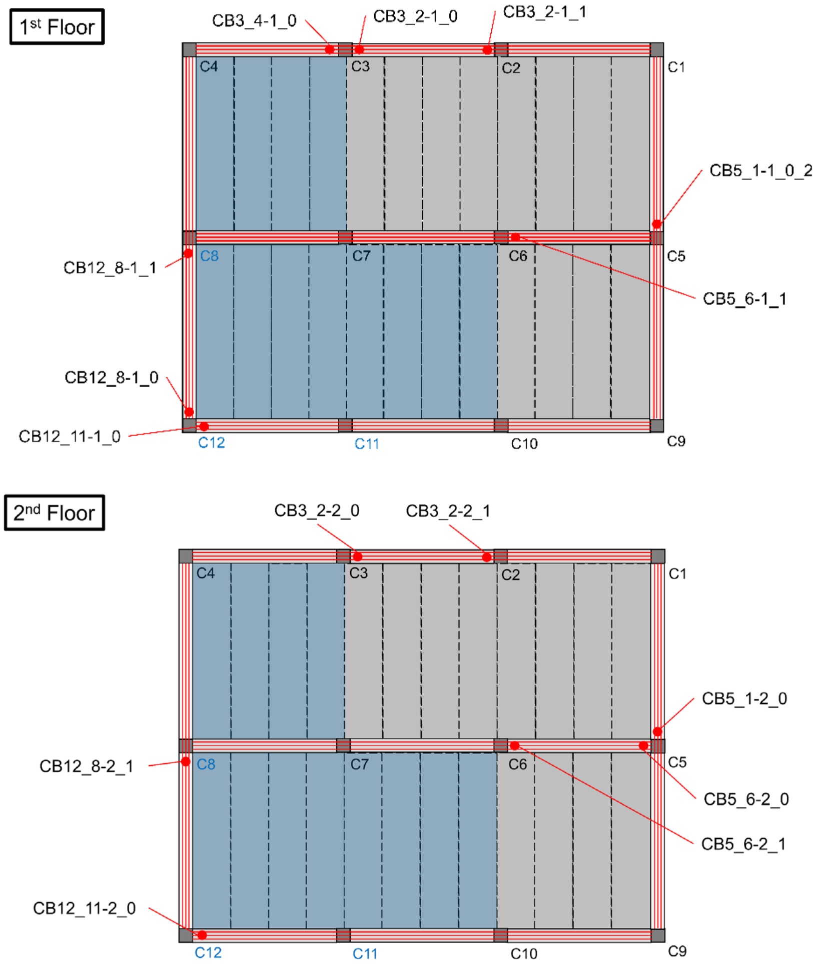

Supplementary Figure 7. The layout of beam continuity bars' strain gauges.

- We installed 5 accelerometers to measure the vertical (free fall) vibration of the expected failure bays, vertical vibration of the upright parts before and after the partial collapse, and horizontal accelerations in both principal axes of the building (**S-Fig. 8**).

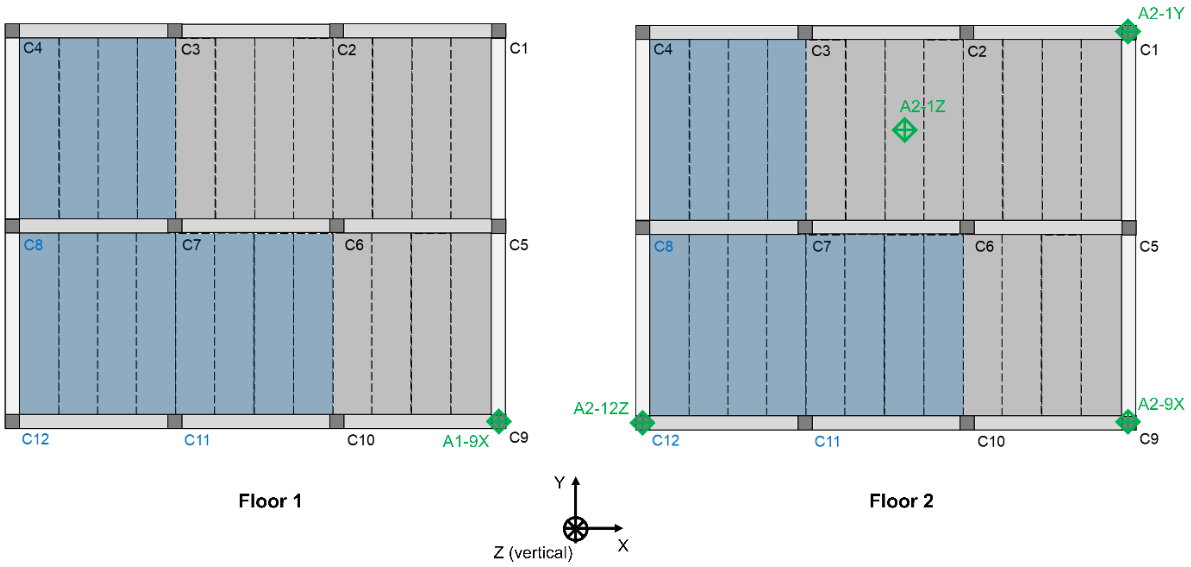

Supplementary Figure 8. The layout of accelerometers (x, y, z indicates the direction of the measured accelerations).

- We placed 5 high-resolution cameras to document the evolution of damages (visual) at different angles of the building during the test.
- We used 2 drones to capture the aerial view of the partial collapse process (refer to Supplementary Video 6) and to perform a post-collapse inspection (refer to Supplementary Video 8).

Section 4
Structural response in Phase 1 of testing

Before the column removal process, the building was loaded with more than 8000 sandbags (**S-Fig. 9a**), representing a uniformly distributed load (UDL) of 11.8 kN/m^2. We only applied these loads in the three bays that are directly affected by the column removals (see the blue-shaded regions in **S-Fig. 9b**) as our preliminary simulations revealed that this partial loading configuration resulted in a more critical condition where a higher magnitude of the unbalanced moment is introduced to the surrounding columns. Phase 1 of testing involves gradually removing columns C8 and C11, representing a small initial failure that may occur during abnormal events. These two specific columns were selected as they are the two closest edge columns to the corner bay of the building that, in real building, is the most vulnerable against external disturbances like vehicle impacts or explosions. For this purpose, we constructed two hanging concrete columns supported only on a vertical hydraulic jack (**S-Fig. 1b**). During the test, the pressure in the vertical jacks was gradually released from a safe distance to remove the vertical reaction supporting the column. The current building codes ensure that buildings can survive such an initial failure by relying on developing alternative load paths (ALPs) that are critical in redistributing the loads from the failed components to the rest of the system. Phase 1 explicitly aims to evaluate the building specimen's ability to withstand such an initial failure by forming the ALPs, hence preventing the collapse initiation. To monitor the behaviour of the structure during this process, measurements were extracted using sensors with a sampling rate of 200 Hz.

1. Vertical displacements at columns C8 and C11

The building was stabilised at about 250 seconds from the start of the loading phase (**S-Fig. 9a**). Two displacement transducers were attached at the top end of the ground floor columns C8 and C11 to monitor the vertical displacements during the quasi-static removal (**S-Fig. 9b**). We decided to remove the sensors to protect them from damage during Phase 2 of the test (**Section 5**). The results expressed in time (until 250 s) are shown in **S-Fig. 9c** below. The peak vertical displacements were approximately 65 mm and 60 mm in columns C11 and C8, respectively. The two displacements were relatively similar but not equal, as the span length and the behaviour of the hollow-core slabs are not symmetrical on both axes. These displacements induced an estimated chord rotation of about 0.01 – 0.015 radians (less than 1°) to the beams connected to these removed columns. This level of rotation can be considered very small when compared to the theoretical rotation limit under the catenary state that corresponds to the fracture of the bars (typically assumed to be around 0.2 radians or about 11°) yet sufficiently high to cause severe flexural concrete cracking and local yielding of the rebars around the region with the highest bending moments (positive bending moment at the location where the columns are removed). Normalising the vertical displacements with the beam's overall height, we obtain a value of

approximately 0.1, much lower than the displacements required to activate the catenary state (vertical displacement/beam height ≅ 1.0). This vertical displacement/beam height ratio and the chord rotation are the first indicators that the beams are still within the flexural state and have not entered the catenary state.

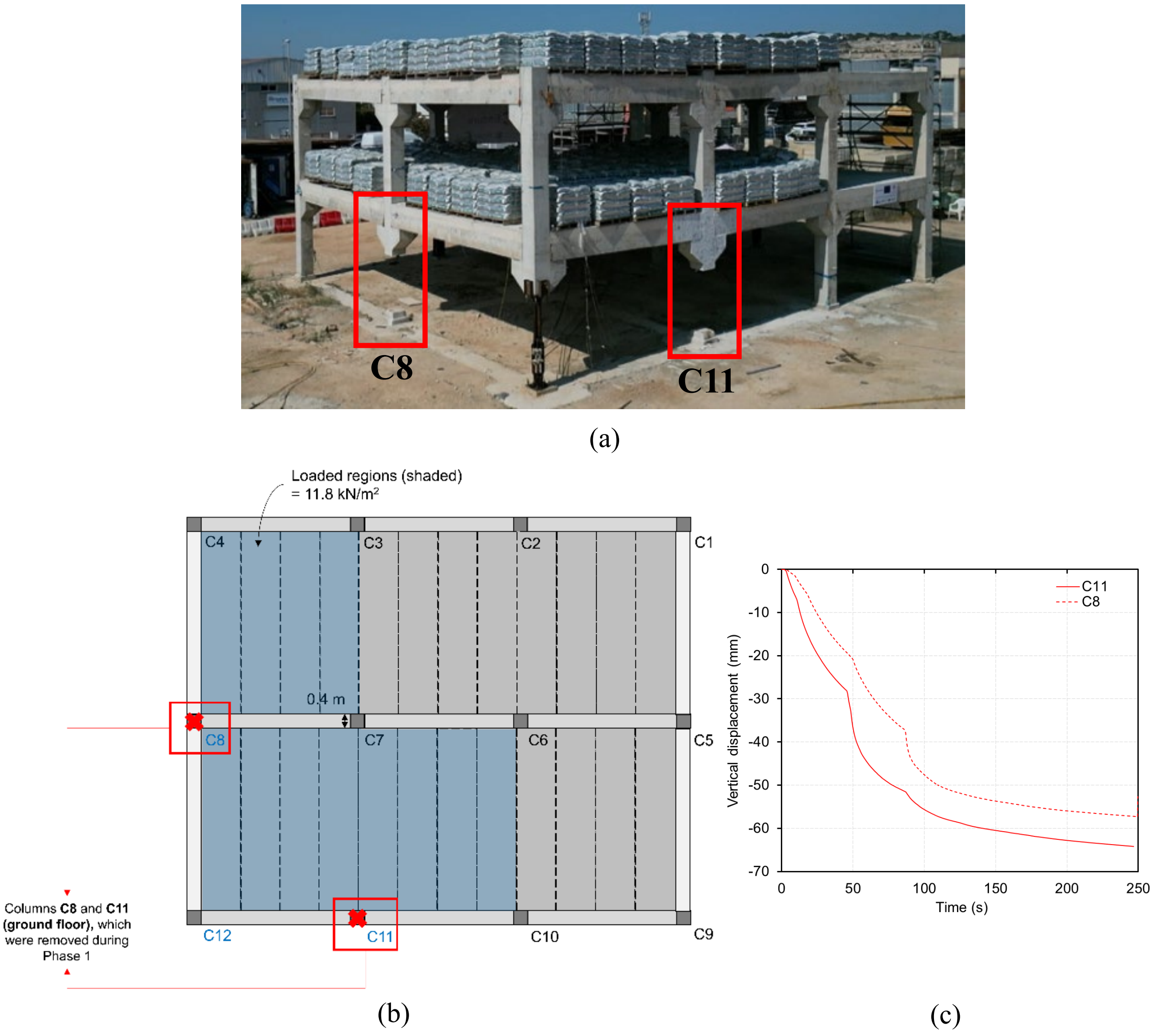

Supplementary Figure 9. (a) Deformed shape of the building specimen; (b) Plan view of the building specimen indicating the loaded regions and the removed columns; (c) Vertical displacements of columns C11 and C8 during Phase 1 (negative values indicate downward displacement).

2. Horizontal displacement (referring to building drift) at columns C1 and C9

The horizontal displacement at corner columns C1 and C9 at the first- and second-floor elevation was monitored (**S-Fig. 10a**). The displacement transducers were attached on one end to the building specimen and the other end to a fixed point outside the specimen (i.e., temporary steel structures located outside but in the vicinity of the building specimen – **S-Fig. 10b**). This was done to have a steady reference point. **S-Figs 10c-f** show the horizontal displacements at columns C1 and C9 on both floors.

The X and Y directions represent the axis parallel to the longer and shorter sides of the building, respectively.

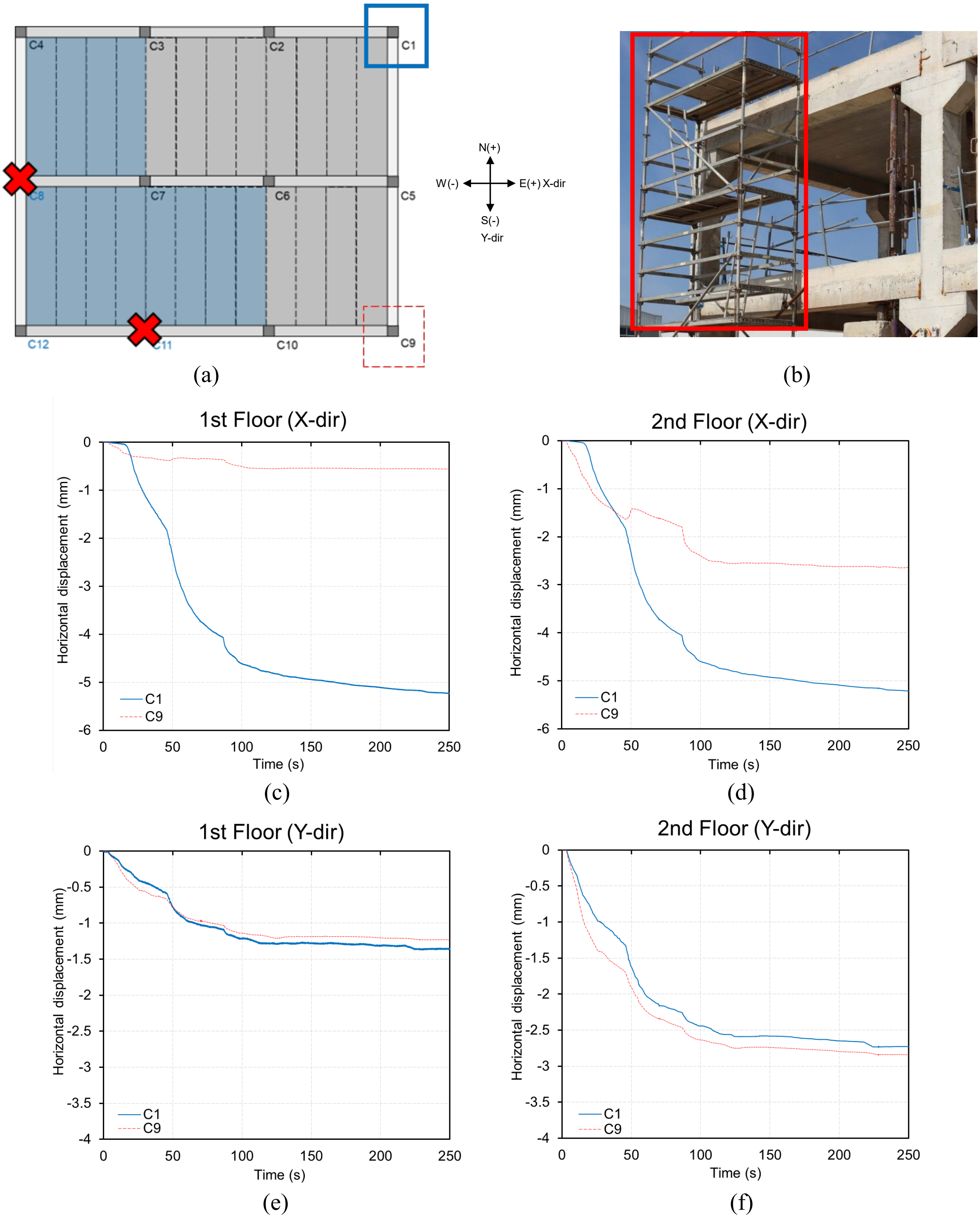

Supplementary Figure 10. (a) Monitored columns and the sign convention; (b) Temporary supports for attaching the sensors; (c) 1st Floor displacement in X-direction; (d) 2nd Floor displacement in X-direction; (e) 1st Floor displacement in Y-direction; (f) 2nd Floor displacement in Y-direction during Phase 1.

We argue that the horizontal displacement of the building is an important parameter to check as it may indicate whether significant pulling forces were generated due to the removal of columns C8 and C11, which may affect the stability of the building. In particular, this magnitude of horizontal displacement (building drift) will be compared between Phase 1 and 2 to evaluate the impact of small and large initial failures on the building system.

A general trend can be observed in **S-Figs 10c-f** that the second floor experienced more significant horizontal displacements in both directions than the first floor. The drift in the Y-direction indicates that columns C1 and C9 behaved almost identically as both columns were pulled toward the south direction. In contrast, the drift in the X-direction shows a twisting in-plane movement as column C1 experienced significantly larger displacements than C9. This is caused by the loading configuration and the location of the removed columns that are asymmetric in the plan. Normalising the magnitude of the inter-storey displacement to the floor-to-floor height, we determine a storey drift of less than 0.2%. According to ASCE 7-22 [8], the standard allowable drift limit for wind design serviceability is 0.17-0.25%, showing that the structural response even in accidental scenarios is appropriate for serviceability limit states. We concluded that the building specimen performed well (within this expected range of serviceability limit) even when subjected to two-column removal.

3. Horizontal displacement (referring to separation) of the plank-to-plank and plank-to-beam components

When a building is subjected to a loss of load-bearing elements during extreme events, its survivability depends on the ability of the system to redistribute the loads from the failed components to their surroundings. At this state, connectivity between structural components is a key aspect to ensure the integrity of the building. To monitor this phenomenon, we installed displacement transducers at two different types of connectivity, between the hollow core slab and the beam (referred to as plank-to-beam/PB) and between two adjacent hollow core plank units (referred to as plank-to-plank/PP). **S-Fig. 11** shows the photographs where these two components are measured in the building specimen.

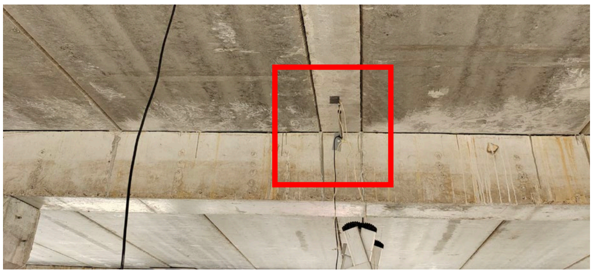

(a)

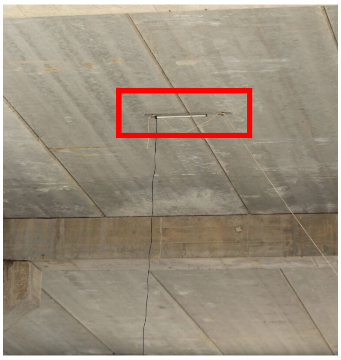

(b)

Supplementary Figure 11. (a) A transducer measuring the plank-to-beam (PB) separation; and (b) the plank-to-plank (PP) separation.

S-Fig. 12 shows the horizontal separation of the plank-to-plank, whereas **S-Fig. 13** shows the plank-to-beam components.

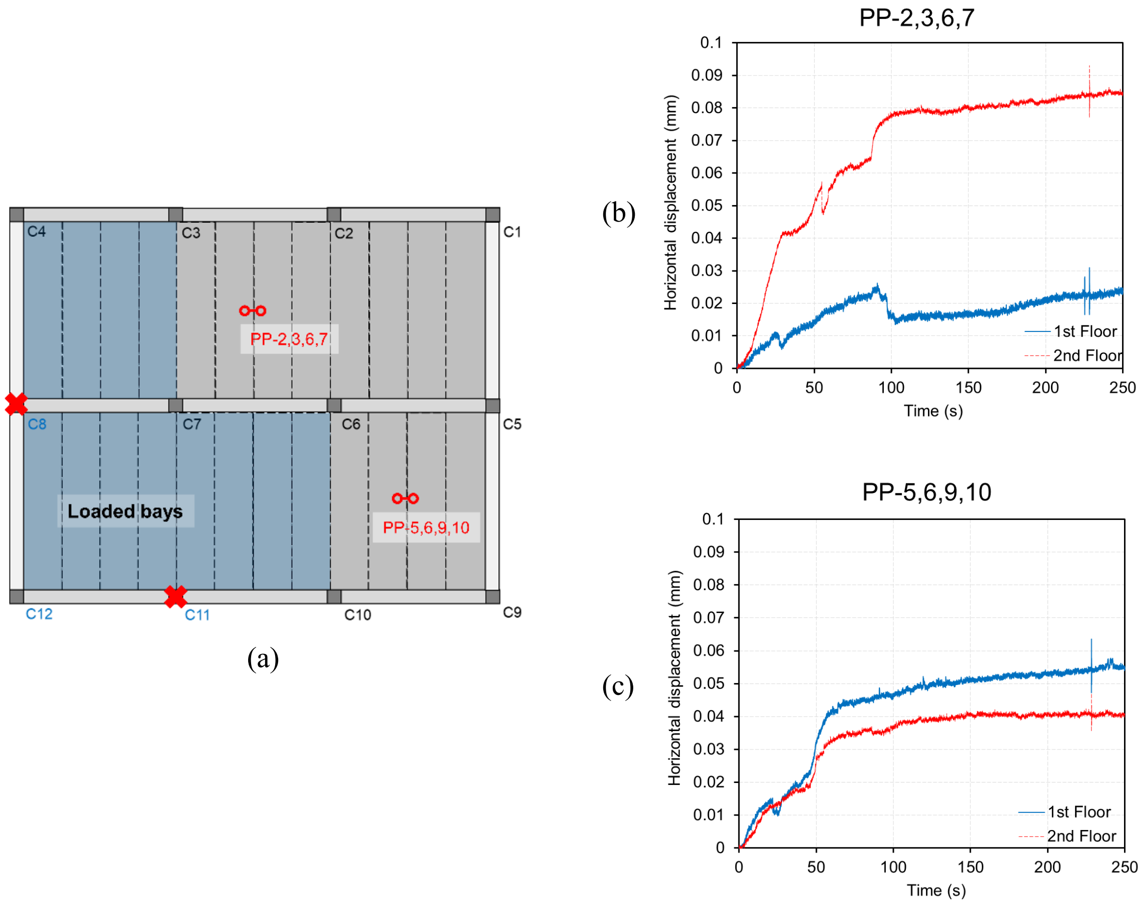

Supplementary Figure 12. (a) The layout of the displacement transducers to measure the plank-to-plank (PP) separation; (b) PP separation measured at the bay between columns C2, C3, C6, and C7; (c) PP separation measured at the bay between columns C5, C6, C9, and C10 during Phase 1 (positive values indicated separation/opening).

Generally, relatively small separations/openings (< 0.1 mm) were observed in all locations, yet they were not negligible. These limited separations indicated that the continuity between plank-to-plank and beam-to-plank (through ties) was well-functioning to restrain the horizontal movement, maintaining the system's integrity in providing an alternative load path. These measurements justify that the floor system if appropriately connected to the frame, can significantly contribute to the development of the alternative load paths, which help to redistribute the loads previously supported by the failed columns C8 and C11 to the surrounding components.

This observation is also later shown in agreement with the strain gauge measurement, revealing a significant increase in strain of the connecting bars between two adjacent plank units. Comparing the

two locations, the measured horizontal separation at PB-6,7 is between three to four times higher than PB-2,3, as the former is closer to the areas affected by the column removals.

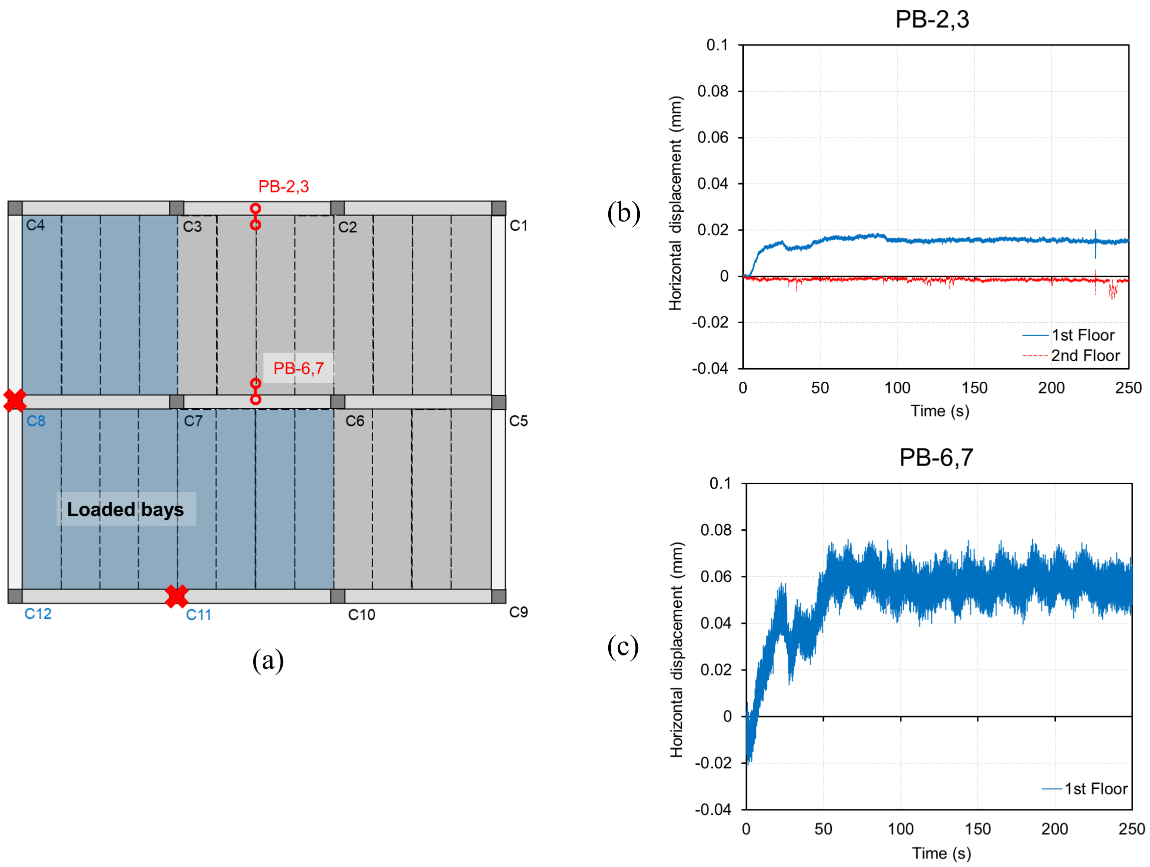

Supplementary Figure 13. (a) The layout of the displacement transducers to measure the plank-to-beam (PB) separation; (b) PB separation measured at the axis between columns C2 and C3; (c) PB separation measured at the axis between columns C6 and C7 during Phase 1 (positive values indicated separation/opening).

4. Strain increase of the columns' reinforcement bars

S-Fig. 14 summarises the average axial strain of the columns located on the ground floor during Phase 1. The columns are grouped as follows: 1) columns located closer to the regions affected by the column removals (C4, C7, and C12); 2) columns located farther away from the affected regions (C1, C6, and C9). Post-processing of the strain was done by performing a "moving average" for every 20 data points to limit measurements' noise. For columns with a complete set of four strain gauges installed at the four corner bars, the axial strain is calculated as the average of the four gauges. When only three strain gauges are available, the axial strain is computed as the average of the two bars at the two opposed diagonal corners. All three monitored columns closer to the affected region (C4, C7, and C12) were subjected to negative strains, indicating increased compressive forces. The highest increase was observed in the internal concrete column C7 (-120 με), followed by the corner steel column C12 (-60 με), and the lowest is corner concrete column C4 (-30 με). This increase provides us with an indication of where the loads are redistributed. The other three monitored columns (C1, C6, and C9) were subjected to lower changes

in axial strain (approximately ± 20 με), some of which were positive. This relaxation is caused by the unbalanced condition where the affected bays moved downward due to the column's removals, causing the regions at the opposite corner to move upward (e.g. column C1).

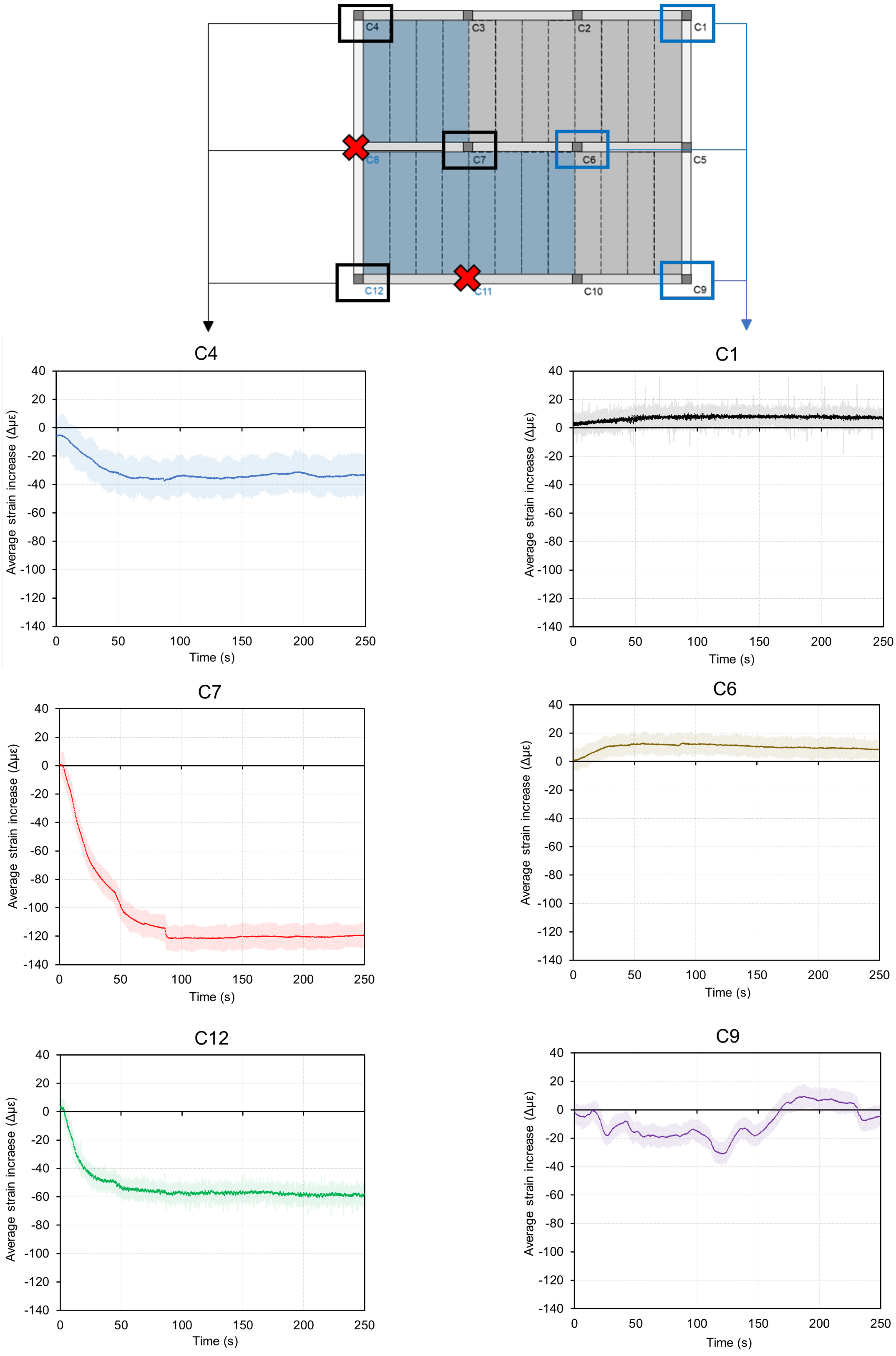

Supplementary Figure 14. Average (axial) strain increase of columns' bars (ground floor) during Phase 1 (positive values indicate tensile strains).

5. Strain increase of the dowel bars (connecting the beams and corbels)

In addition to reinforcement bars in the columns, strain gauges were also installed in the dowel bars, connecting the bottom part of the beams (precast element) to corbels and measuring the possible elongation of the bar. These dowel bars were designed to resist the horizontal compression forces, allowing the negative moment resistance (hogging moments) of the beam to be partially developed at the support location (beam-column connection). **S-Fig. 15a** shows the location of the monitored dowel bars, and **S-Fig. 15b** shows the measured strains.

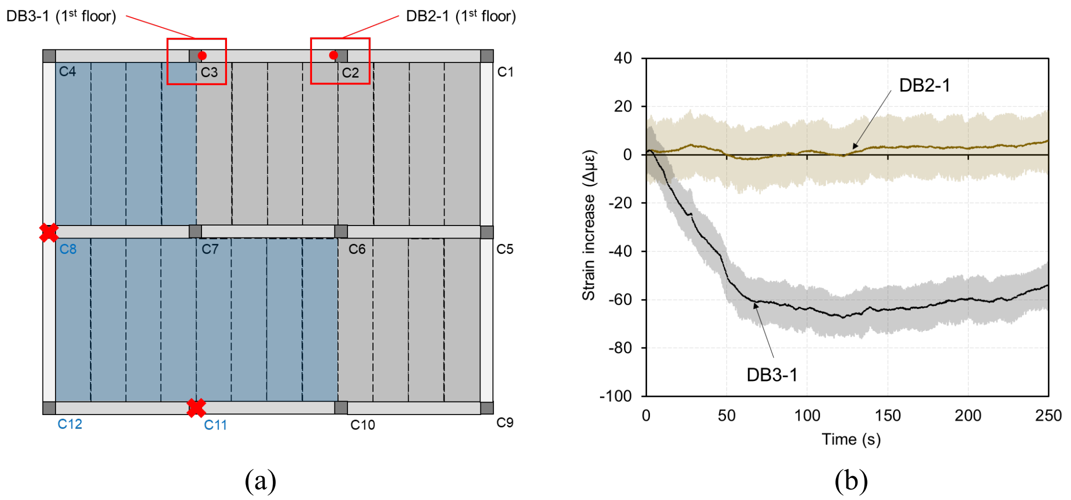

Supplementary Figure 15. (a) Location of the monitored dowel bars; (b) Strain increase in dowel bars during Phase 1 (positive values indicate tensile strains).

A strain increase was observed in dowel bars DB3-1, indicating that the flexural capacity of the beam is mobilised to provide the alternative load path (ALP) after the removals of columns C8 and C11. In contrast, dowel bars DB2-1 remained unchanged as this beam was located farther from the affected region and not necessarily required to accommodate the ALP.

6. Strain increase of the U-shaped ties (connecting two adjacent plank units)

The U-shaped ties connecting two adjacent plank units were monitored using strain gauges. This measurement would allow us to quantify the contribution of the floor slabs in forming the alternative load path for redistributing the excess loads after the column removal process. **S-Fig. 16a** shows the location of the monitored ties, and **S-Fig. 16b** shows the measured strains. A significant strain increase (tensile) was observed in the bars connecting the two planks between columns C3 and C7, as these planks were located close to the affected bays. In particular, the rebars on the second-floor slabs were strained more (almost double) than the first-floor ties. This indicates that the continuity between floor units provided by these ties was necessary to maintain the integrity of the floor system. These ties were also critical to limiting the crack (separation) between adjacent plank units. Otherwise, the planks would

behave as an individual unit, not contributing to the ALP in their perpendicular direction. On the other hand, no strain increase was observed in the bars between columns C5 and C1 as they are located farthest from the affected bays.

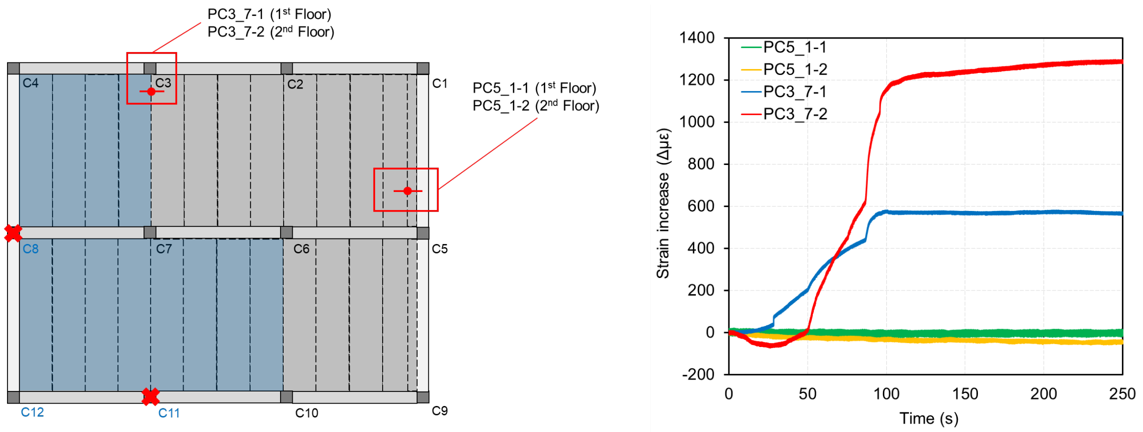

(a) (b)

Supplementary Figure 16. (a) Location of the monitored U-shaped ties connecting two adjacent plank units; (b) Strain increase in the U-shaped ties during Phase 1 (positive values indicate tensile strains).

7. Strain increase of the continuity bars (continuous top beam bars)

To monitor the contribution of the beams in providing the alternative load path, the top continuity bars were monitored with strain gauges (**S-Figs. 17a-b**). The strain increase of the continuity bars in these monitored locations is shown in **S-Figs. 17c-d**. The increase of strain in these bars indicated the activation of the alternative load path (ALP) that makes the load redistribution possible. Observing the change of rebar strain in various beam locations revealed that only beams connected directly to the removed columns were highly strained; refer to CB12_8 and CB12_11 for beams connecting C12-C8 and C12-C11, respectively. The continuity bars were also activated in the rest of the beams but at the lowest strain level (subtle increase). The trend generally indicates that the farther the beam is located from the affected bays, the lower the strain (the same trend was observed for the columns' axial strains). In two particular locations, CB12_11-1_0 (first floor) and CB12_11-2_0 (second floor), it was observed that the increase in rebar strain surpasses the yield strain. Due to the asymmetry of the floor system (the hollow core slabs behave as a one-way slab system), the continuity bars installed within the primary beam (connecting columns C11 and C12) were more highly strained when compared to the perpendicular (secondary) beam connecting columns C8 and C12. Comparing the increase of rebar strain in the first- and second-floor beams indicates that a more uniform increase can be observed in the first-floor beams, whereas, for the second-floor beams, only one location was highly strained (the beam connecting columns C11 and C12). This indicates that the damage is more concentrated on the second floor. Nevertheless, the strain measurements suggest that all the bars are still much below the rupture strain (approximately 0.15ε).

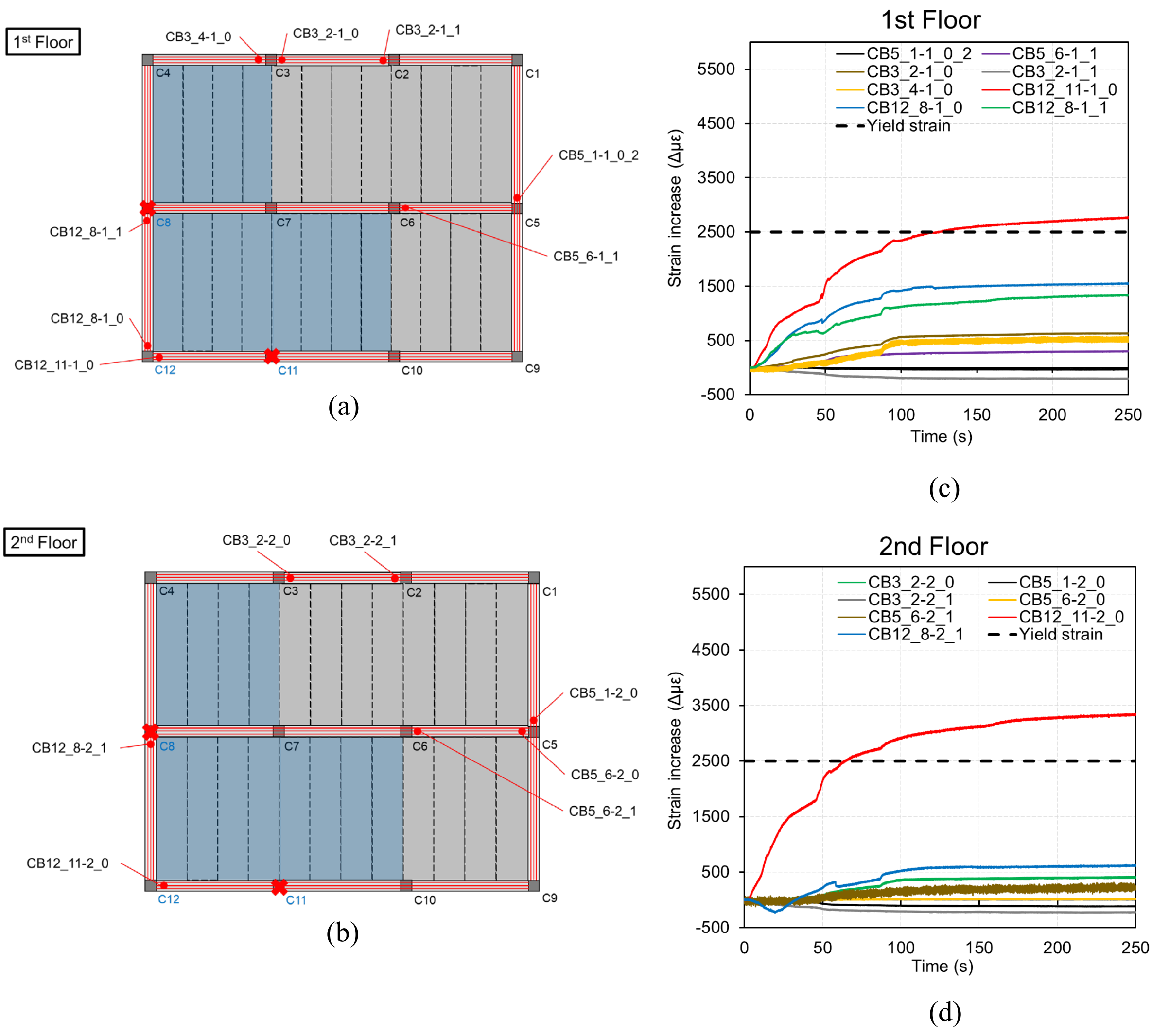

Supplementary Figure 17. Layout of the monitored bars in the (a) 1st Floor; (2) 2nd Floor; Strain increase in the continuity bars in the (c) 1st Floor; (d) 2nd Floor during Phase 1.

Section 5

Structural response in Phase 2 of testing

After performing the column removals in Phase 1, we had a 10-minute pause before proceeding with Phase 2. This was done to ensure the building had sufficient time to stabilise itself while allowing the sensors to monitor all the phenomena occurring during this transition. After this delay, we performed the dynamic column removal by destabilising the three-hinged steel column (C12). We pulled the middle hinge horizontally using a forklift and a cable from outside the building. Immediately, a partial collapse occurred only at the loaded regions, while the rest of the system remained standing after experiencing some vibrations (Supplementary Video 6). The whole collapsing process lasts for less than 10 seconds. For this reason, all the measurements shown here are already filtered only to show this selected timeframe. The sampling rate of the sensors was the same as Phase 1, which is 200 Hz, except for the accelerometer, which is 1000 Hz.

1. Dynamic column removal and the damaged state of the building specimen at Phase 2

To monitor the vertical downward movement of column C12, we installed a displacement transducer cable sensor connected at one end to the concrete corbel just above the steel column and the other to the zero elevation at the base of the ground floor. In addition, two strain gauges were attached to the steel columns to monitor the removal time.

S-Figs. 18a-b shows the damaged state of the building specimen after Phase 2. The dynamic removal of column C12 induced partial collapse at the loaded bays, creating a separation (border) in the two principal building axes between the upright and collapsed parts. The separation occurs in two primary forms. First is the separation between beams and columns where the top continuity bars broke at the column face (**S-Fig. 18c**), damaging the corbels. Second, the top bars of the hollow core slabs (rebar mesh) experienced bond failure where the bars slipped from the concrete topping at the top part of the slab (**S-Fig. 18d**).

S-Fig. 18e shows the downward movement of column C12 measured by the displacement cable sensor. The strain measurement at column C12 revealed a removal time of 0.06 s. Column C12 fell to the ground with an average velocity of 1.7 m/s, where we lost the signal from the strain gauges at about 2.8s after the test commenced. The total load previously supported by C12 was estimated at 640 kN.

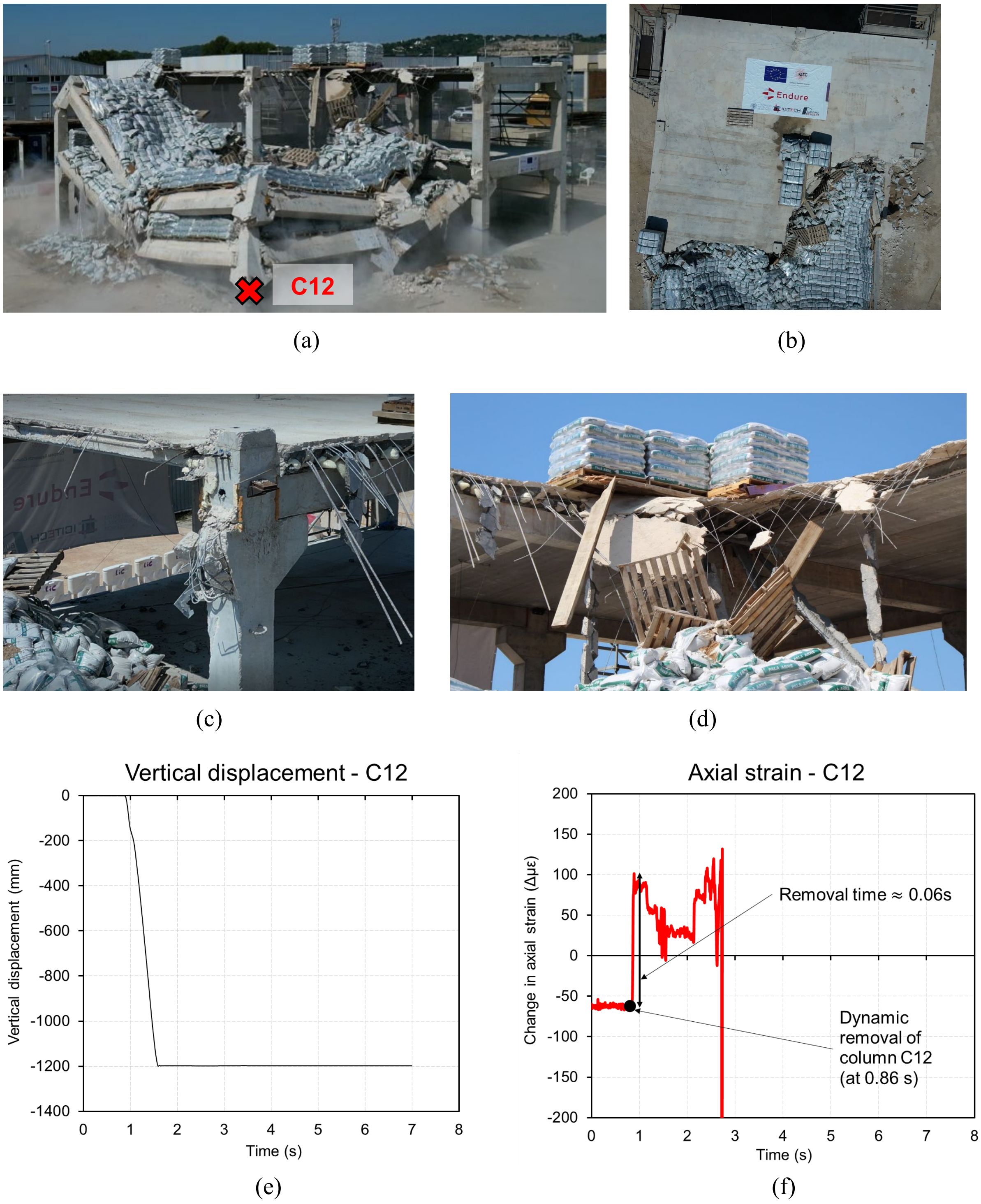

Supplementary Figure 18. (a) 3D-view and (b) Aerial view of the damaged state of the building specimen after Phase 2 of testing; (c) Separation at beam-column connections; (d) Bond failure of rebar mesh of the hollow core slabs; (e) Vertical displacement of column C12 measured by the displacement cable sensor; (f) Change in axial strain of column C12 measured by strain gauges.

2. Horizontal displacement (referring to building drift) at columns C1 and C9

The horizontal displacements at corner columns C1 and C9 on the first and second floors were monitored using eight displacement transducers. Unfortunately, due to some unexpected external disturbance, the measurements obtained by transducers at column C1 were unreliable, so only measurements from C9 are shown here. The magnitude of the measured horizontal displacement provides an excellent indication of the horizontal pulling forces generated during the collapse process. **S-Fig. 19a** indicates the monitored location (column C9) and the sign convention to interpret the measurement. The horizontal displacements in the X- and Y-direction of C9 are shown in **S-Fig. 19b** and **c**, respectively.

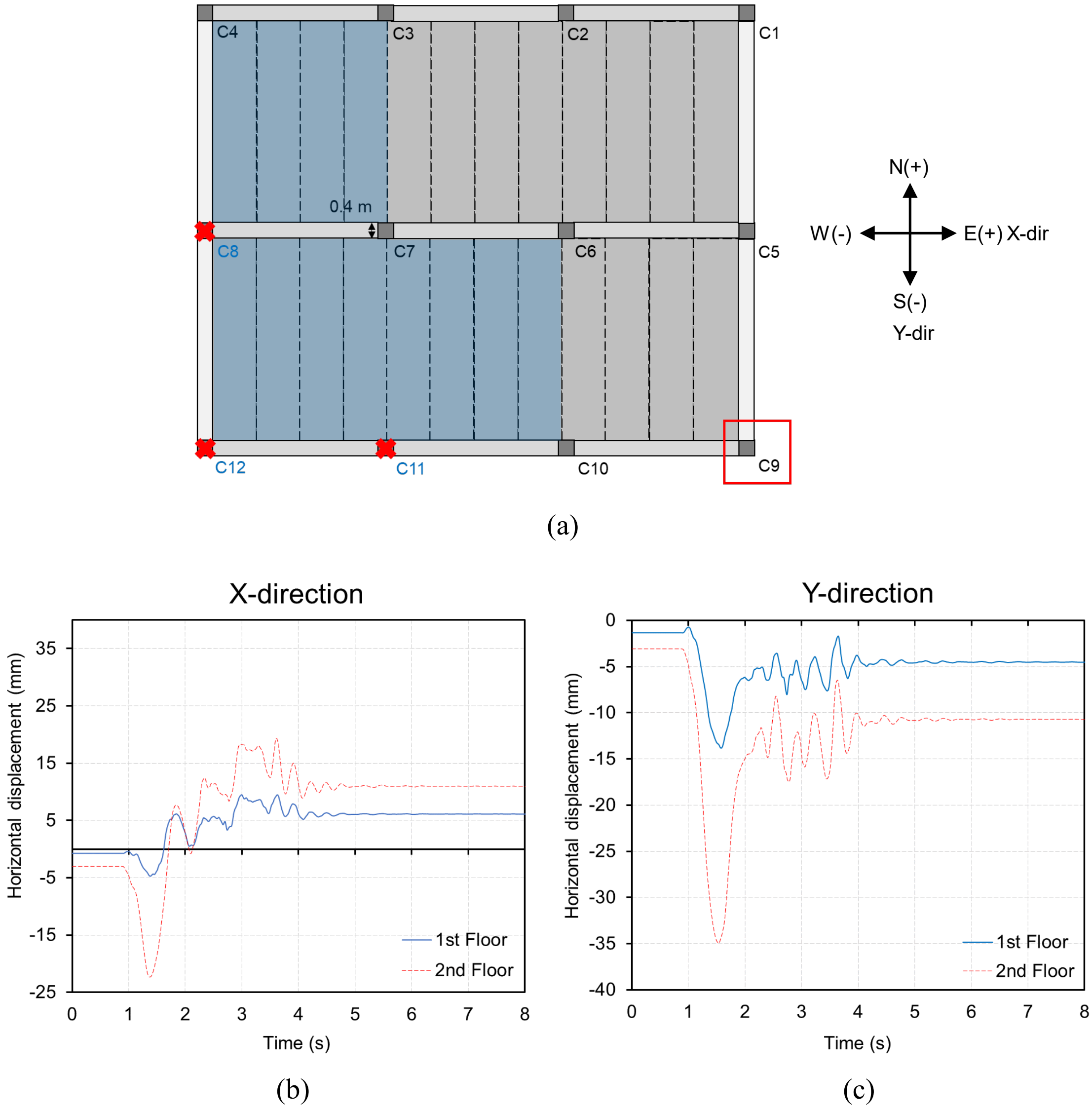

Supplementary Figure 19. (a) Monitored columns and the sign convention; (b) Horizontal displacement of C9 in the X-direction; (c) Horizontal displacement of C9 in the Y-direction during Phase 2.

After the separation, a significant vibration was reflected from the measured horizontal displacements of the building in both X and Y directions. As observed in Phase 1, the measured inter-storey drift was also higher on the first floor than on the ground floor in Phase 2. The peak roof drift was observed in

the Y direction with a value of 35 mm, corresponding to 0.7% drift (normalised by the building's height). To provide a context, a typical drift limit allowed for buildings located in earthquake-prone areas subjected to the maximum considered earthquakes (MCE_R) with a 2500-year return period, according to the American Society of Civil Engineers (ASCE), is between 1.0 – 2.0% [8], depending on the occupancy category and performance target (independent of the location). The measured peak drift from Phase 2 was relatively close to this range, which we can be considered as an extreme event. After reaching the peak values, the vibration decayed, but a residual (permanent) total horizontal displacement existed with a value of about 10 mm on the first floor in both directions. Comparing the pattern observed in the two directions, it is interesting to notice that a sudden sign reversal occurred (from negative to positive displacement) in the X direction, which indicates that the building suffered a significant whiplash effect.

3. Horizontal displacement (referring to separation) of the plank-to-plank and plank-to-beam components

S-Fig. 20 shows the horizontal separation of the plank-to-plank (PP), whereas **S-Fig. 21** shows the plank-to-beam components (PB) measured in Phase 2.

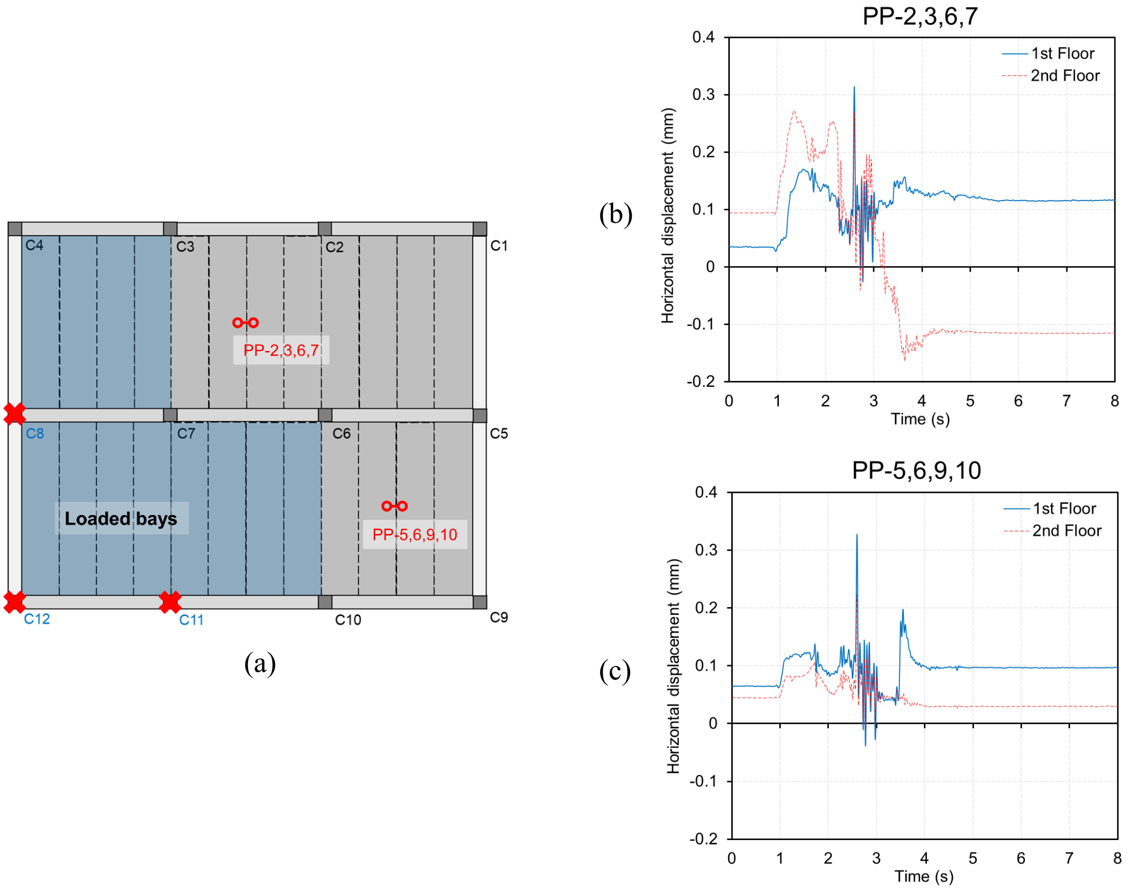

Supplementary Figure 20. (a) The layout of the displacement transducers to measure the plank-to-plank (PP) separation; (b) PP separation measured at the bay between columns C2, C3, C6, and C7; (c) PP separation measured at the bay between columns C5, C6, C9, and C10 during Phase 2 (positive values indicated separation/opening).

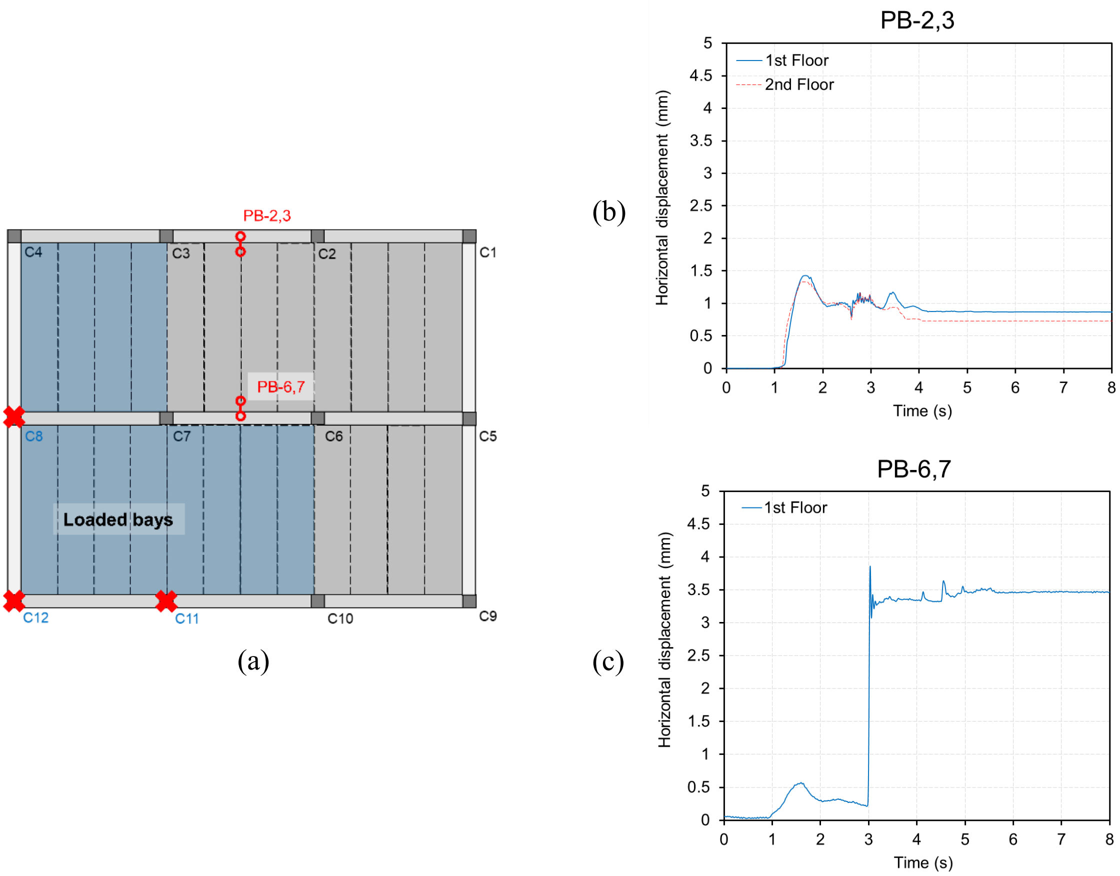

Supplementary Figure 21. (a) The layout of the displacement transducers to measure the plank-to-beam (PB) separation; (b) PB separation measured at the axis between columns C2 and C3; (c) PB separation measured at the axis between columns C6 and C7 during Phase 2 (positive values indicated separation/opening).

Comparing **S-Figs. 20** and **21**, we can observe that the magnitude of the horizontal separation was much higher in plank-to-beam compared to plank-to-plank connections (one order of magnitude higher). Mainly, PB-6,7, located closest to the failure border, showed the highest value (approximately 3.5 mm compared to 1.5 mm in PB-2,3). This horizontal separation was not recovered after the vibration decayed (resulting in significant residual/permanent deformations). Regarding the plank-to-plank separation, the magnitude of the separation is relatively comparable between the two locations and the two floors. In general, the measured horizontal displacements were in the 0.1 – 0.3 mm range. If compared to the magnitude of displacement during Phase 1, the peak value increases by 5-10 times in Phase 2, which indicates the severity of the partial collapse even for the upright part of the structure.

4. Strain increase of the columns' reinforcement bars

S-Fig. 22 summarises the average axial strain of the columns located on the ground floor, except for column C3, which is monitored at the first-floor level.

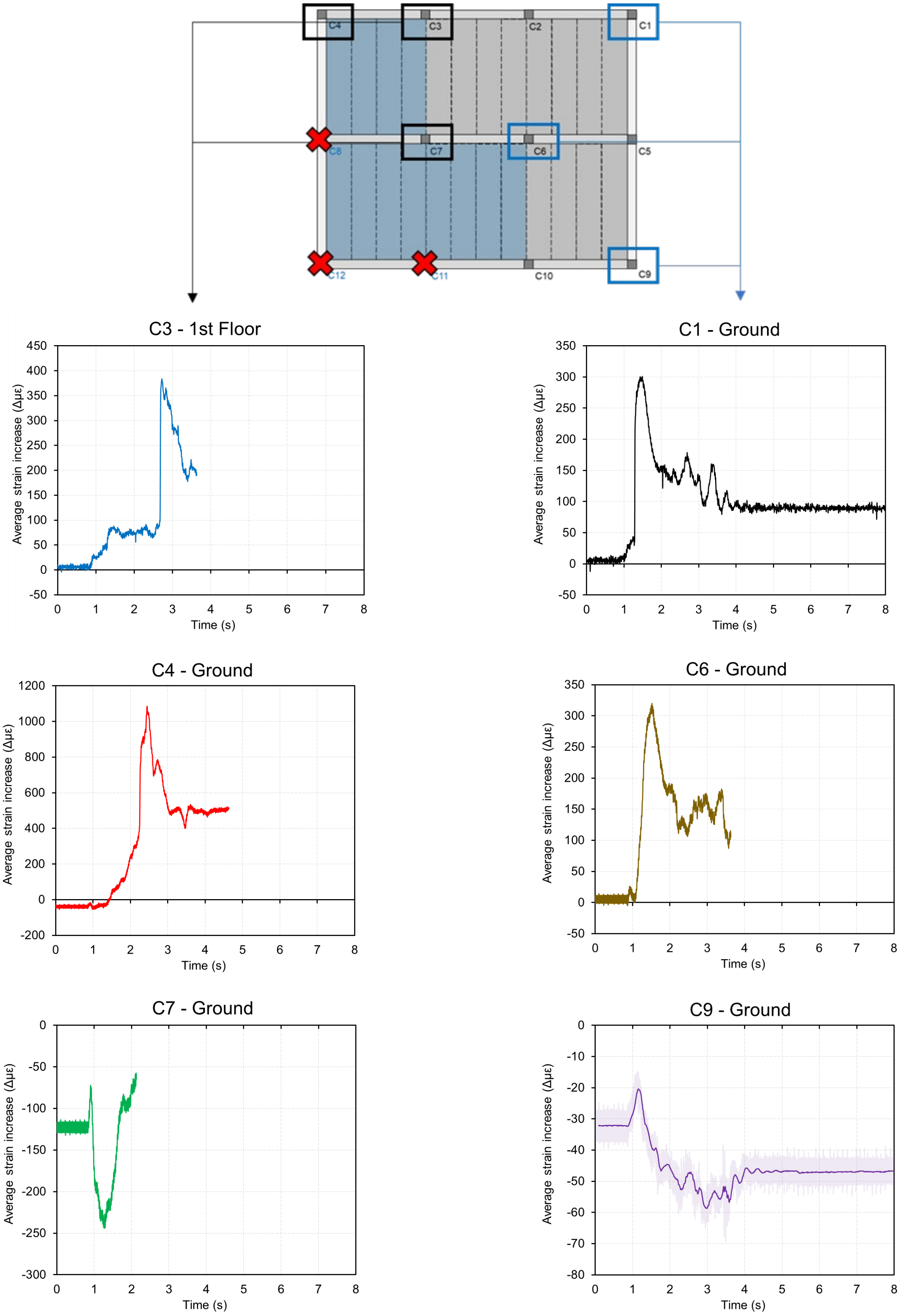

Supplementary Figure 22. **Average (axial) strain increase of columns' bars during Phase 2 (positive values indicate tensile strains).**

The columns are grouped as follows: 1) columns located closer to the regions affected by the column removals (C4, C3, and C7); 2) columns located farther away from the affected regions (C1, C6, and C9). It must be noted that we lost signal in some of the strain gauges before the 8 s; hence, some of the plots in **S-Fig. 22** are incomplete. Post-processing of the strain for C9 was done by performing a "moving average" for every 20 data points to limit the noise of the measurements (no post-processing is required for the other columns). For columns with a complete set of four strain gauges installed at the four corner bars, the axial strain is calculated as the average of the four gauges. In contrast, when only three strain gauges are available, the axial strain is calculated as the average of the two bars at the two opposed diagonal corners as representative of the average sectional axial strain.

Of these six columns, column C7 were the one that was highly stressed in compression just after the removal of column C12. This indicates that column C7 behaved as a pivot point that was most compressed when the three loaded bays fell to the ground and pushed up all columns behind C7 (i.e., the seesaw effect). This explains why C1 and C6 were subjected to positive (tensile) strain in **S-Fig. 22**. After the immediate strain jump in C7, the strain decayed after the peak in -250 $\mu\varepsilon$, indicating an unloading behaviour due to the separation of the slabs and beams in the three loaded bays (the collapsing parts). Simultaneously, columns C1, C3, C4, and C6 were subjected to a relaxation of compressive strain. Some even experienced tensile net strain due to the unbalanced moment and horizontal pulling forces generated during the collapse.

5. Strain increase of the dowel bars

S-Fig. 23 shows the strain increase of the dowel bars in the locations shown in **S-Fig. 23a**. The measurements suggest that both monitored dowel bars were activated during the partial collapse process: one experienced increased tensile strain, and the other decreased (relaxed). The peak strain ranges between 300 – 400 $\mu\varepsilon$. Comparing this magnitude of strain increase to Phase 1, we observe 5-20 times increase in strain, indicating that the dowel bars were highly strained during the collapse process in Phase 2. We can also observe the residual strains at the post-peak response, suggesting permanent/plastic damage occurs.

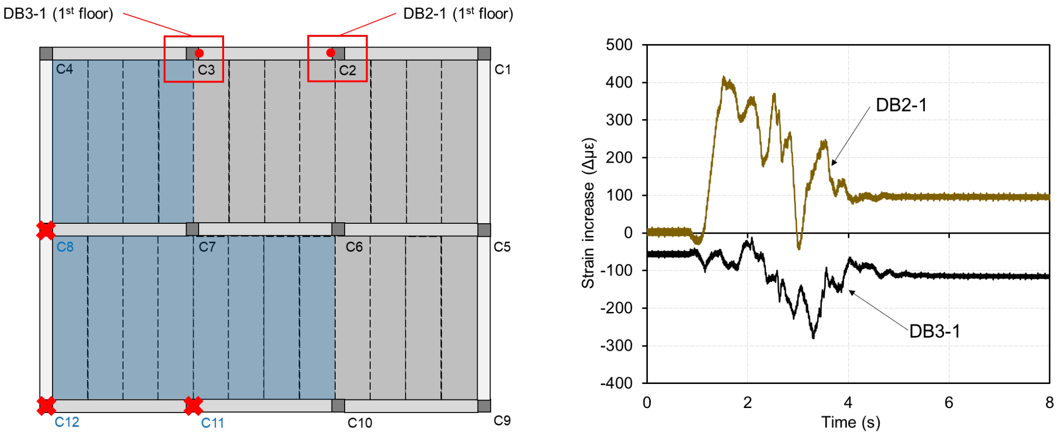

(a) (b)

Supplementary Figure 23. (a) Location of the monitored dowel bars; (b) Strain increase in dowel bars during Phase 2 (positive values indicate tensile strains).

6. Strain increase of the U-shaped ties

S-Fig. 24a indicates the location where strain gauges were placed to monitor the increase in strain of the U-shaped ties connecting two adjacent plank units, and **S-Fig. 24b** provides the measurement.

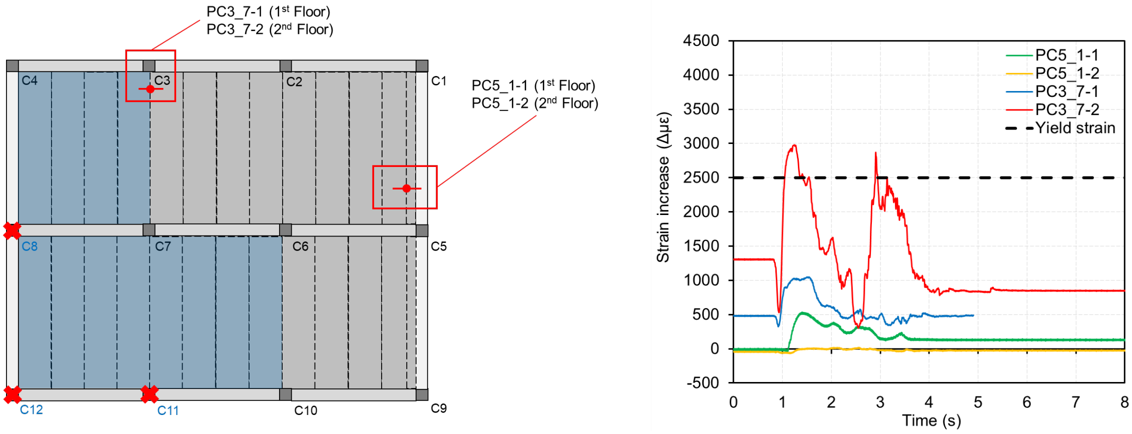

(a) (b)

Supplementary Figure 24. (a) Location of the monitored U-shaped ties connecting two adjacent plank units; (b) Strain increase in the U-shaped ties during Phase 2 (positive values indicate tensile strains).

Regarding the connecting ties between plank units, the most significant increase in strain was observed in the bays close to the failure borders: PC3_7-1 and PC3_7-2. In particular, the increase in strain of the second-floor ties exceeded the expected yield strain. This observation is consistent with the sudden increase in the plank-to-plank separation shown in **S-Fig. 20b** (i.e., we can relate the significant jump of crack/separation between the two planks after the yielding of the ties). On the other hand, the other

two monitored locations located farther away from the failure border (PC5_1-1 and PC5_1-2) were less activated.

7. Strain increase of the continuity bars

S-Fig. 25 shows the strain increase of the continuity bars located close to the failure border. In contrast, **S-Fig. 26** shows the rest of the measured continuity bars that are farther (less affected) from the failure border.

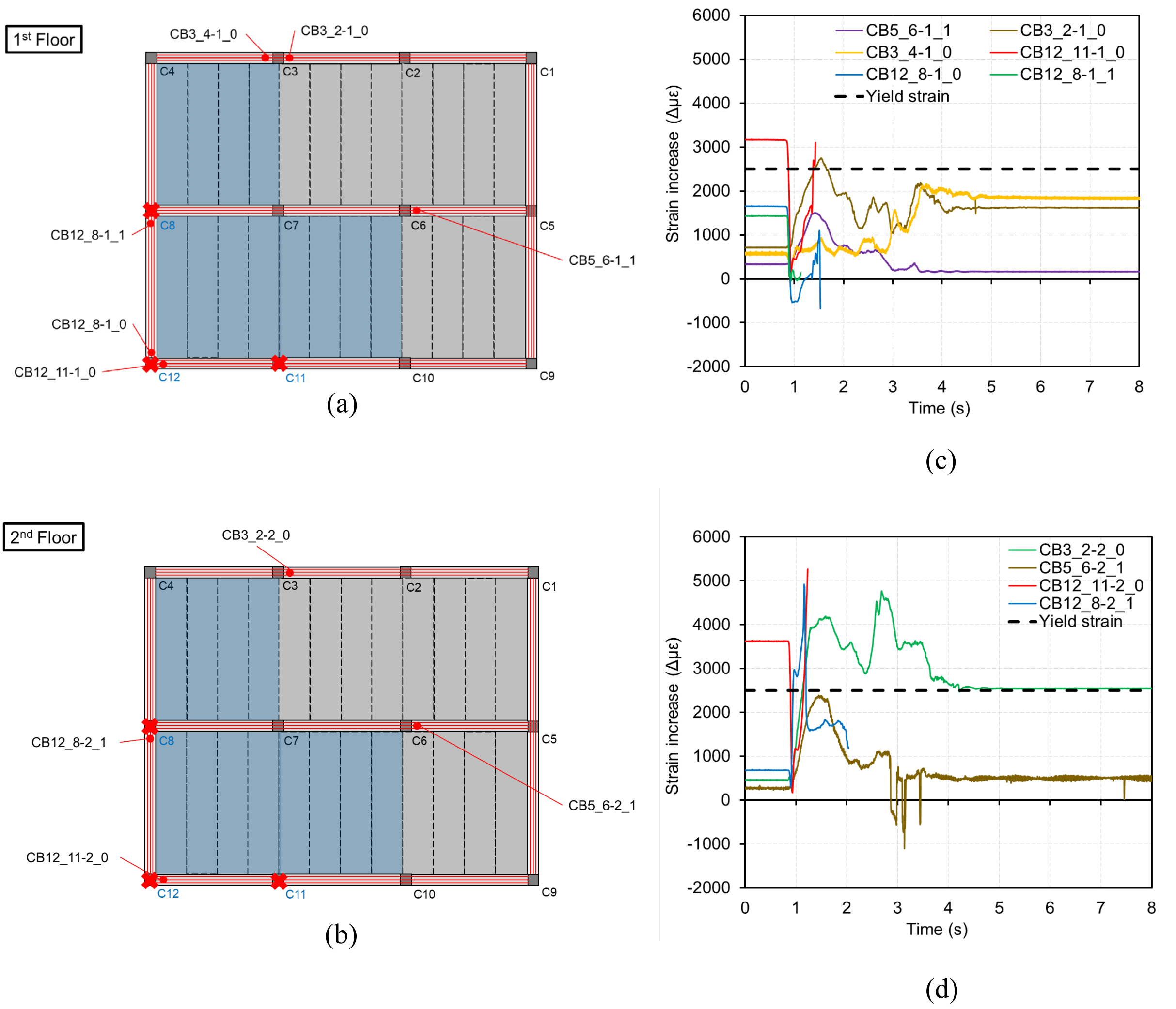

Supplementary Figure 25. **Layout of the monitored bars located close to the failure (segment) border in the (a) 1st Floor; (2) 2nd Floor; Strain increase in the continuity bars in the (c) 1st Floor; (d) 2nd Floor during Phase 2.**

Continuity bars connecting columns C12 to C11 and C8 (all three removed columns) were broken entirely during Phase 2 (CB12_8 and CB12_11). This indicates that the catenary action has been fully utilised and exhausted, leading to partial collapse. Simultaneously, a jump in strain was observed in all the rest of the continuity bars, followed by a gradual decrease to their original strains (unloading

behaviour) except for the continuity bars CB3_2-1_0, CB3_2-2_0, and CB3_4-1_0. These three bars were subjected to a significant residual strain after the vibration ceased, indicating permanent/plastic damages. This is expected as these three bars were located just on the border of the collapse bays, hence experiencing the most severe damage compared to other continuity bars. Comparing the increase in strain of the continuity bars on different floors, it was observed that the 2nd-floor bars experienced higher strains. The increase in CB3_2-2_0 (the second floor, close to the failure border) was beyond the expected yield strain.

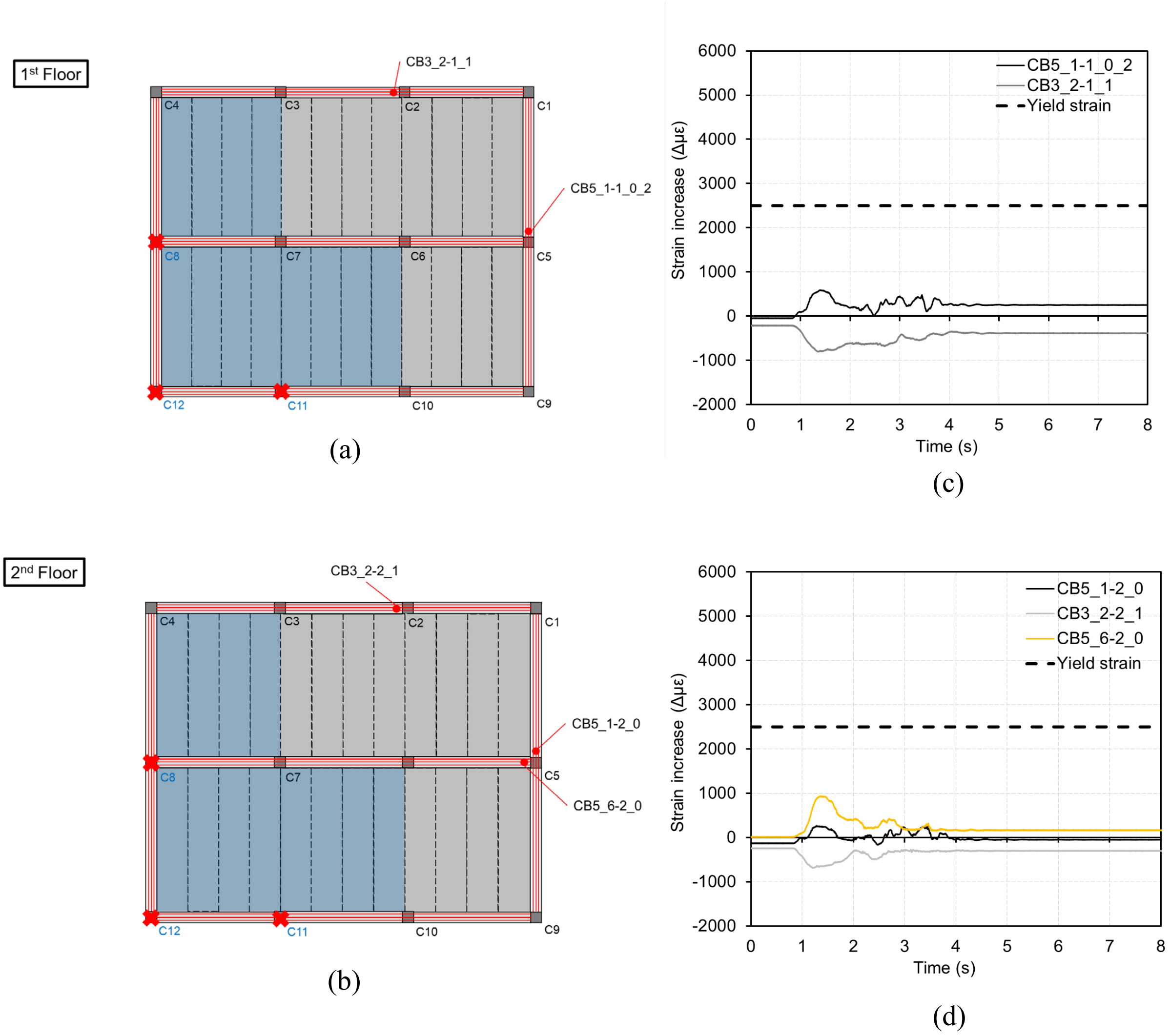

Supplementary Figure 26. **Layout of the monitored bars located farther from the failure (segment) border in the (a) 1st Floor; (2) 2nd Floor; Strain increase in the continuity bars in the (c) 1st Floor; (d) 2nd Floor during Phase 2.**

8. Floor accelerations in horizontal and vertical directions of the collapsing and the upright parts

In total, five accelerometers were installed in the building at a sample rate of 1000 samples per second: two sensors to measure the vertical acceleration at column C12 (collapsing part) and the middle bay located around columns C2, C3, C6, and C7; and another three sensors measuring the horizontal acceleration of the building at C9 for the X-direction of the building (both at the first and second-floor levels) and C1 for the Y-direction in the first floor (**S-Fig. 27a**). **S-Figs. 27b-e** provides the measured accelerations by these five sensors.

For these measurements, the value reported in **S-Fig. 27b-e** represents a duration of 100 s, which covers all the crucial phenomena during the collapse process (peak acceleration) and the decay of the vibration (post-peak acceleration). The largest acceleration was measured at column C12 (sensor A2-12Z, **S-Fig. 27b**) with a peak value of -3.3g and 1.6g, values which are clearly above a free fall of the building. This is somewhat expected as this accelerometer was placed at the collapsing part, experiencing the largest vibration. Nevertheless, the vertical acceleration was still high at the bays located around columns C2, C3, C6, and C7 (in the upright part but close to the failure/segment border), peaking at 1.2-1.3g (sensor A2-1Z, **S-Fig. 27d**). Regarding the horizontal acceleration, we observed that the second-floor level experienced a slightly larger acceleration than the first-floor one (**S-Fig. 27c**). Comparing the horizontal acceleration in X- and Y-directions, we can observe a comparable magnitude of 0.8-0.9g (**S-Figs. 27c, d**). This confirmed the severity of the collapse and how even the upright part that survived the collapse still experienced a significant impact.

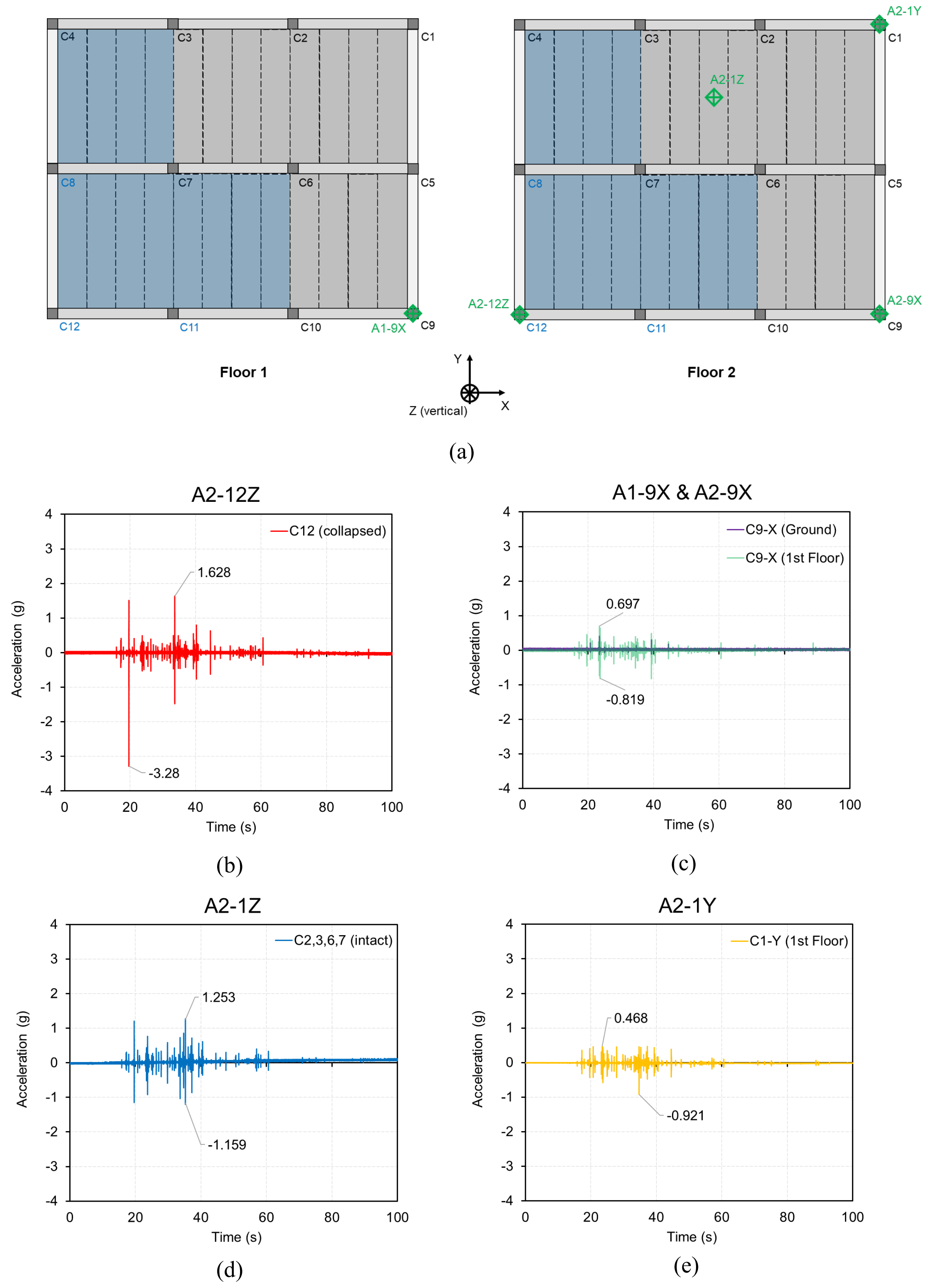

Supplementary Figure 27. **(a) The layout of the accelerometers; measured accelerations during Phase 2 at (b) column C12, second floor (vertical, Z-direction); (c) C9, first and second floors (horizontal, X-direction); (d) floor slabs located at the bays between C2, C3, C6, and C7, second floor (vertical, Z-direction); (e) column C1, second floor (horizontal, Y-direction).**

References

[1] EN 1992-1-1. Eurocode 2 - Design of concrete structures - Part 1-1: General rules and rules for buildings. 2004.

[2] EN 1990. Eurocode 0 - Basis of structural design. 2002.

[3] EN 1991-1-1. Eurocode 1 - Actions on structures - Part 1-1: Densities, self-weight, imposed loads for buildings. 2003.

[4] EN1991-1-7:2006: Eurocode 1 - Actions on structures - Part 1-7: General actions - accidental actions. 2006.

[5] The Institution of Structural Engineers. Practical guide to structural robustness and disproportionate collapse in buildings. 2010.

[6] FIB Commission 6. Guide to good practice: Design of precast concrete structures against accidental actions. 2012.

[7] FIB Commission 6. Guide to good practice: Structural connections for precast concrete buildings. 2008.

[8] ASCE 7-22. Minimum design loads and associated criteria for buildings and other structures. 2022.

Appendix
Detailed drawing of the as-built building design

Detailed construction drawings of the as-built design are included in the following pages with the following index of contents:

1. Plan view.
2. Foundation.
3. Foundation details (1).
4. Foundation details (2).
5. Foundation details (3).
6. Foundation details (4).
7. Foundation details (5).
8. Foundation details (6).
9. Foundation details (7).
10. Columns section view.
11. Corner columns – Ground floor.
12. Corner column C12 – Ground floor.
13. Corner columns – First floor,
14. Edge columns – Long side – Ground floor.
15. Edge column C3 – Long side – Ground floor.
16. Edge columns – Long side – First floor.
17. Edge columns – Short side – Ground floor.
18. Edge column C5 – Short side – Ground floor.
19. Edge columns – Short side – First floor.
20. Internal columns – Ground floor.
21. Internal columns – First floor.
22. Precast beams and external frames assemblage.
23. Precast beams and internal frames assemblage.
24. Precast beams and edge frames assemblage.
25. Slabs 1 and 2 – Reinforcements and tying (1).
26. Slabs 1 and 2 – Reinforcements and tying (2).

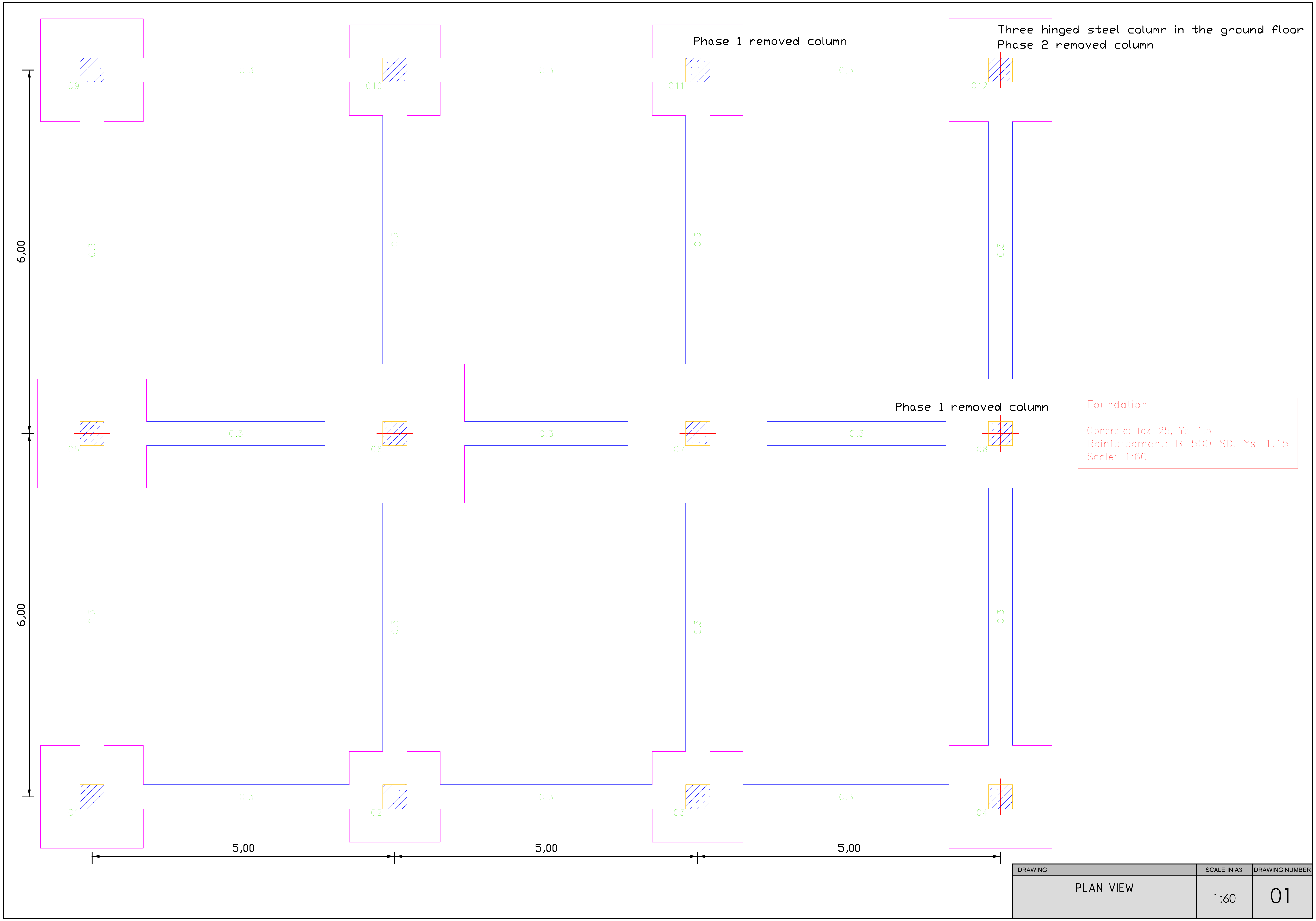
Three hinged steel column in the ground floor
Phase 2 removed column
Phase 1 removed column
Phase 1 removed column
Foundation
Concrete: fck=25, Yc=1.5
Reinforcement: B 500 SD, Ys=1.15
Scale: 1:60
6,00
6,00
5,00
5,00
5,00
C.3
C1
C2
C3
C4
C5
C6
C7
C8
C9
C10
C11
C12
DRAWING
PLAN VIEW
SCALE IN A3
1:60
DRAWING NUMBER
01

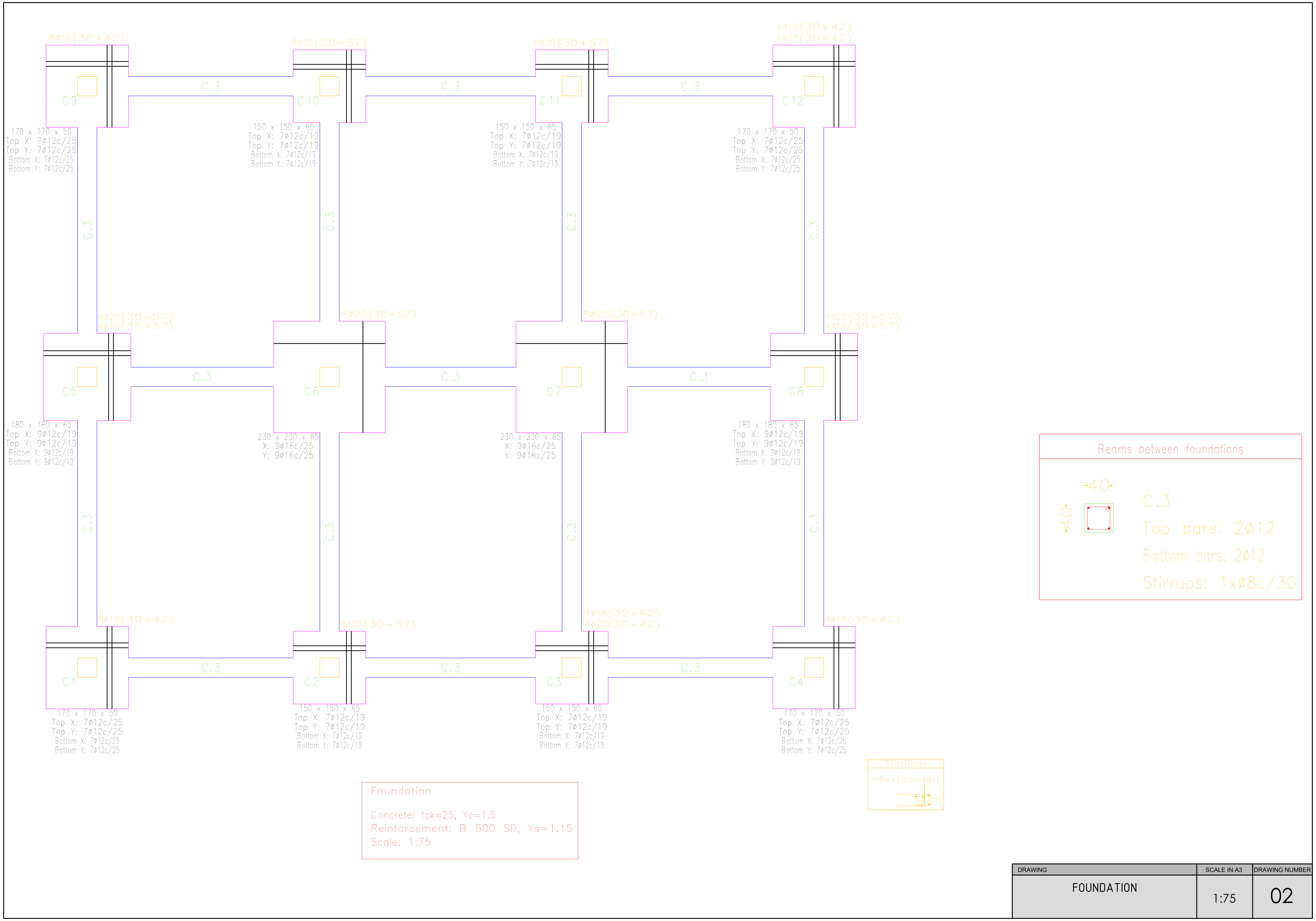

8ø16(30+42)
8ø20(30+57)
8ø20(30+57)
4ø16(30+42)
4ø20(30+42)
C.3
C.3
C.3
C9
C10
C11
C12
170 x 170 x 50
Top X: 7ø12c/25
Top Y: 7ø12c/25
Bottom X: 7ø12c/25
Bottom Y: 7ø12c/25
150 x 150 x 65
Top X: 7ø12c/19
Top Y: 7ø12c/19
Bottom X: 7ø12c/19
Bottom Y: 7ø12c/19
4ø20(30+57)
4ø16(30+57)
8ø25(30+57)
8ø25(30+57)
C5
C6
C7
C8
180 x 180 x 65
Top X: 9ø12c/19
Top Y: 9ø12c/19
Bottom X: 9ø12c/19
Bottom Y: 9ø12c/19
230 x 230 x 65
X: 9ø16c/25
Y: 9ø16c/25
8ø16(30+42)
8ø20(30+57)
C1
C2
C3
C4
Beams between foundations
40
C.3
Top bars: 2ø12
Bottom bars: 2ø12
Stirrups: 1xø8c/30
Stirrups
nøxx(aa+bb)
Foundation
Concrete: fck=25, Yc=1.5
Reinforcement: B 500 SD, Ys=1.15
Scale: 1:75
DRAWING
FOUNDATION
SCALE IN A3
1:75
DRAWING NUMBER
02

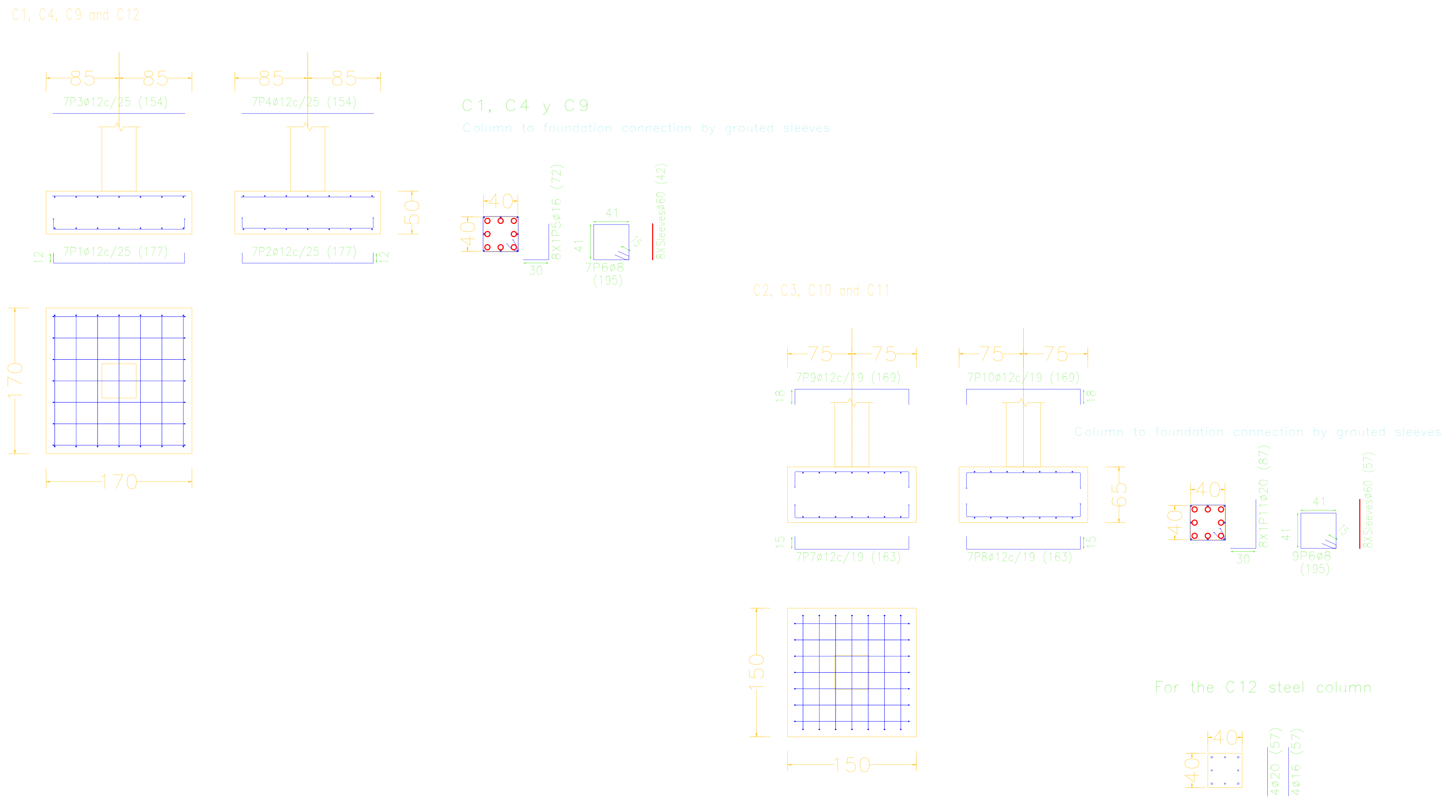

DRAWING	SCALE IN A3	DRAWING NUMBER
FOUNDATION DETAILS (1)	1:50	03

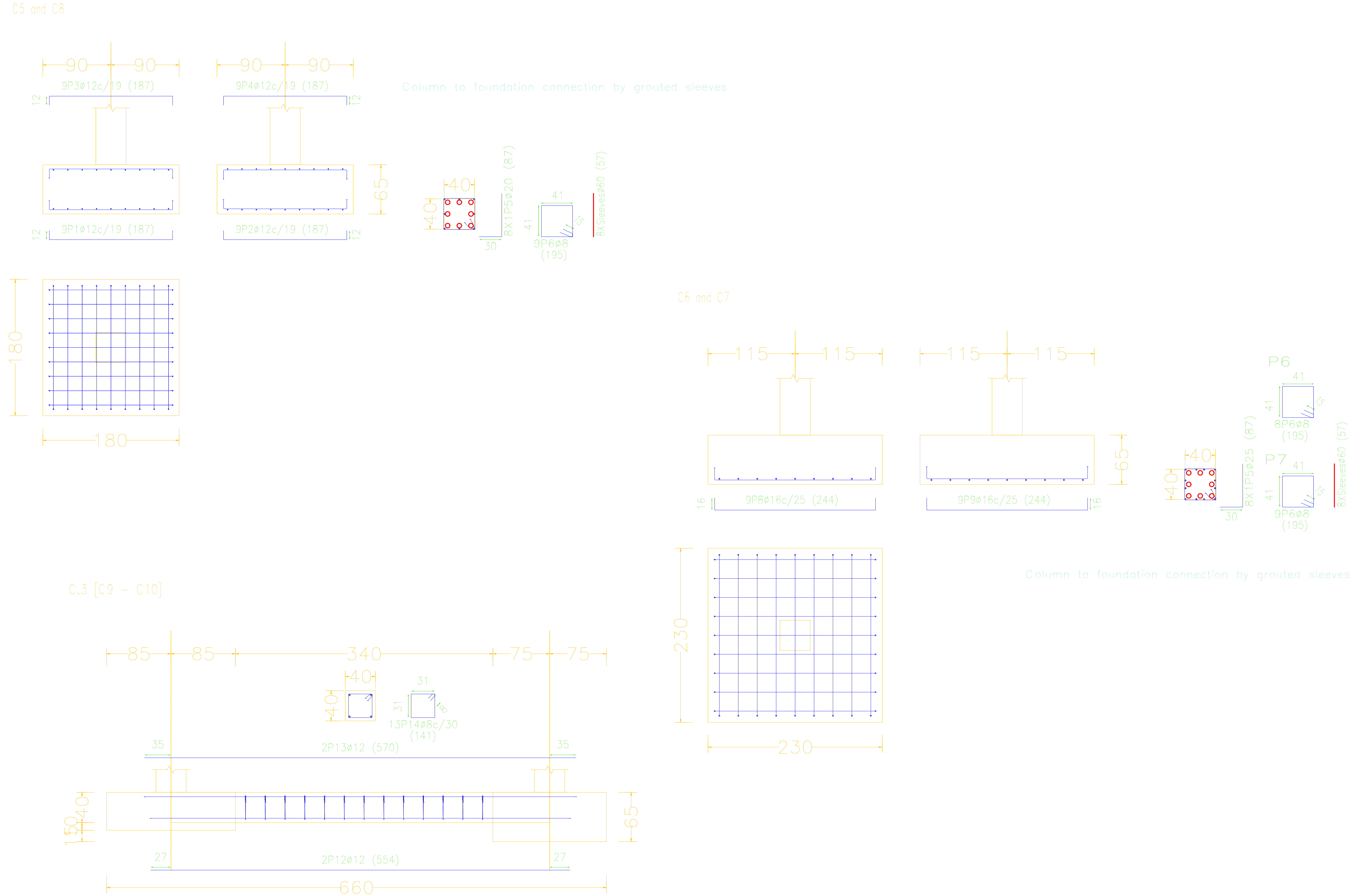

DRAWING	SCALE IN A3	DRAWING NUMBER
FOUNDATION DETAILS (2)	1:50	04

C.3 [C5 - C9]

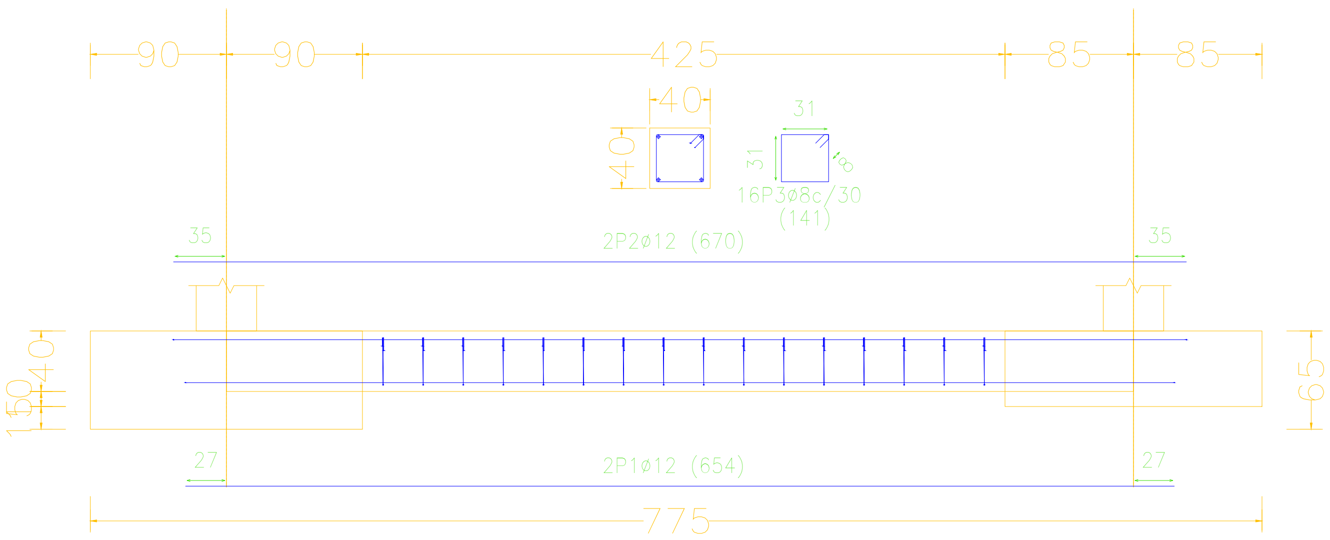

C.3 [C10 - C11]

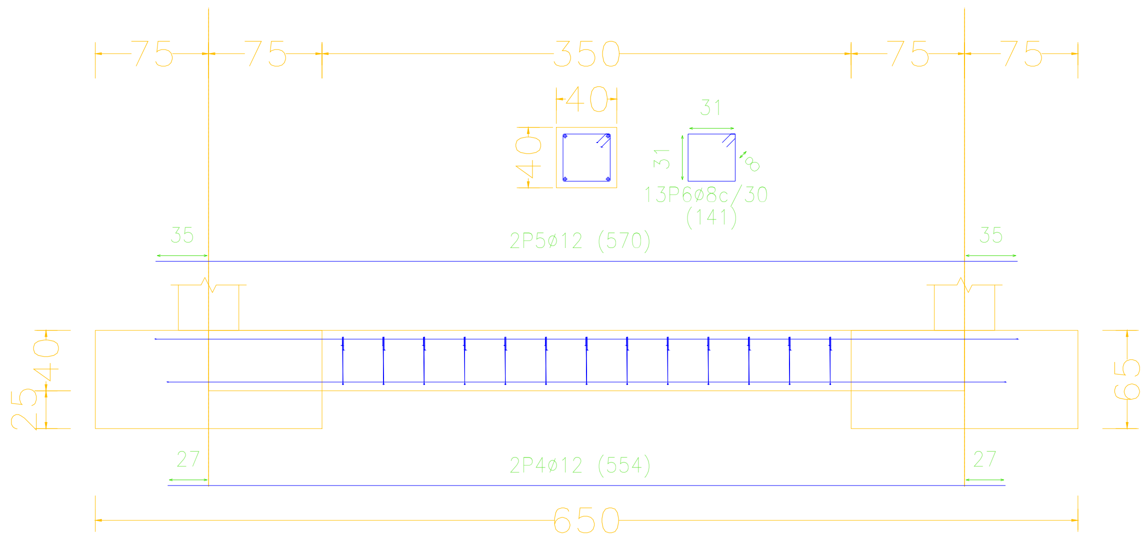

C.3 [C6 - C10]

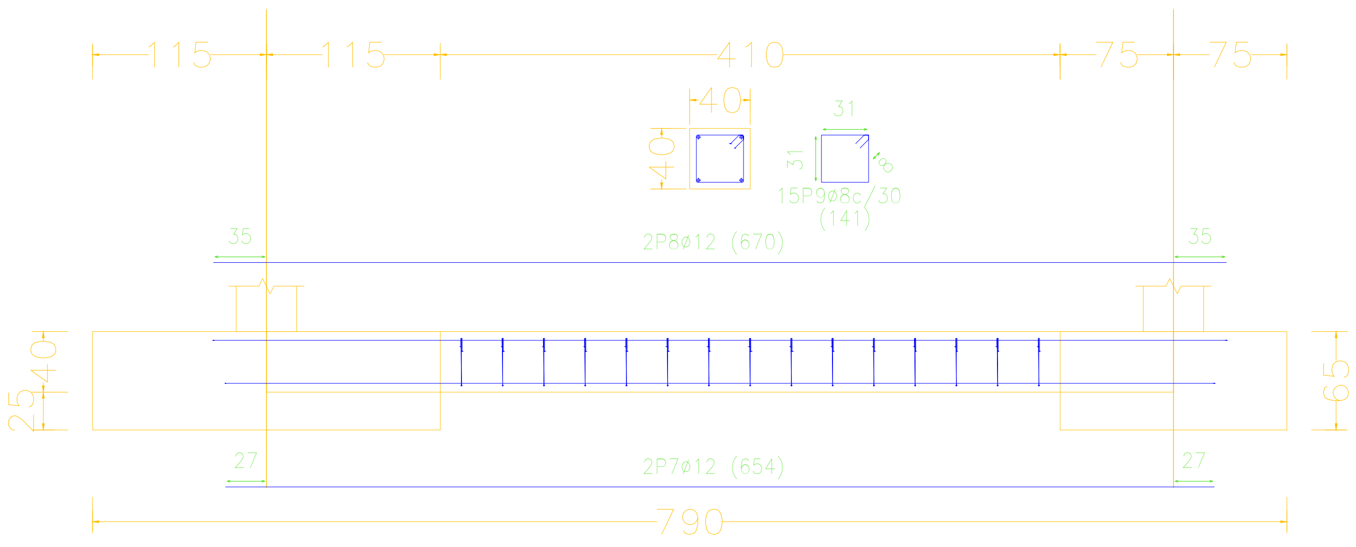

DRAWING	SCALE IN A3	DRAWING NUMBER
FOUNDATION DETAILS (3)	1:50	05

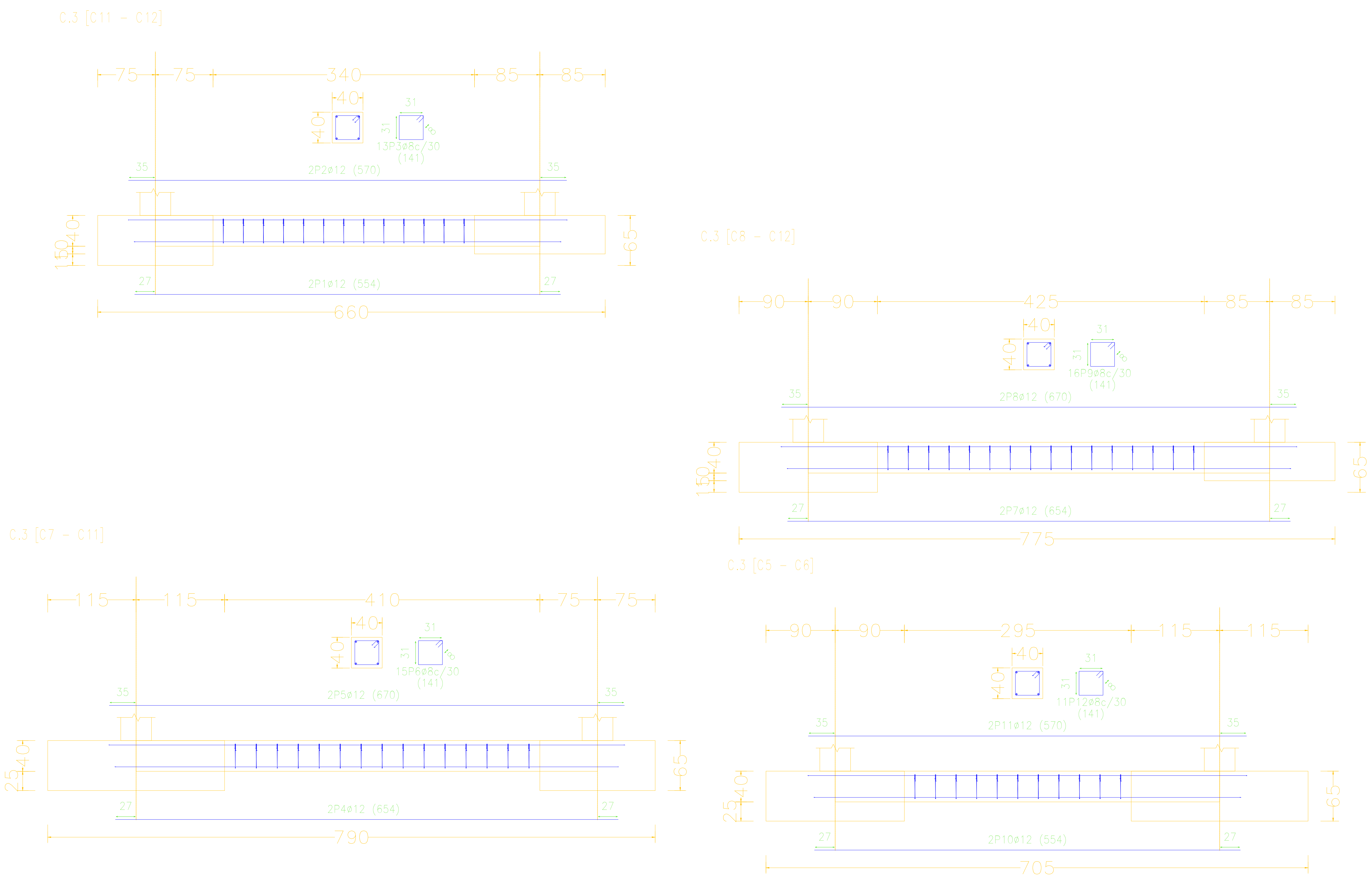

DRAWING	SCALE IN A3	DRAWING NUMBER
FOUNDATION DETAILS (4)	1:50	06

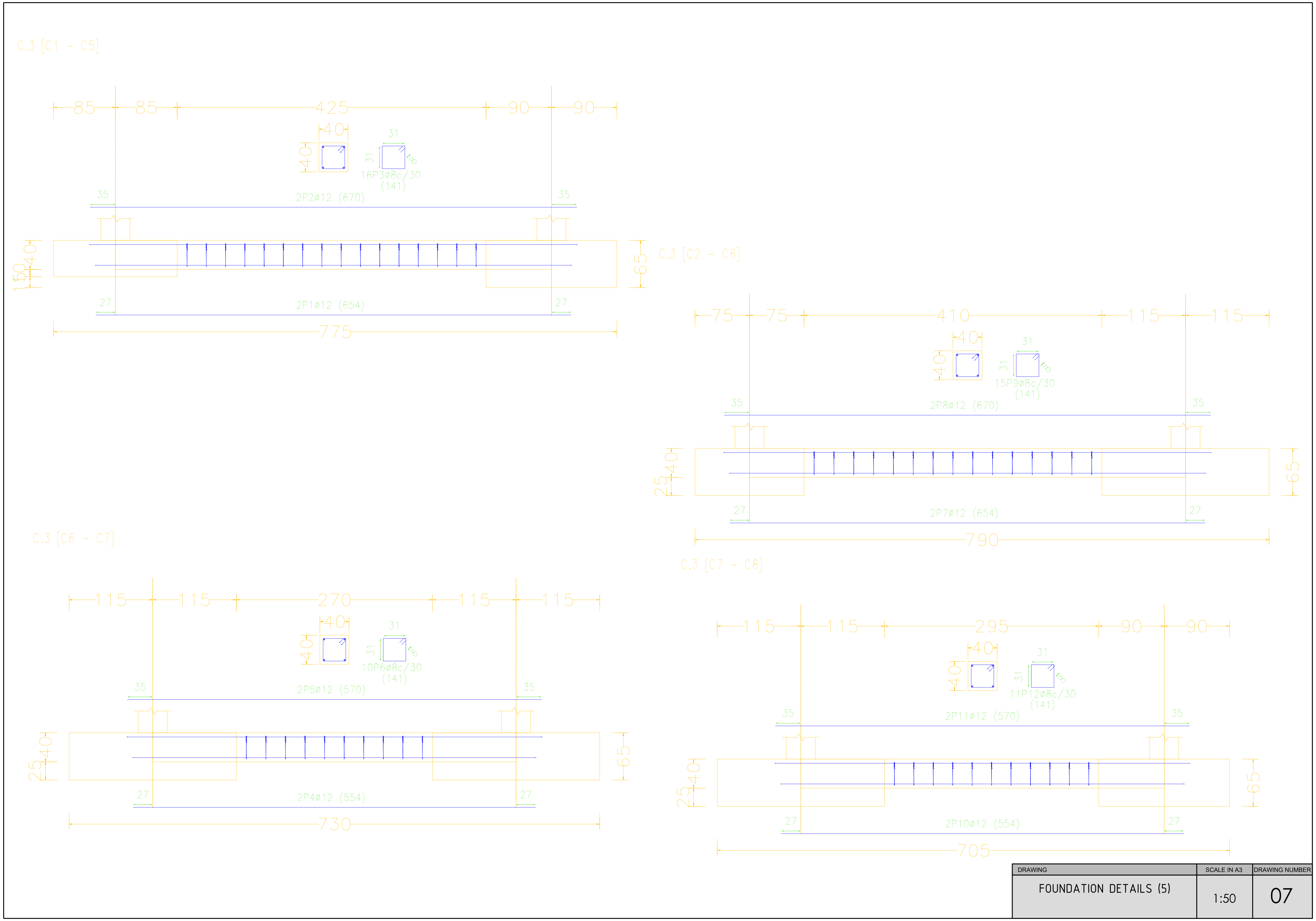
C.3 [C1 - C5]
16P3ø8c/30
(141)
2P2ø12 (670)
2P1ø12 (654)
C.3 [C2 - C8]
15P9ø8c/30
(141)
2P8ø12 (670)
2P7ø12 (654)
C.3 [C6 - C7]
10P6ø8c/30
(141)
2P5ø12 (570)
2P4ø12 (554)
C.3 [C7 - C8]
11P12ø8c/30
(141)
2P11ø12 (570)
2P10ø12 (554)
DRAWING
FOUNDATION DETAILS (5)
SCALE IN A3
1:50
DRAWING NUMBER
07

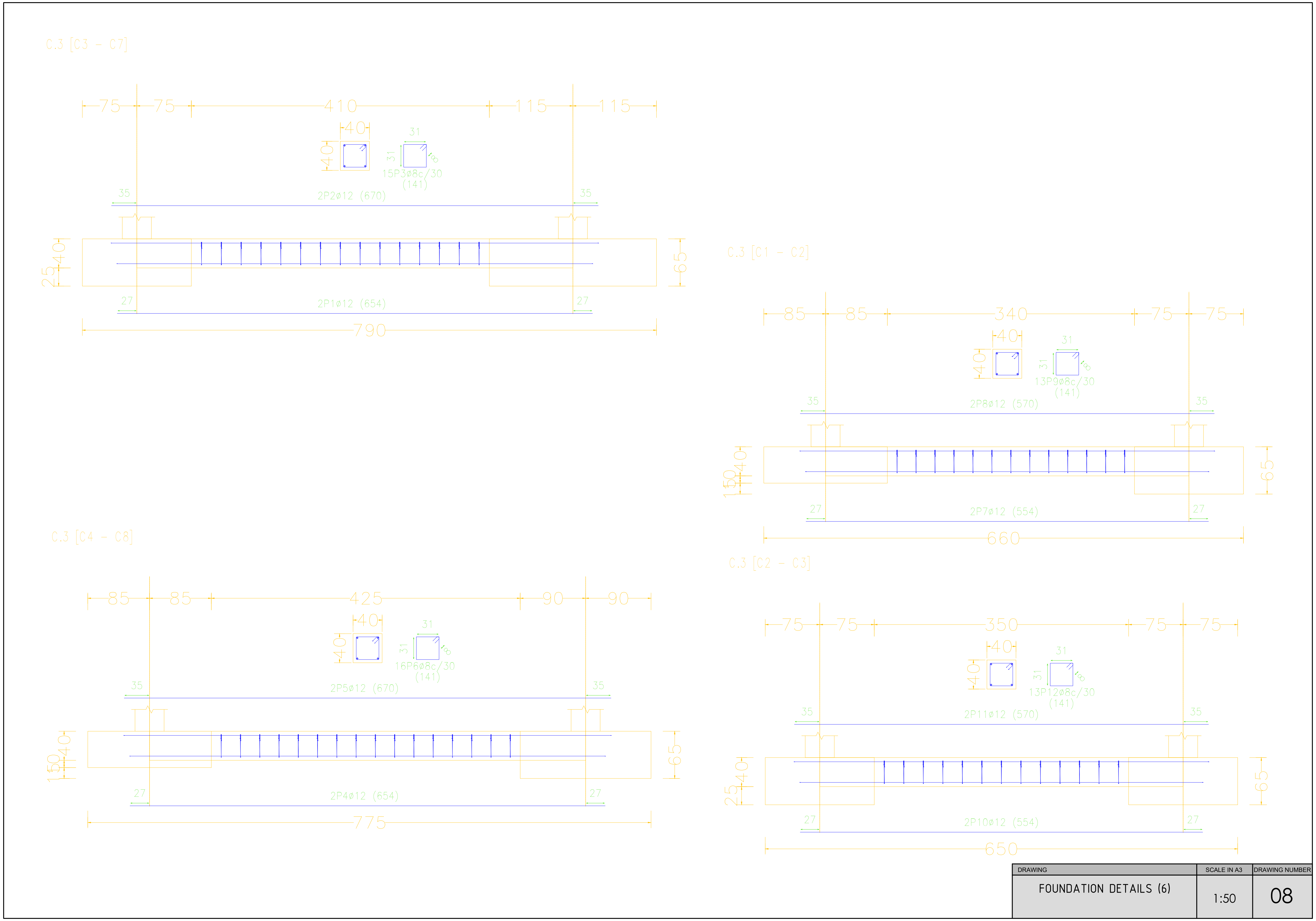
C.3 [C3 - C7]
15P3ø8c/30
(141)
2P2ø12 (670)
2P1ø12 (654)
790
C.3 [C1 - C2]
13P9ø8c/30
(141)
2P8ø12 (570)
2P7ø12 (554)
660
C.3 [C4 - C8]
16P6ø8c/30
(141)
2P5ø12 (670)
2P4ø12 (654)
775
C.3 [C2 - C3]
13P12ø8c/30
(141)
2P11ø12 (570)
2P10ø12 (554)
650
DRAWING
FOUNDATION DETAILS (6)
SCALE IN A3
1:50
DRAWING NUMBER
08

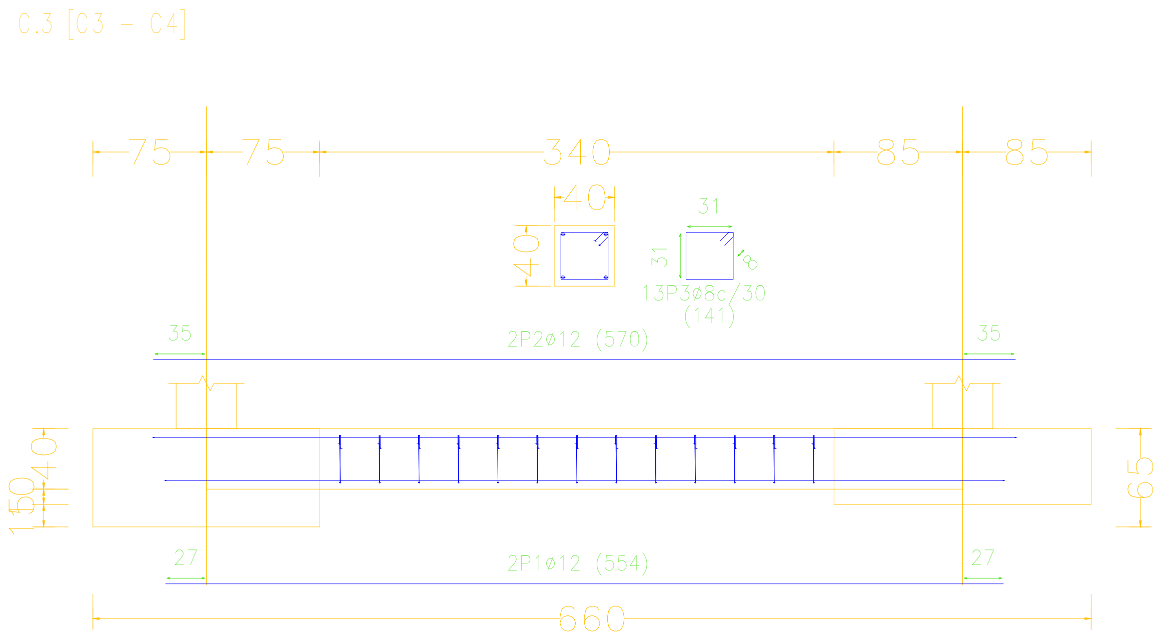

DRAWING	SCALE IN A3	DRAWING NUMBER
FOUNDATION DETAILS (7)	1:50	09

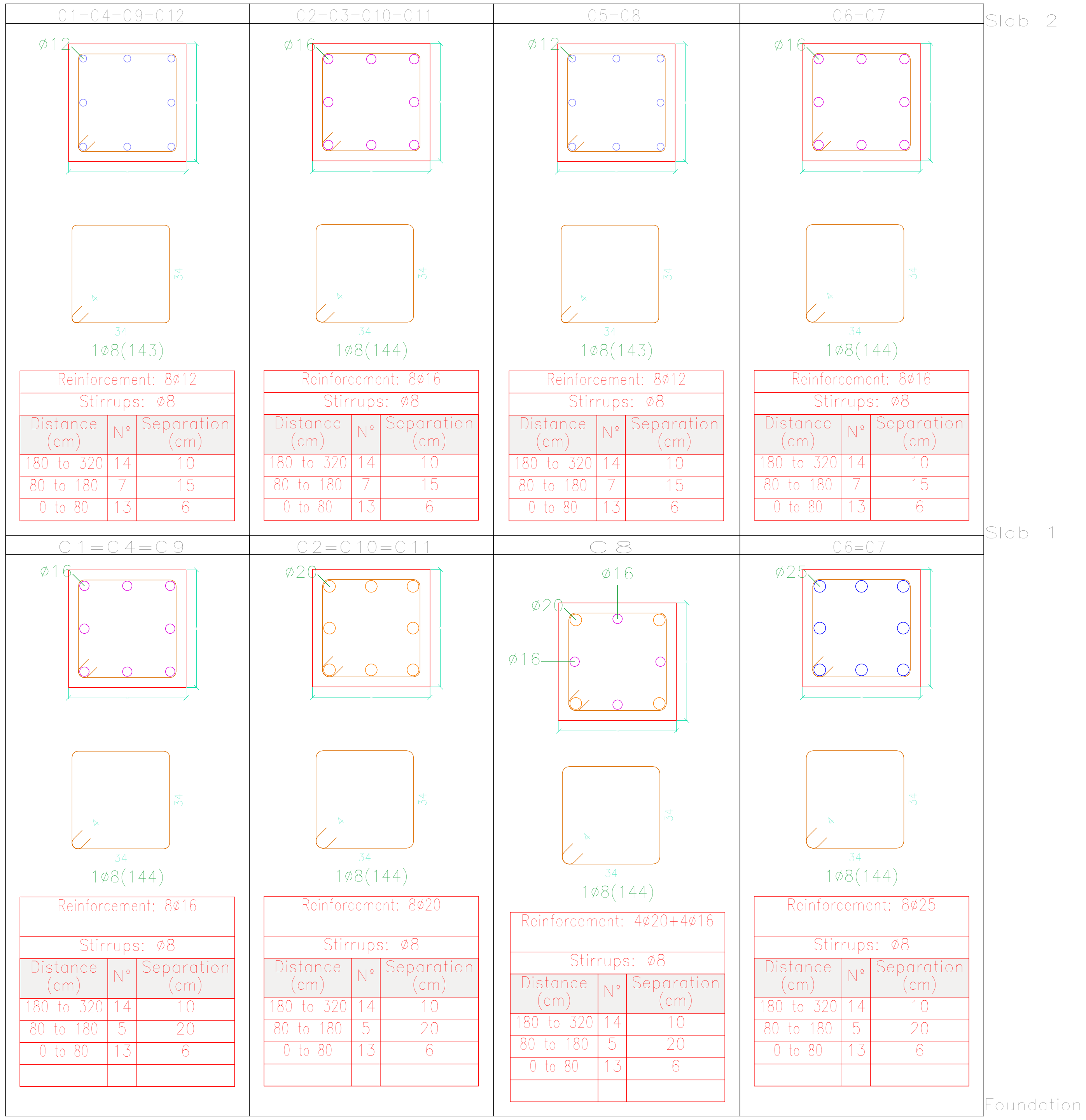

Columns; section view
Scale 1:20
Concrete: fck=30, Yc=1.5
Reinforcement: B 500 SD, Ys=1.15

DRAWING	SCALE IN A3	DRAWING NUMBER
COLUMNS SECTION VIEW	1:20	10

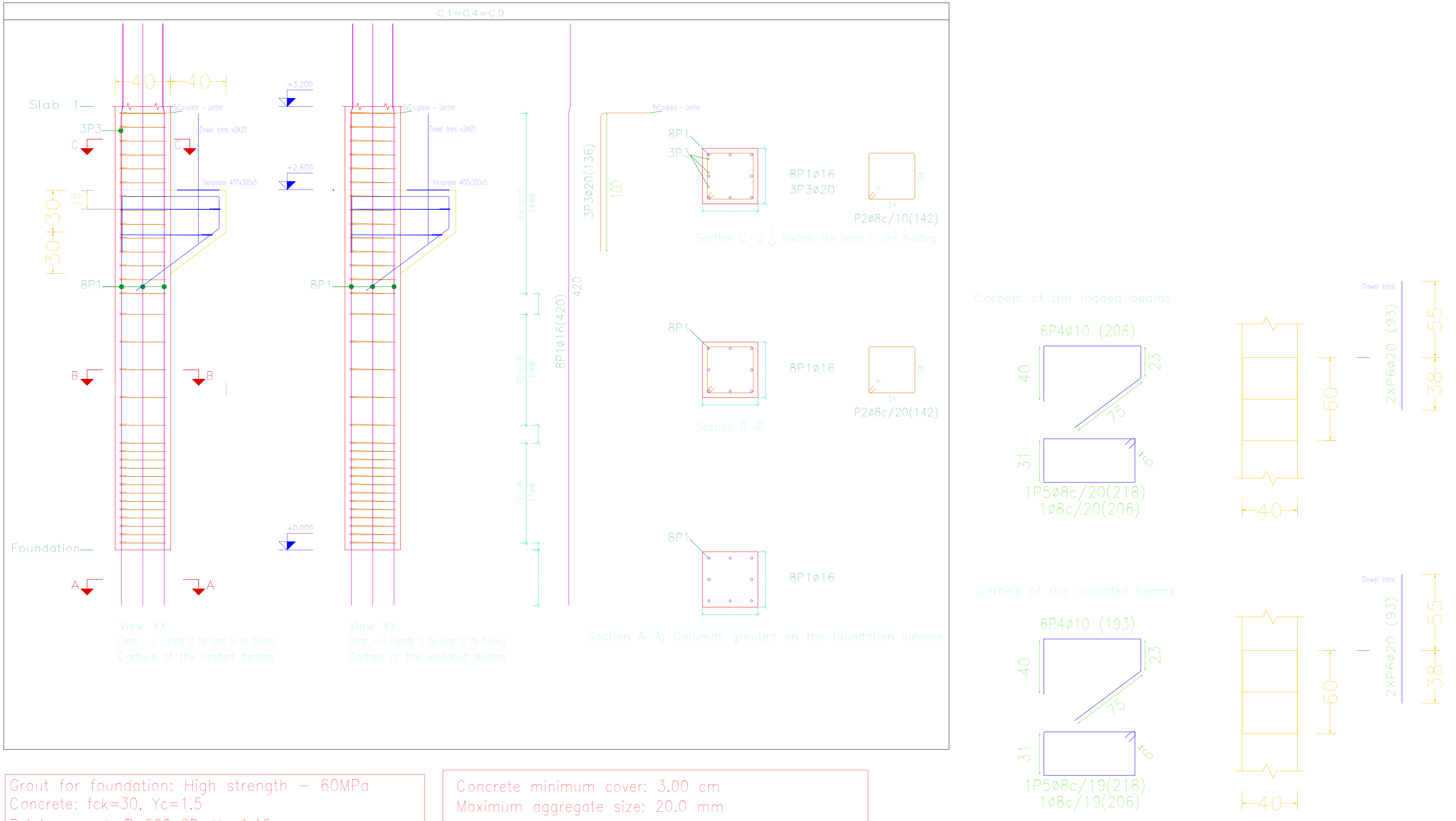

Grout for foundation: High strength – 60MPa
Concrete: fck=30, Yc=1.5
Reinforcement: B 500 SD, Ys=1.15

Concrete minimum cover: 3.00 cm
Maximum aggregate size: 20.0 mm

Scale: 1: 30

DRAWING	SCALE IN A3	DRAWING NUMBER
CORNER COLUMNS – GROUND FLOOR	1:30	11

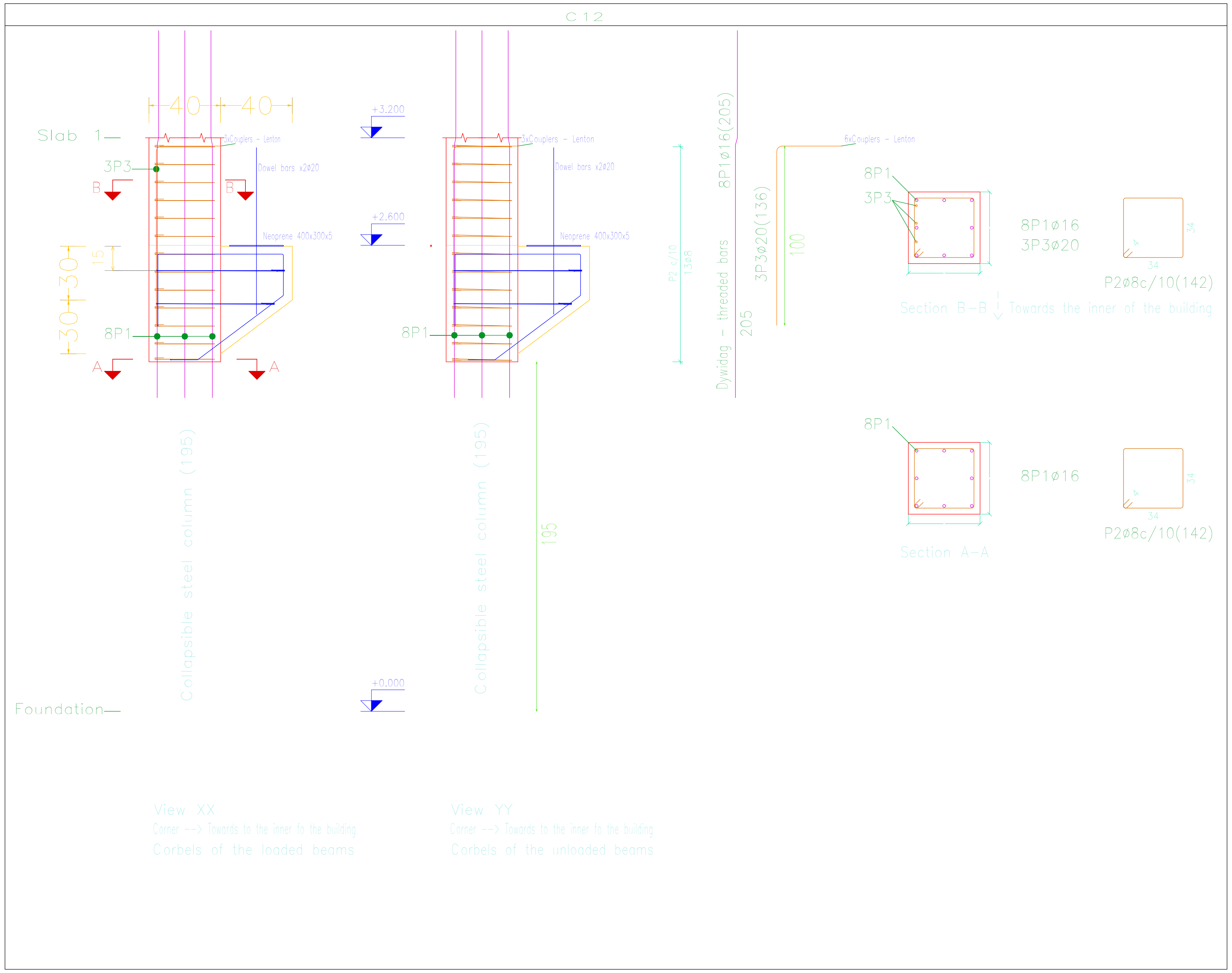

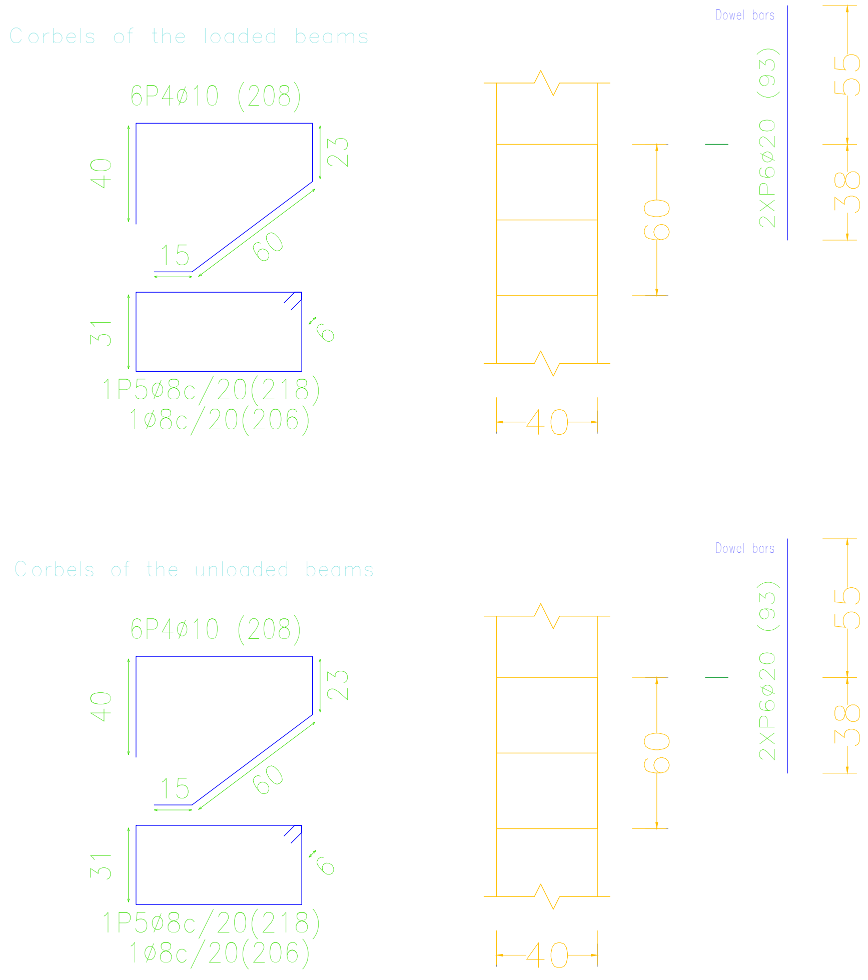

Grout for foundation: High strength - 60MPa
Concrete: fck=30, Yc=1.5
Reinforcement: B 500 SD, Ys=1.15

Concrete minimum cover: 3.00 cm
Maximum aggregate size: 20.0 mm

Scale: 1: 30

DRAWING	SCALE IN A3	DRAWING NUMBER
CORNER COLUMN C12 - GROUND FLOOR	1:30	12

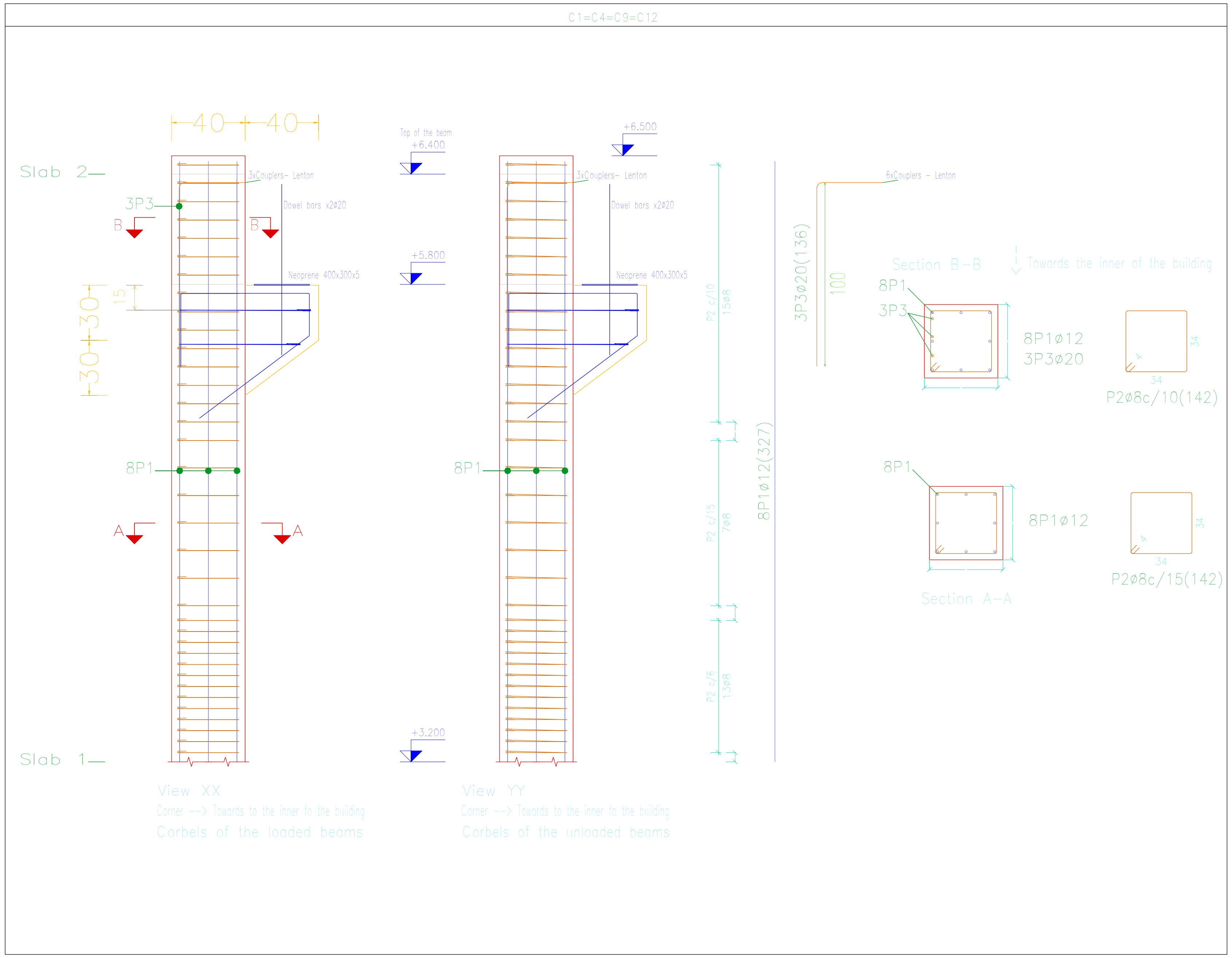

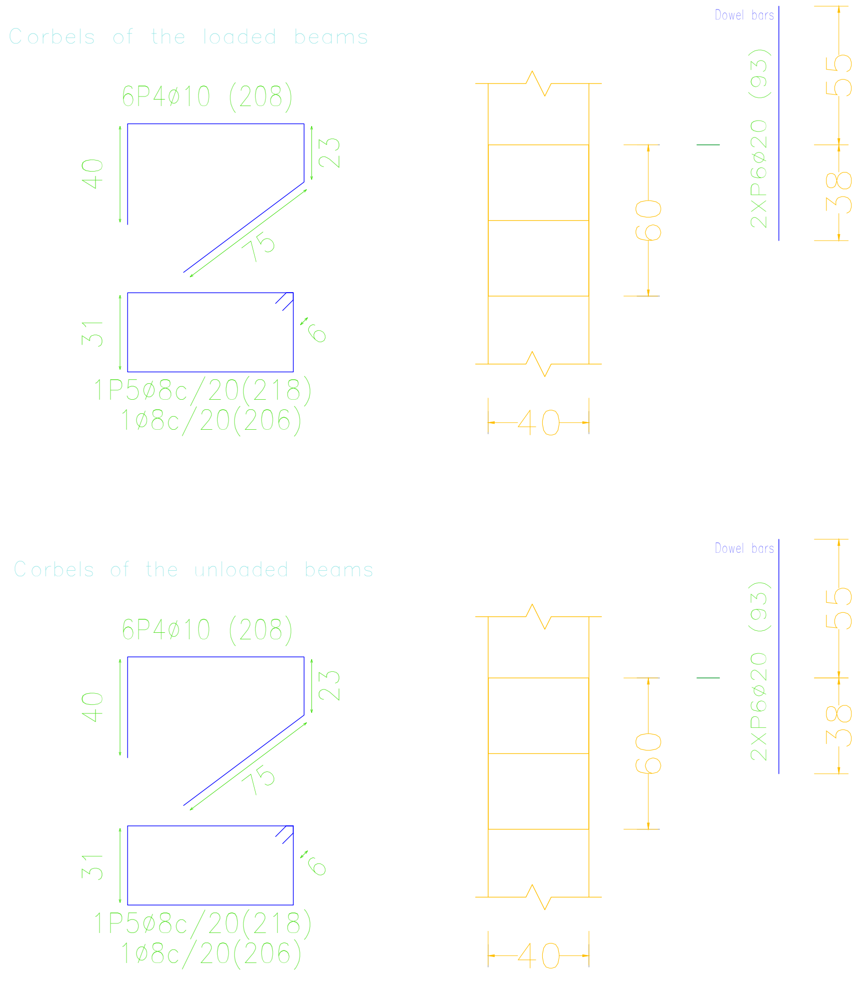

Grout for foundation: High strength – 60MPa
Concrete: fck=30, Yc=1.5
Reinforcement: B 500 SD, Ys=1.15

Concrete minimum cover: 3.00 cm
Maximum aggregate size: 20.0 mm

Scale: 1: 30

DRAWING	SCALE IN A3	DRAWING NUMBER
CORNER COLUMNS – FIRST FLOOR	1:30	13

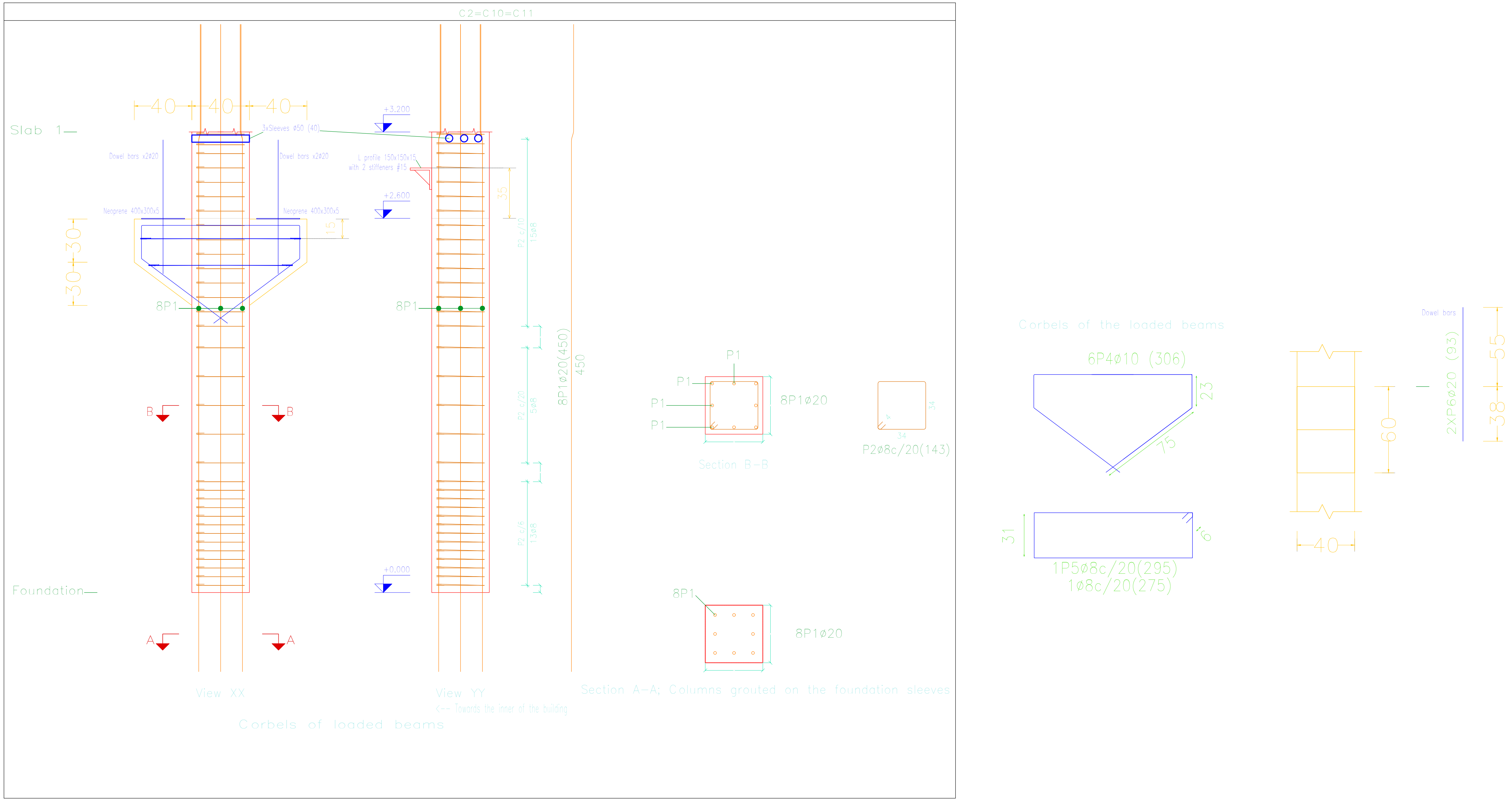

Grout for foundation: High strength – 60MPa
Concrete: fck=30, Yc=1.5
Reinforcement: B 500 SD, Ys=1.15

Concrete minimum cover: 3.00 cm
Maximum aggregate size: 20.0 mm

Scale: 1: 30

DRAWING	SCALE IN A3	DRAWING NUMBER
EDGE COLUMNS – LONG SIDE – GROUND FLOOR	1:30	14

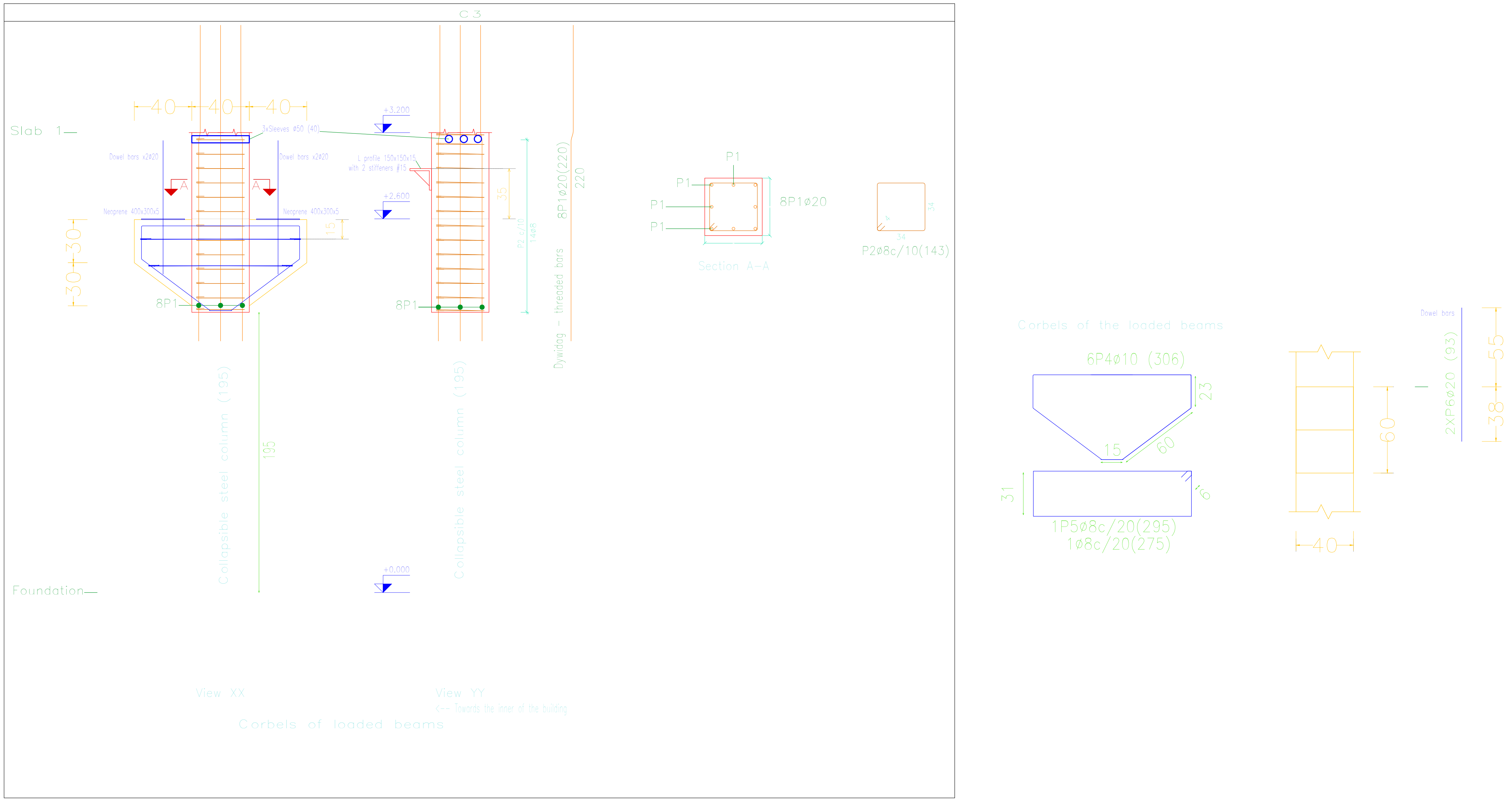

Grout for foundation: High strength – 60MPa
Concrete: fck=30, Yc=1.5
Reinforcement: B 500 SD, Ys=1.15

Concrete minimum cover: 3.00 cm
Maximum aggregate size: 20.0 mm

Scale: 1: 30

DRAWING	SCALE IN A3	DRAWING NUMBER
EDGE COLUMN C3 – LONG SIDE – GROUND FLOOR	1:30	15

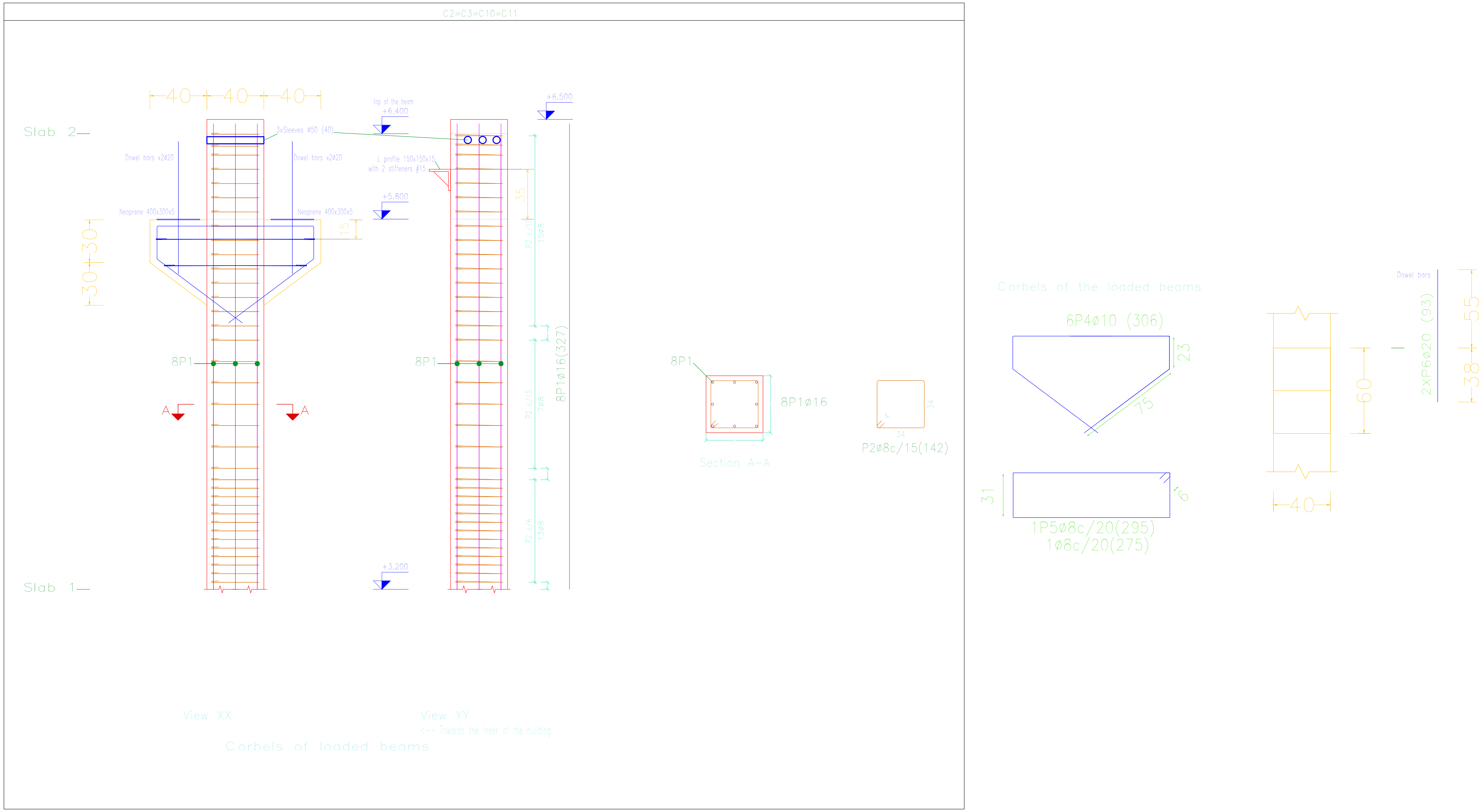

Grout for foundation: High strength – 60MPa
Concrete: fck=30, Yc=1.5
Reinforcement: B 500 SD, Ys=1.15

Concrete minimum cover: 3.00 cm
Maximum aggregate size: 20.0 mm

Scale: 1: 30

DRAWING	SCALE IN A3	DRAWING NUMBER
EDGE COLUMNS – LONG SIDE – FIRST FLOOR	1:30	16

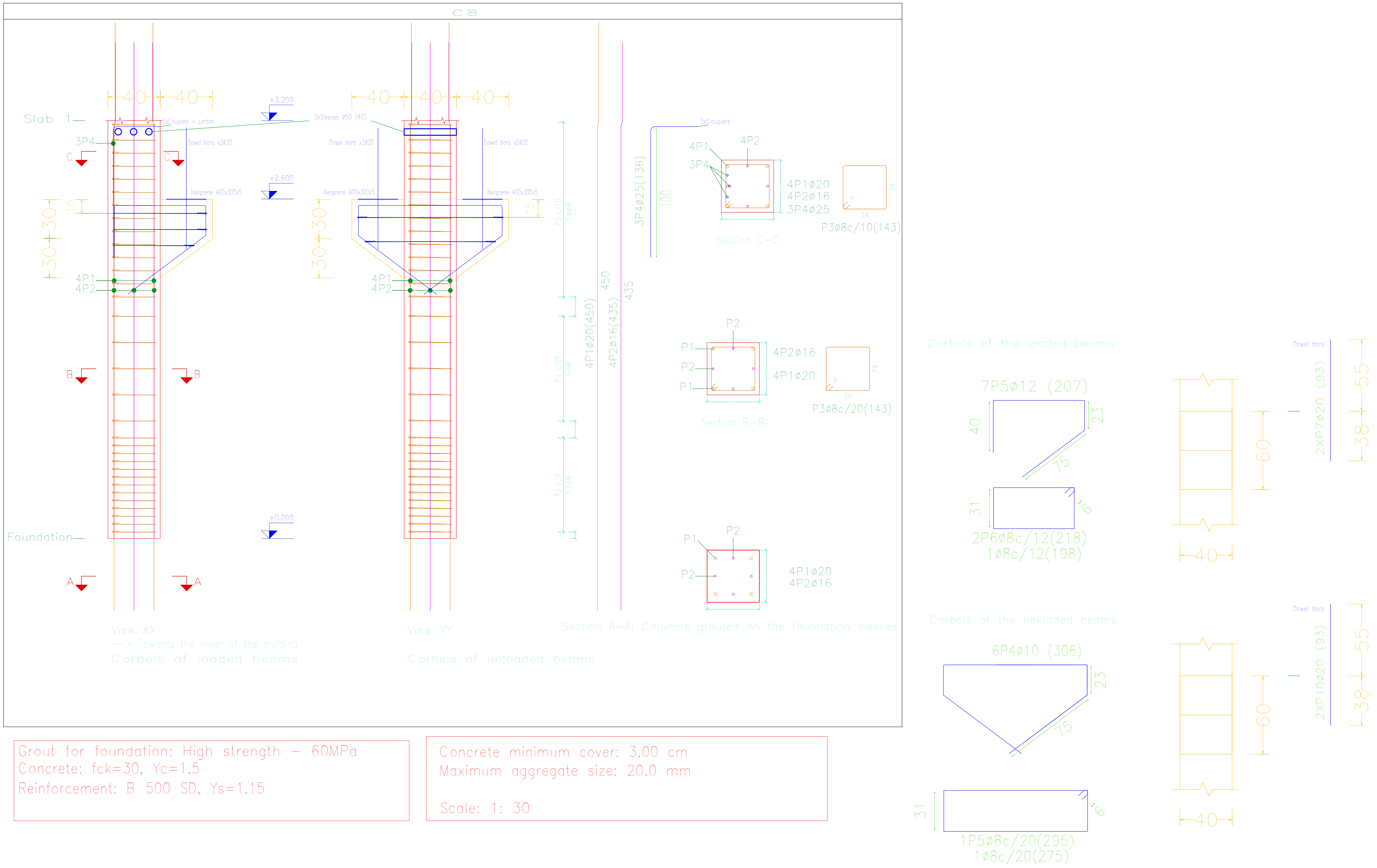

DRAWING	SCALE IN A3	DRAWING NUMBER
EDGE COLUMNS – SHORT SIDE – GROUND FLOOR	1:30	17

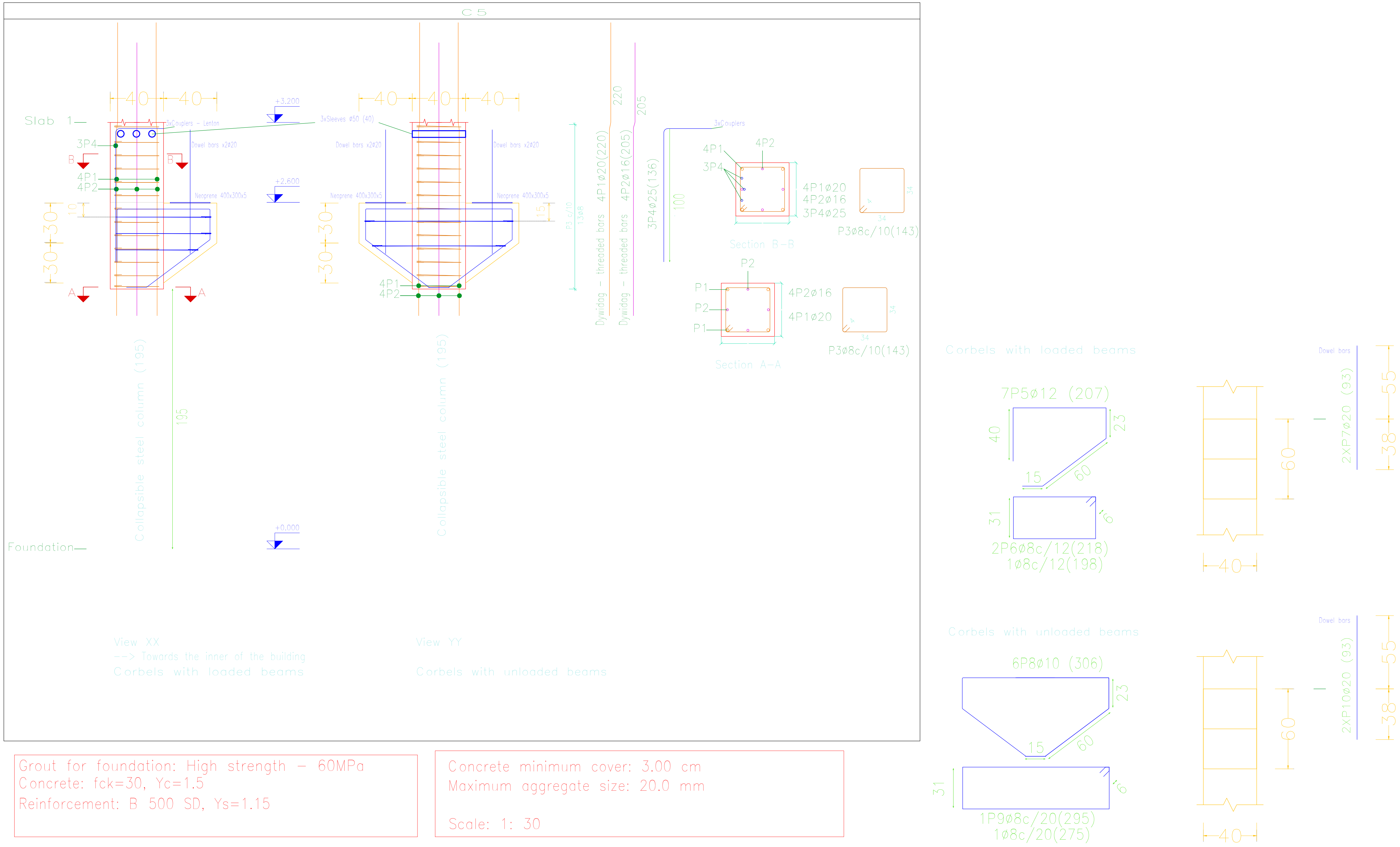

Grout for foundation: High strength – 60MPa
Concrete: fck=30, Yc=1.5
Reinforcement: B 500 SD, Ys=1.15

Concrete minimum cover: 3.00 cm
Maximum aggregate size: 20.0 mm

Scale: 1: 30

DRAWING	SCALE IN A3	DRAWING NUMBER
COLUMN C5 - SHORT SIDE - GROUND FLOOR	1:30	18

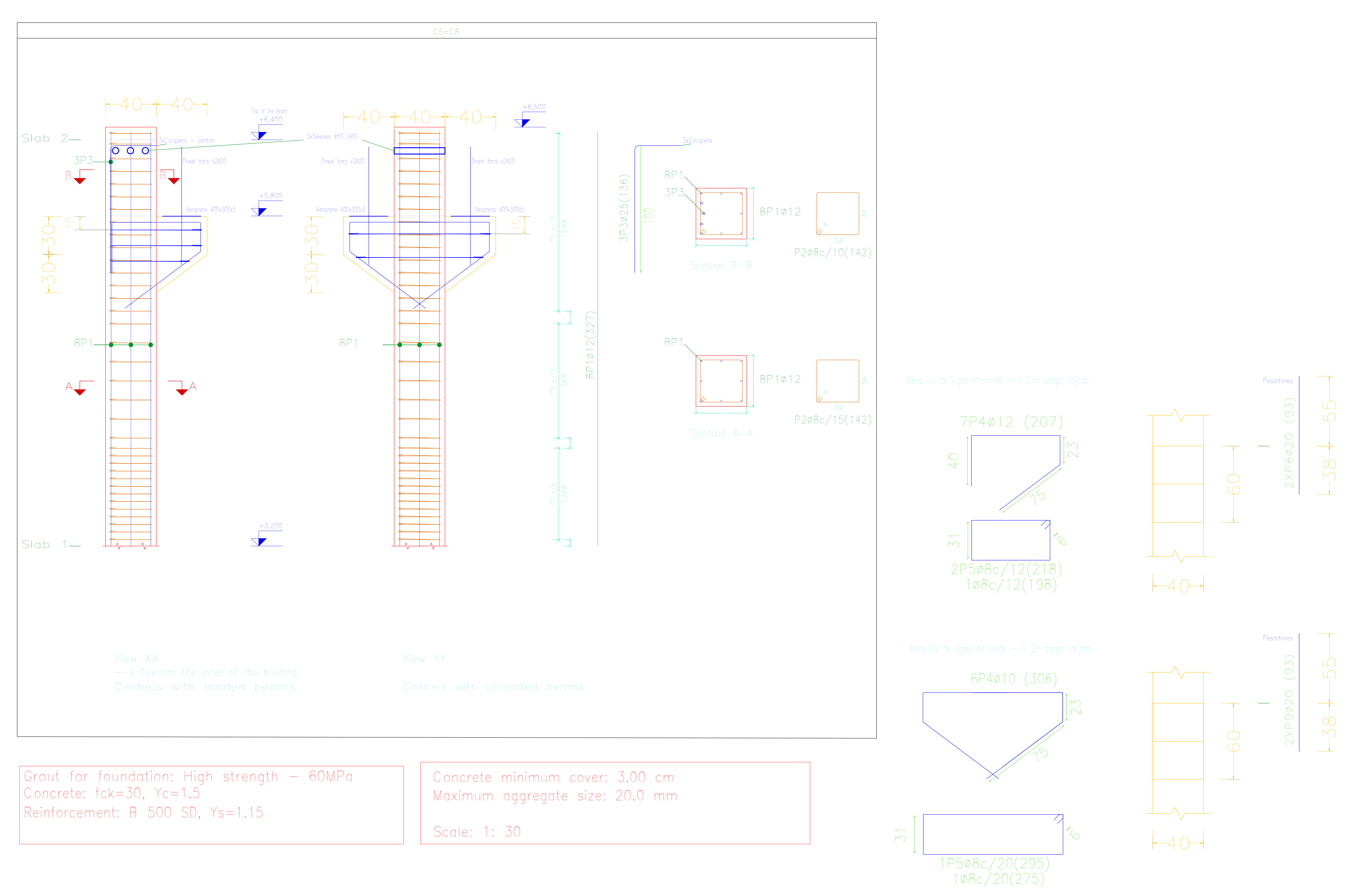

DRAWING	SCALE IN A3	DRAWING NUMBER
EDGE COLUMNS – SHORT SIDE – FIRST FLOOR	1:30	19

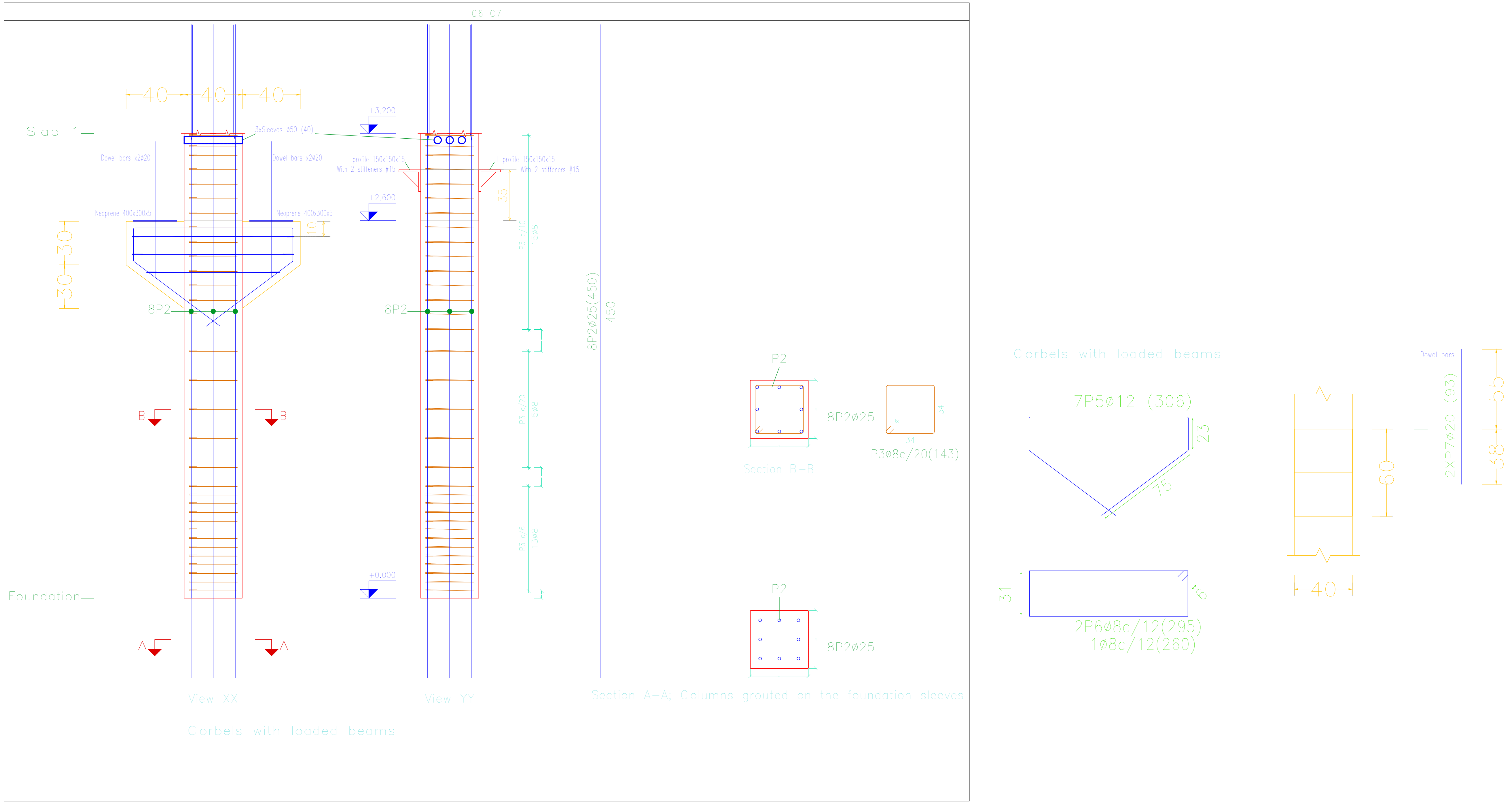

Grout for foundation: High strength – 60MPa
Concrete: fck=30, Yc=1.5
Reinforcement: B 500 SD, Ys=1.15

Concrete minimum cover: 3.00 cm
Maximum aggregate size: 20.0 mm

Scale: 1: 30

DRAWING	SCALE IN A3	DRAWING NUMBER
INTERNAL COLUMNS – GROUND FLOOR	1:30	20

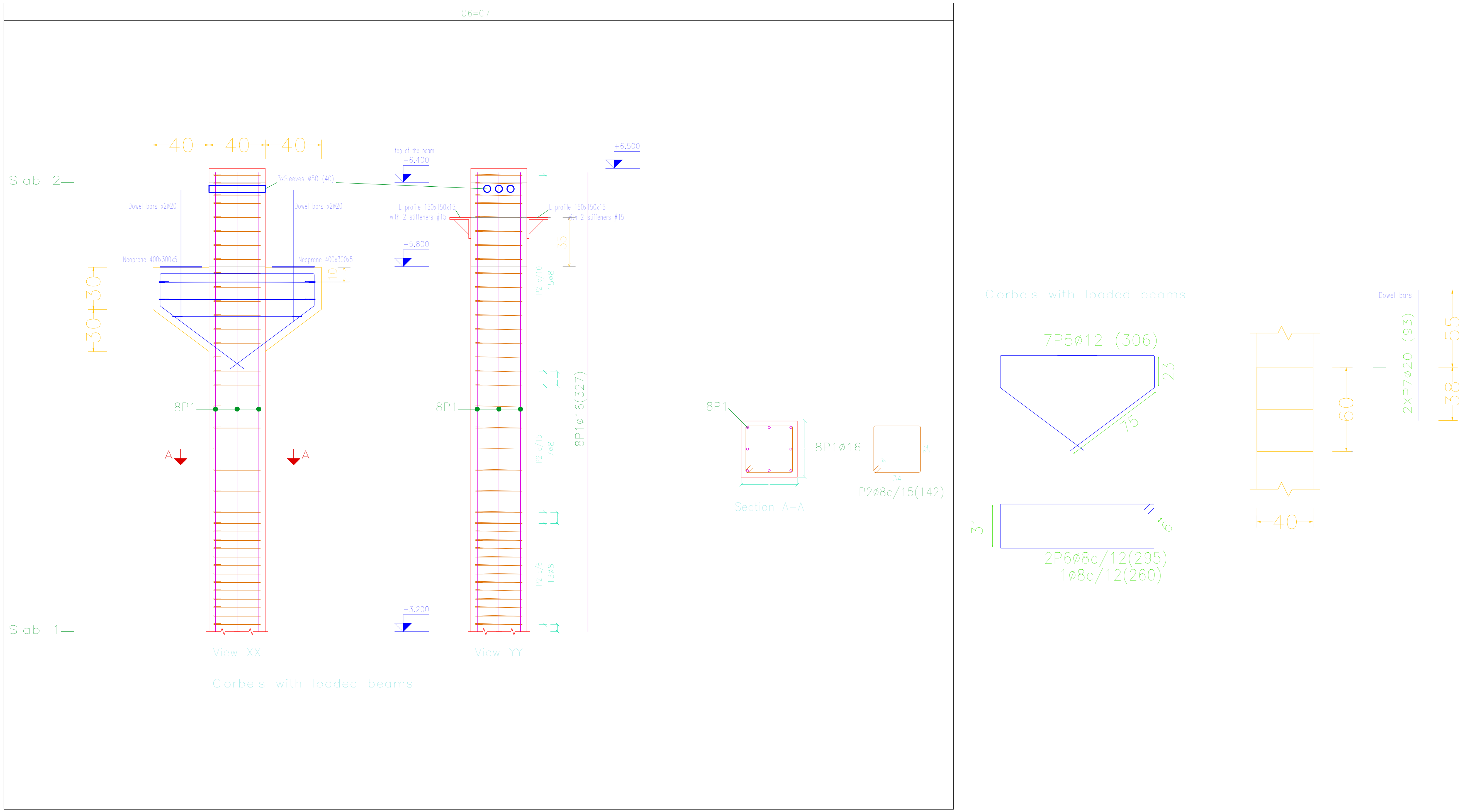

Grout for foundation: High strength – 60MPa
Concrete: fck=30, Yc=1.5
Reinforcement: B 500 SD, Ys=1.15

Concrete minimum cover: 3.00 cm
Maximum aggregate size: 20.0 mm

Scale: 1: 30

DRAWING	SCALE IN A3	DRAWING NUMBER
INTERNAL COLUMNS - FIRST FLOOR	1:30	21

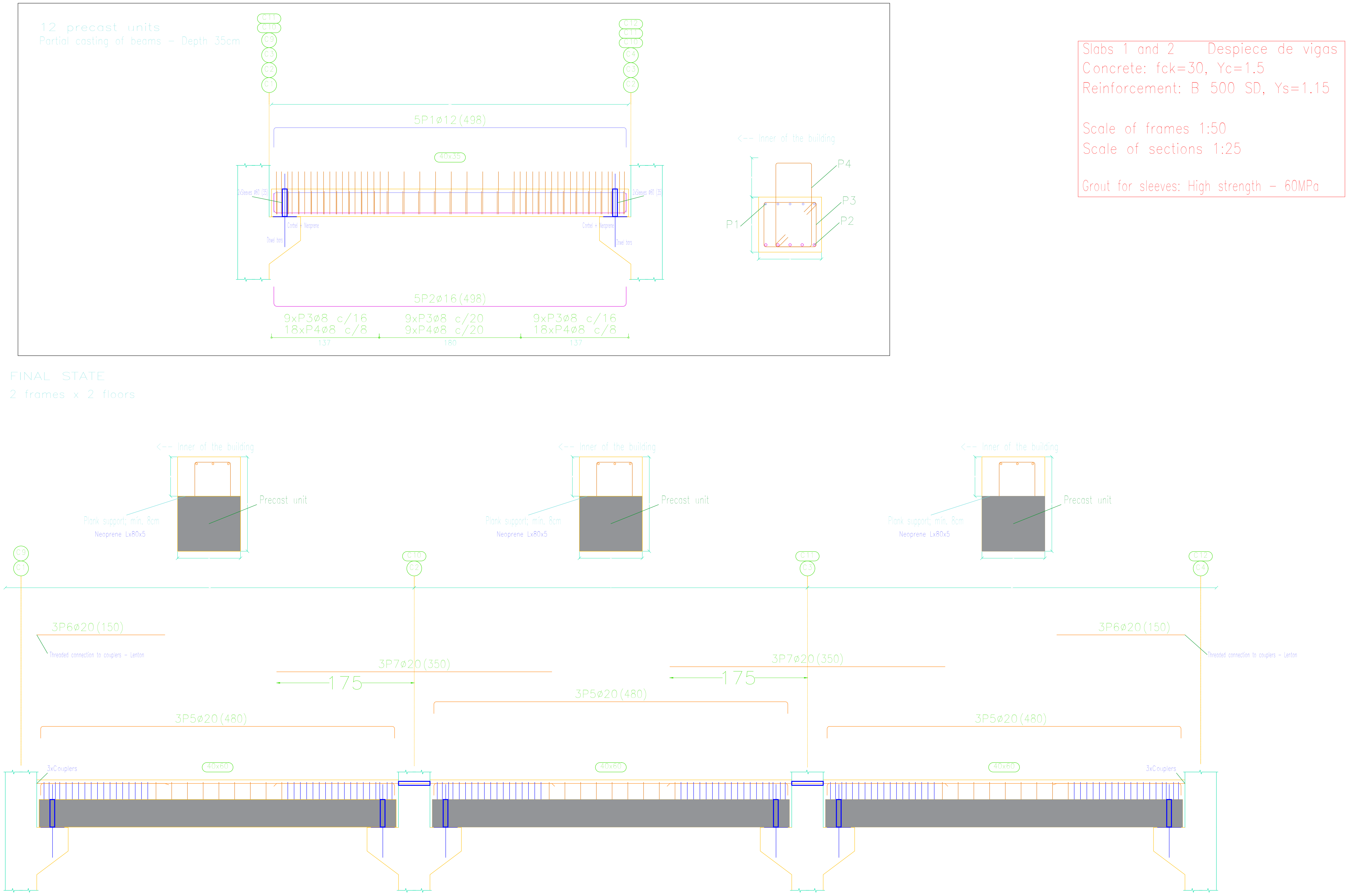

DRAWING	SCALE IN A3	DRAWING NUMBER
PRECAST BEAMS AND EXTERNAL FRAMES ASSEMBLAGE	MULTIPLE	22

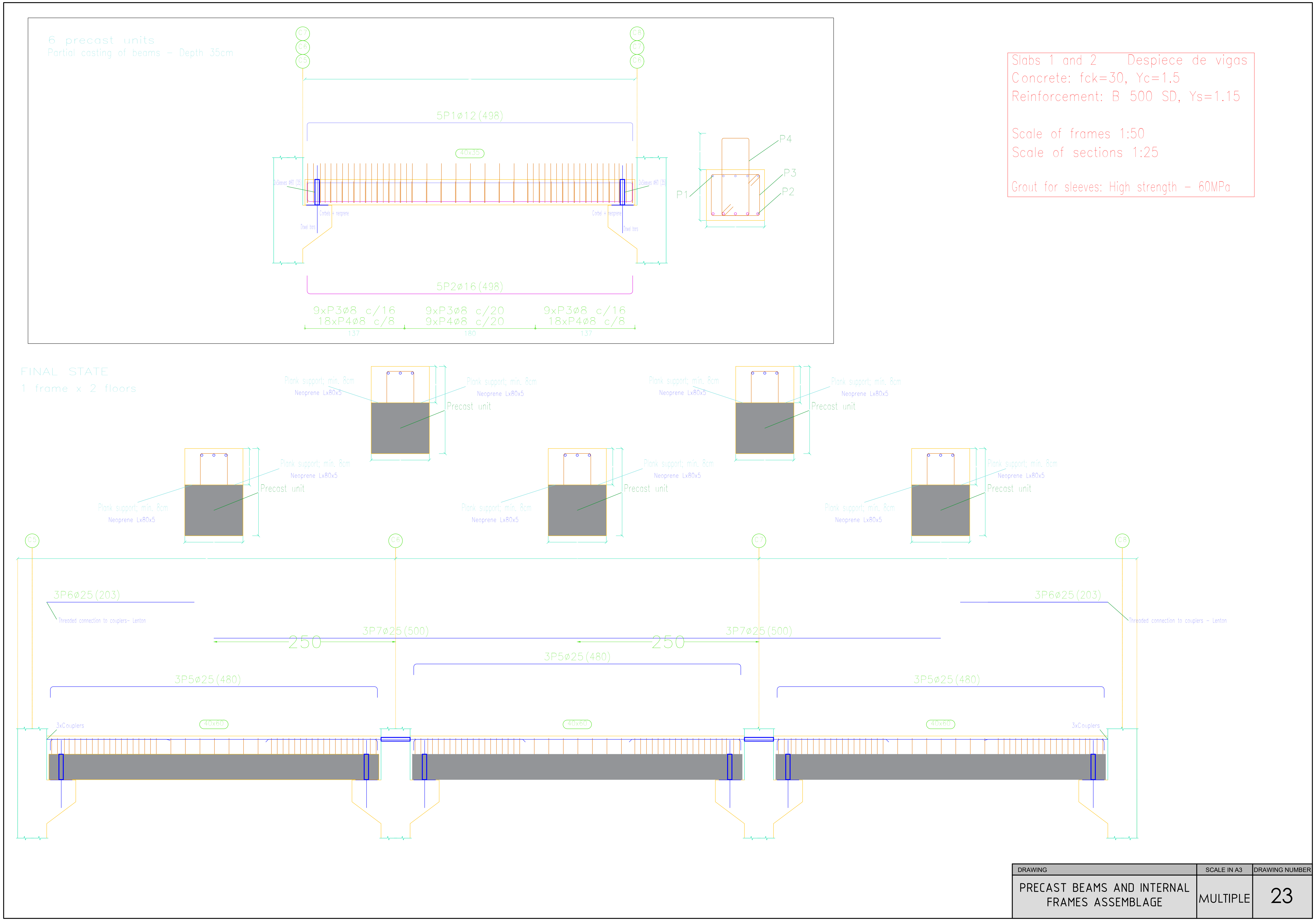
6 precast units
Partial casting of beams - Depth 35cm
5P1ø12(498)
5P2ø16(498)
9xP3ø8 c/16
18xP4ø8 c/8
9xP3ø8 c/20
9xP4ø8 c/20
9xP3ø8 c/16
18xP4ø8 c/8
P1
P2
P3
P4
Slabs 1 and 2 Despiece de vigas
Concrete: fck=30, Yc=1.5
Reinforcement: B 500 SD, Ys=1.15
Scale of frames 1:50
Scale of sections 1:25
Grout for sleeves: High strength - 60MPa
FINAL STATE
1 frame x 2 floors
Plank support: min. 8cm
Neoprene Lx80x5
Precast unit
3P6ø25(203)
Threaded connection to couplers- Lenton
3P7ø25(500)
250
3P5ø25(480)
3xCouplers
DRAWING
PRECAST BEAMS AND INTERNAL FRAMES ASSEMBLAGE
SCALE IN A3
MULTIPLE
DRAWING NUMBER
23

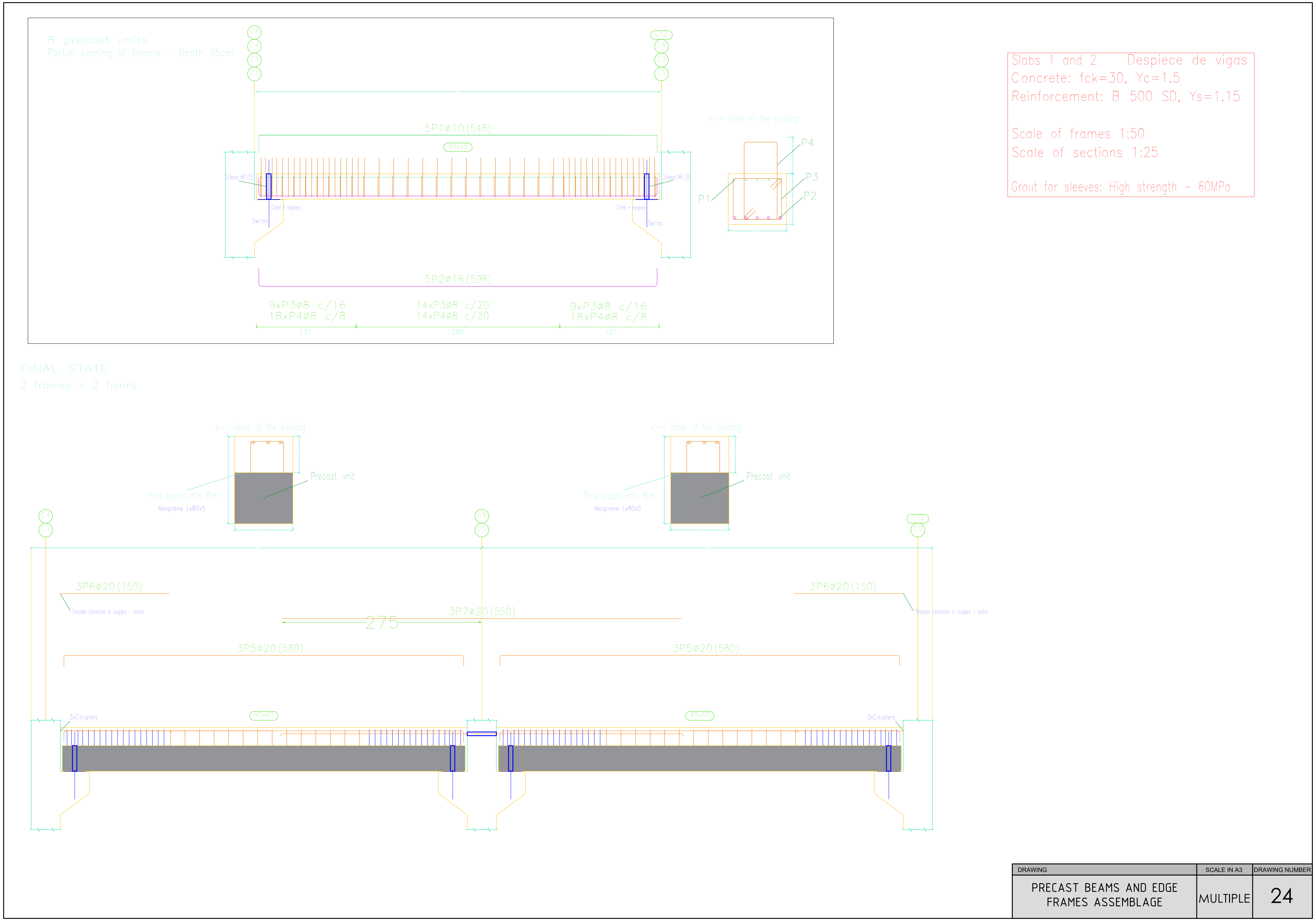
8 precast units
Partial casting of beams - Depth 35cm
5P1ø10 (548)
40x35
<-- Inner of the building
P1
P2
P3
P4
5P2ø16 (598)
9xP3ø8 c/16
18xP4ø8 c/8
14xP3ø8 c/20
14xP4ø8 c/20
9xP3ø8 c/16
18xP4ø8 c/8
137
280
137
Slabs 1 and 2 Despiece de vigas
Concrete: fck=30, Yc=1.5
Reinforcement: B 500 SD, Ys=1.15
Scale of frames 1:50
Scale of sections 1:25
Grout for sleeves: High strength - 60MPa
FINAL STATE
2 frames x 2 floors
<-- Inner of the building
Precast unit
Plank support, min. 8cm
Neoprene Lx80x5
3P6ø20 (150)
Threaded connection to couplers - Lenton
275
3P7ø20 (550)
3P5ø20 (580)
3P5ø20 (580)
40x60
40x60
3xCouplers
3xCouplers
DRAWING
PRECAST BEAMS AND EDGE FRAMES ASSEMBLAGE
SCALE IN A3
MULTIPLE
DRAWING NUMBER
24

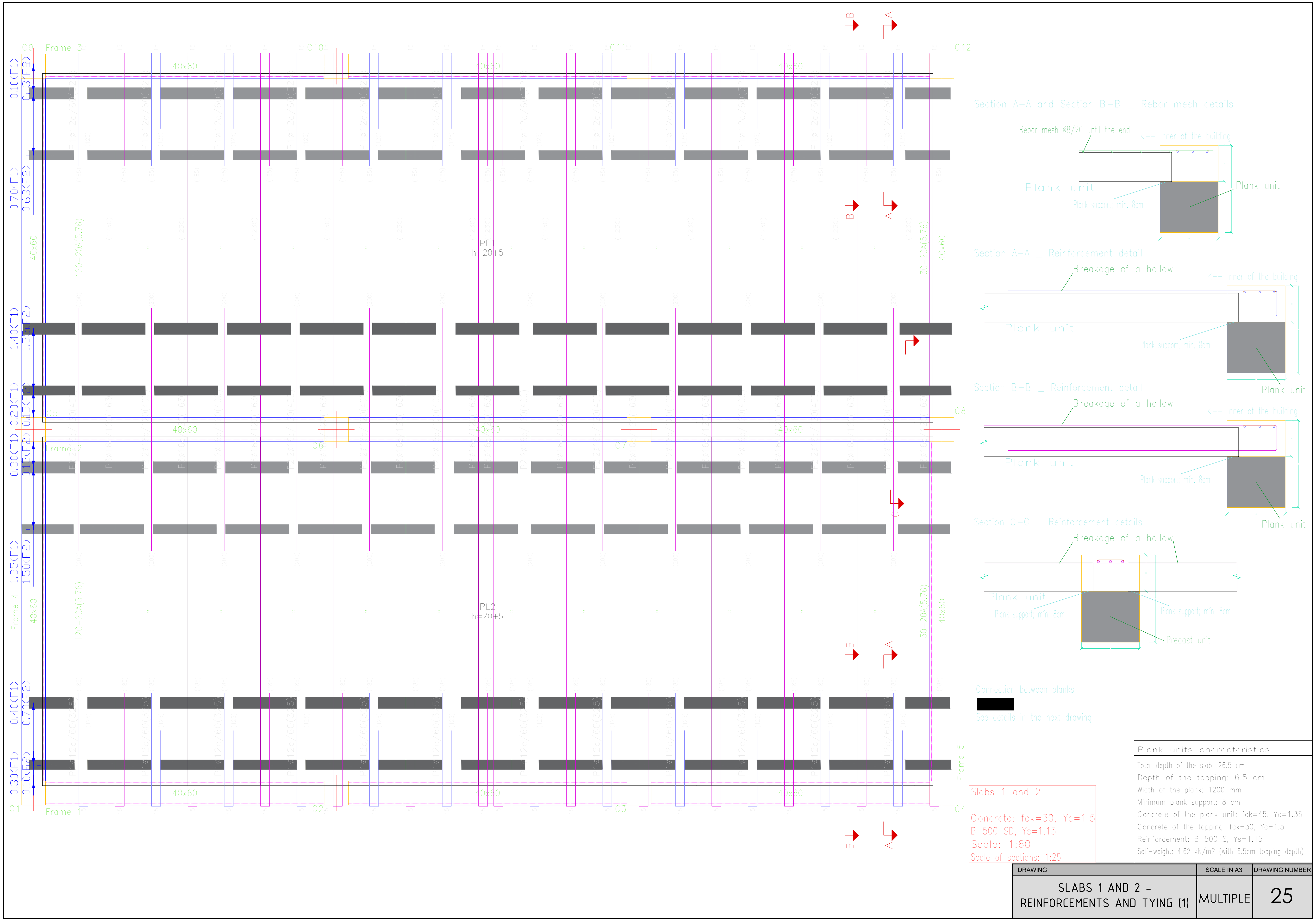
Section A-A and Section B-B _ Rebar mesh details
Rebar mesh Ø8/20 until the end
Plank unit
Section A-A _ Reinforcement detail
Breakage of a hollow
Plank unit
Section B-B _ Reinforcement detail
Breakage of a hollow
Plank unit
Section C-C _ Reinforcement details
Breakage of a hollow
Plank unit
Precast unit
PL1
h=20+5
PL2
h=20+5
Frame 1
Frame 3
Frame 4
Frame 5
Connection between planks
See details in the next drawing
Slabs 1 and 2
Concrete: fck=30, Yc=1.5
B 500 SD, Ys=1.15
Scale: 1:60
Scale of sections: 1:25
Plank units characteristics
Total depth of the slab: 26.5 cm
Depth of the topping: 6.5 cm
Width of the plank: 1200 mm
Minimum plank support: 8 cm
Concrete of the plank unit: fck=45, Yc=1.35
Concrete of the topping: fck=30, Yc=1.5
Reinforcement: B 500 S, Ys=1.15
Self-weight: 4.62 kN/m2 (with 6.5cm topping depth)
DRAWING
SLABS 1 AND 2 -
REINFORCEMENTS AND TYING (1)
SCALE IN A3
MULTIPLE
DRAWING NUMBER
25

Reinforcement details – Parallel to the plan unit main direction at the ends

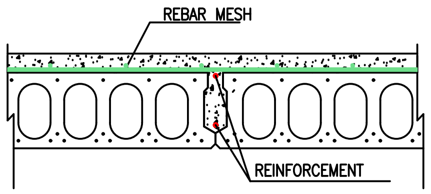

Reinforcement details – Parallel to the plan unit main direction at the center

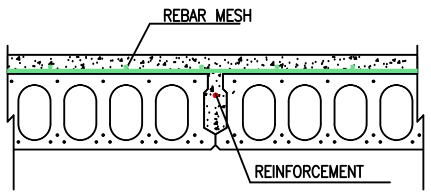

Reinforcement details – Parallel to the plank unit main direction – at the ends with breakage of a hollow

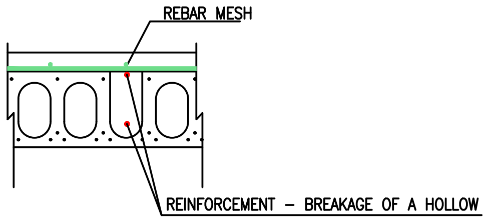

Reinforcement details – Parallel to the plank unit main direction – at the center with breakage of a hollow

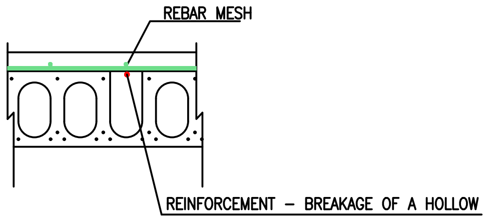

Reinforcement details of the connection between plank units

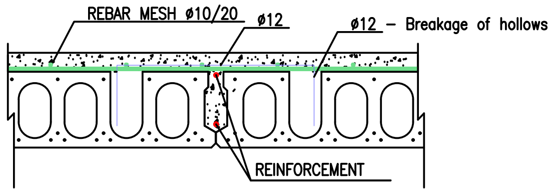

Connection between plank units – This symbol was used in the previous drawing

Detail of the lateral support of the plank unit

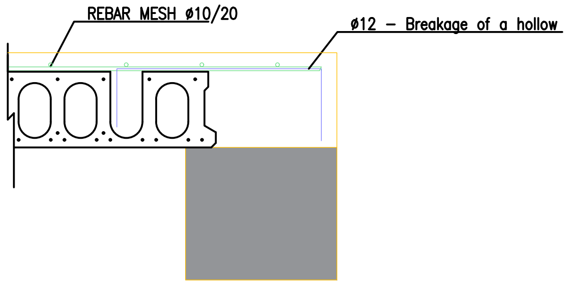

Connection between plank units – This symbol was used in the previous drawing

Scale: 1:20

DRAWING	SCALE IN A3	DRAWING NUMBER
SLABS 1 AND 2 – REINFORCEMENTS AND TYING (2)	1:20	26
